# A Tutorial on the Mechanisms of Group 9 Transition-Metal-Catalyzed Asymmetric Olefin Hydrogenation

**DOI:** 10.1021/acs.organomet.5c00512

**Published:** 2026-04-10

**Authors:** Lauren N. Mendelsohn, Paul J. Chirik

**Affiliations:** Department of Chemistry, 6740 Princeton University, Princeton, New Jersey 08544, United States

## Abstract

Asymmetric alkene hydrogenation catalyzed by group 9 transition-metal complexes has become one of the most widely used methods for producing enantio­enriched compounds, particularly single-enantiomer pharmaceuticals. This Tutorial is focused on the mechanistic aspects of asymmetric alkene hydrogenation mediated by group 9 transition-metal catalysts with an emphasis on elucidating reaction pathways and the origins of enantio­induction. Among these catalysts, bis­(phosphine)–rhodium­(I) complexes are the most extensively studied, due to their broad applicability and historical importance in the development of asymmetric hydrogenation. Early mechanistic studies provided strong support for an unsaturated pathway consistent with the Curtin–Hammett principle, in which the minor rhodium alkene diastereomer reacts more rapidly with H_2_ to yield the preferred enantiomer of the alkane. In contrast, later investigations with more electron-rich phosphines favored a metal dihydride pathway. Iridium catalysts bearing chiral bidentate phosphines have expanded the scope of asymmetric hydrogenation to minimally functionalized alkenes, and extensive experimental and computational studies support an Ir­(III)/Ir­(V) redox cycle. More recently, attention has shifted toward more earth-abundant cobalt catalysts, which can form both neutral and cationic complexes. Emerging evidence indicates that neutral cobalt catalysts operate through either non-redox Co­(II) metallacycle pathways or more rhodium-like Co­(0/II) redox cycling mechanisms.

## Graphical abstract

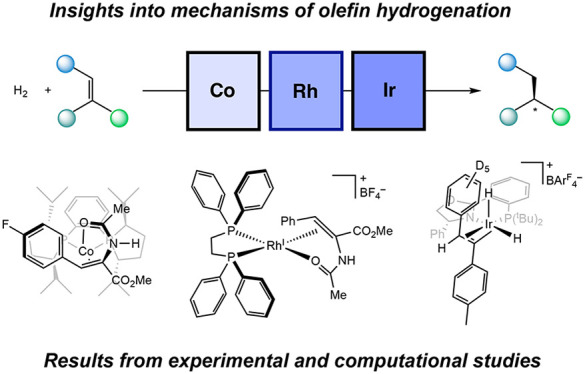

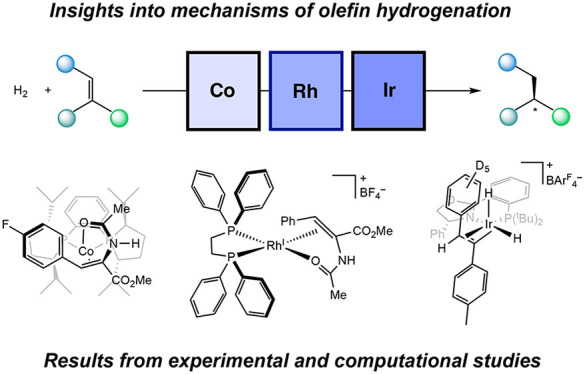

## Introduction

The asymmetric hydrogenation of olefins is one of the most powerful and widely used methods for the synthesis of enantio­enriched products.^1^ This is in part due to the discovery and implementation of highly active and enantio­selective catalysts, as well as the prevalence of C–H bonds in stereocenters in active pharmaceutical ingredients.^2^ In 1975, Knowles and co-workers reported the application of asymmetric alkene hydrogenation to an L-DOPA precursor and achieved 96% enantiomeric excess (*ee*) with [((*R*,*R*)-DIPAMP)­Rh­(COD)]­[BF_4_] (DIPAMP = (ethane-1,2-diyl)­bis­[(2-methoxyphenyl)­(phenyl)­phosphane]; COD = 1,4-cyclooctadiene) as the precatalyst (Scheme 1).^3^ This landmark discovery demonstrated the powerful role of molecular precatalysts in asymmetric synthesis, and since this initial report, scores of rhodium,^4^ iridium,^5^ and cobalt^6^ catalysts bearing chiral ligands have been applied to various enantio­selective hydrogenation reactions.^7^

**Scheme 1:**
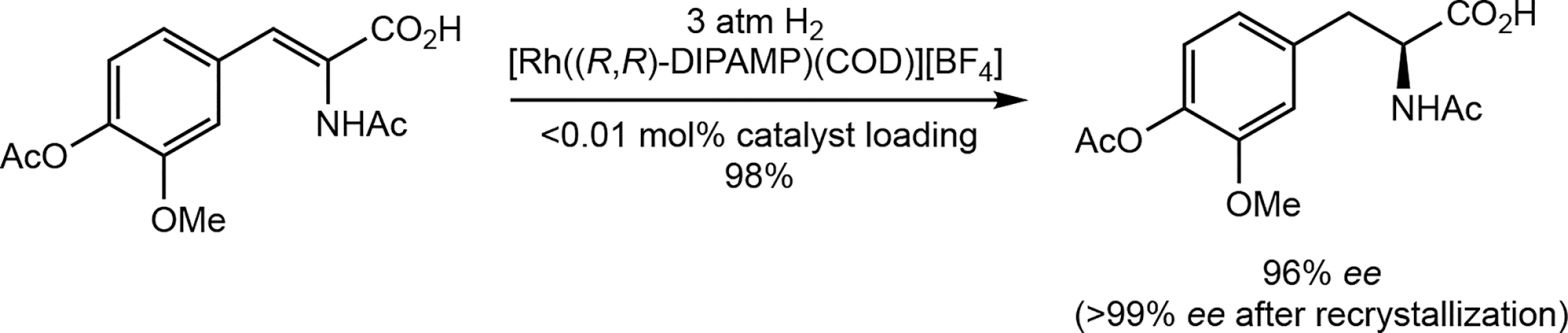
Seminal Synthetically Useful Example of Catalytic Asymmetric Hydrogenation: Asymmetric Hydrogenation of a Precursor to L-DOPA Using [((*R*,*R*)-DIPAMP)­Rh­(COD)]­[BF_4_] as the Precatalyst (Knowles, 1975 ^3^)

The development of new ligands for the realization of catalysts with improved activity and selectivity has been a continuing theme in the organometallic chemistry of asymmetric alkene hydrogenation.^8,9^ Over the past four decades, significant effort has also been devoted to elucidating the operative mechanisms for these important reactions. While most textbooks tend to focus on Halpern and Landis’s establishment of the “major–minor concept” as a demonstration of the Curtin–Hammett principle in the rhodium-catalyzed asymmetric hydrogenation of dehydroamino acids,^10^ continued experimental and computational studies have provided convincing evidence for other operative mechanisms depending on the metal, supporting ligand, type of substrate, and other conditions.

This Tutorial explores various mechanistic proposals that have been considered and evaluated for the asymmetric hydrogenation of alkenes using rhodium, iridium, and cobalt catalysts. Emphasis will be placed on catalysts bearing chiral bidentate phosphine ligands as these are the most common, synthetically useful, and widely used among the triad. This Tutorial is not intended to be a comprehensive review but rather a pedagogical guide for understanding the nuances and the mechanistic uncertainties still present in the field, especially as more earth abundant cobalt catalysts emerge. The examples from literature have been selected to illustrate various advancements in overall mechanistic understanding.

## Rhodium

### Brief Overview of Rhodium-Catalyzed Asymmetric Olefin Hydrogenation

Since the pioneering work of Knowles, bis­(phosphine) rhodium catalysts have been applied to the highly enantio­selective hydrogenation of a wide variety of olefins, including α,β-dehydroamino acids, enamides, itaconic acids, enol esters, and ethenephosphonates (Scheme 2A).^11^ It is generally accepted that the examples reported in the literature that proceed with high enantio­selectivity rely on “two-point binding” where the functional groups on the substrate facilitate coordination to the rhodium as well as facial discrimination.

**Scheme 2:**
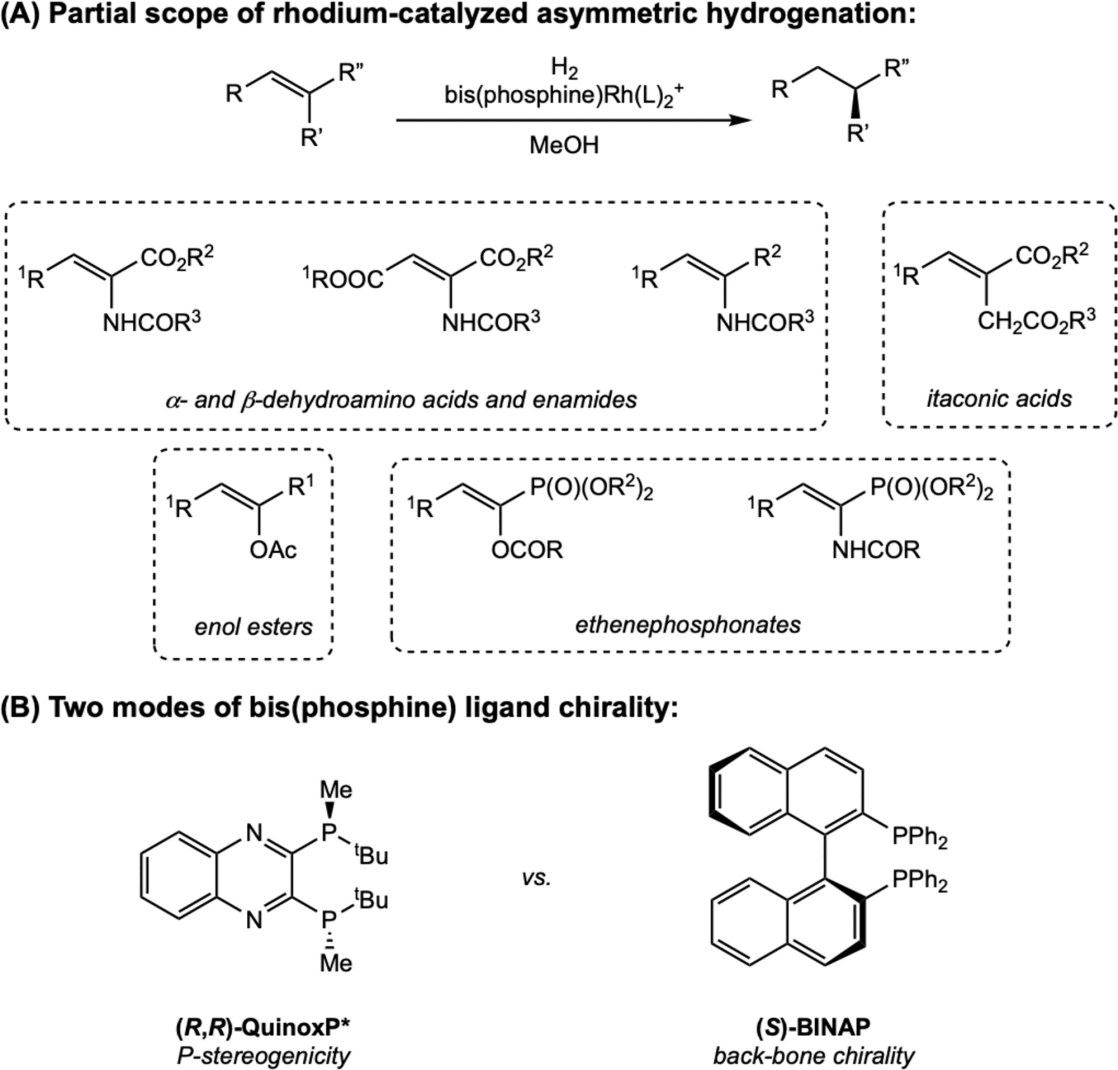
(A) General Scheme for Bis­(phosphine) Rhodium-Catalyzed Asymmetric Hydrogenation with Examples of Substrate Classes That Undergo Highly Enantioselective Hydrogenation and (B) Classes of Chiral Bis­(phosphine) Ligands with Selected Examples

Because of this historical precedent and the importance of these compounds in the pharmaceutical industry, the majority of mechanistic work has focused on the asymmetric hydrogenation of α,β-dehydroamino acids and enamides.^4^ Cationic, square-planar rhodium­(I) *d* ^8^ precatalysts bearing chiral bidentate phosphine ligands are most commonly used, supported by a chelating olefin, diene, or solvent molecules. A variety of chiral bis­(phosphine) ligands have been developed where the chiral element is on the phosphorus itself (P-stereogenicity) or derives from the carbon backbone (Scheme 2B).

The importance of the identity of the chiral bis­(phosphine) ligand will be a focus of this Tutorial, as it not only determines the stereochemical outcome of the reaction but also has a dramatic impact on the energetics and consequently, the preferred pathways and outcomes of asymmetric olefin hydrogenation. It is also important to note that many catalytic reactions using bis­(phosphine) rhodium catalysts operate in coordinating solvents such as methanol that also may play a significant role in the reaction mechanism.

### General Mechanistic Proposals: Unsaturated and Dihydride Pathways

Two principal, limiting pathways for the cationic bis­(phosphine) rhodium-catalyzed asymmetric hydrogenation of α,β-dehydroamino acids and enamides have been proposed. The distinguishing feature between these two pathways is the order in which the substrates, dihydrogen, or alkene interact with the rhodium catalyst (Scheme 3). In the unsaturated pathway, the alkene coordinates before H_2_ activation while in the dihydride pathway, H_2_ oxidative addition occurs before alkene coordination. Both pathways converge to a rhodium alkene dihydride complex, which undergoes migratory insertion to form a rhodium monohydride alkyl. Finally, C–H reductive elimination releases the alkane product and reinitiates the cycle. Hence, the key difference between these two pathways lies only in the initial stages of the mechanism. Because the enantio-determining step may be in these initial stages, this seemingly slight mechanistic nuance translates onto the overall success and utility of the process and is key for ligand and catalyst design. Support for each of these two mechanistic proposals will be discussed, including the factors that impact their prevalence.

**Scheme 3:**
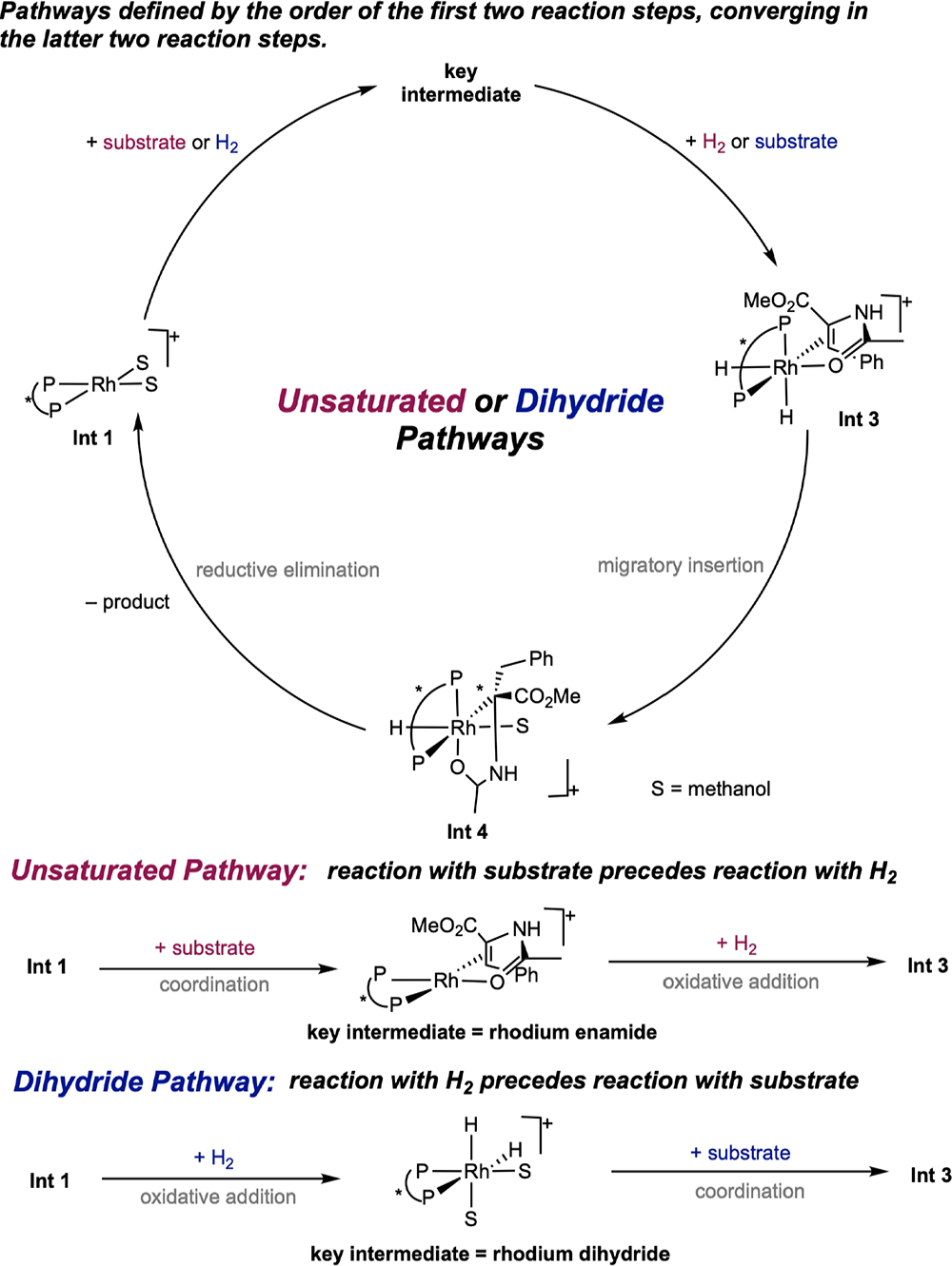
Two Limiting Mechanisms for the Bis­(phosphine) Rhodium-Catalyzed Asymmetric Hydrogenation of Prochiral Enamides: Unsaturated and Dihydride Pathways

### Evidence for Unsaturated Pathways in Rhodium-Catalyzed Asymmetric Hydrogenation

The earliest mechanistic studies for hydrogenation with cationic bis­(phosphine) rhodium catalysts support an unsaturated pathway. Experimental efforts were conducted independently by Halpern and Brown and relied on less donating achiral bis­(phosphine) ligands. It is proposed that the initial step of the cycle is coordination of the prochiral enamide to the [bis­(phosphine)­Rh­(S)_2_]^+^ precatalyst to form [bis­(phosphine)­Rh­(substrate)] resting states. The first evidence that the alkene substrate may coordinate to rhodium before dihydrogen coordination to rhodium, came from Halpern and co-workers in 1977 using an achiral ligand.^12^ Addition of hydrogen gas to a methanol solution of [(dppe)­Rh­(NBD)]­[BF_4_] (dppe = 1,2-bis­(diphenylphosphino)­ethane; NBD = norbornadiene) generated the rhodium bis­(solvento) complex, [(dppe)­Rh­(MeOH)_2_]­[BF_4_], rather than oxidative addition product (Scheme 4A). No evidence was obtained for the formation of rhodium hydrides. However, subsequent reaction of [(dppe)­Rh­(MeOH)_2_]­[BF_4_] with a variety of alkenes produced observable [(dppe)­Rh­(alkene)]­[BF_4_] complexes. This seminal report established the preference of cationic rhodium complexes bearing arylated bis­(phosphines) to engage with olefins, before H_2_, demonstrating the feasibility of the first steps of the unsaturated catalytic cycle.

**Scheme 4:**
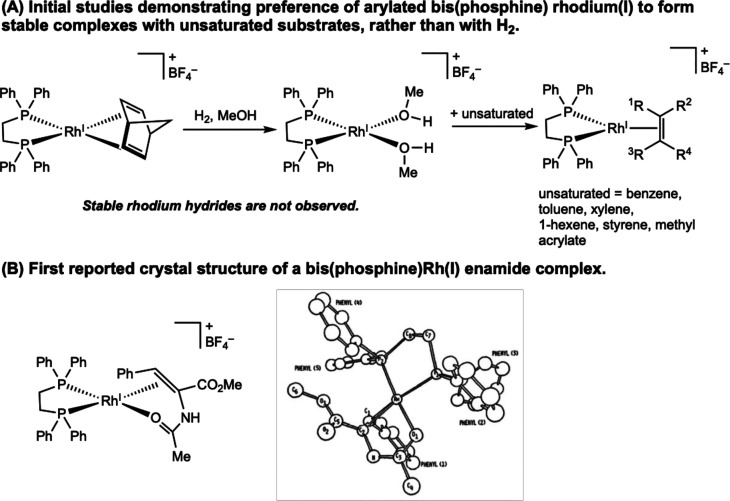
Stoichiometric Studies Using Achiral dppe Ligands: (A) Formation of Rhodium­(I) Solvento and Substrate Complexes (Halpern, 1977 ^12^) and (B) Representations of the Solid-State Structure of the Bis­(phosphine) Rhodium­(I) Enamide Complex, [(dppe)­Rh­(MAC)]­[BF_4_] (Halpern, 1979 ^13^)­^a^

a Panel B adapted with permission from ref 13. Copyright 1979 Elsevier.

In 1979, Halpern and co-workers provided a foundational example by determining the X-ray crystal structure of a cationic, achiral bis­(phosphine) rhodium–enamide complex, [(dppe)­Rh­(MAC)]­[BF_4_] (MAC = methyl (*Z*)-α-acetamido­cinnamate) (Scheme 4B).^13^ Two-point coordination of the alkene substrate through both the alkene C=C bond and the oxygen of the amide carbonyl to the rhodium was observed in the solid-state structure.

Introduction of a chiral, enantio­enriched bidentate phosphine results in the possible formation of two distinct diastereomeric rhodium–substrate complexes with a prochiral alkene. Through deuterium-labeling studies on the selective hydrogenation of alkynes to *cis*-olefins by cationic rhodium complexes bearing monodentate phosphine ligands, Schrock and Osborn had previously demonstrated that the transition state for hydrogen addition to the unsaturated bond occurs intramolecularly in a syn periplanar geometry.^14^ Addition of H_2_ to the bound face of the substrate in the so-called “pro-(*R*)” diastereomer should lead to the (*R*) enantiomer of the hydrogenation product, while addition of H_2_ to the bound face of the “pro-(*S*)” diastereomer should form the (*S*) product. In 1980, Halpern reported the first crystallographic characterization of a cationic rhodium–enamide complex with the determination of the solid-state structure of [(ChiraPhos)­Rh­(EAC)]­[BF_4_] (ChiraPhos = (2*R*,3*R*)-bis­(disphenyl­phosphino)­butane; EAC = ethyl (*Z*)-α-acetamido­cinnamate).^15^ Notably, the pro-(*S*) diastereomer was isolated and crystallographically characterized, while the catalytic asymmetric hydrogenation of EAC produced (*R*)-alkane with high enantiomeric excess (Scheme 5). Line broadening was observed in the ^31^P NMR spectrum, suggesting rapid exchange of free and bound alkenes.

**Scheme 5:**
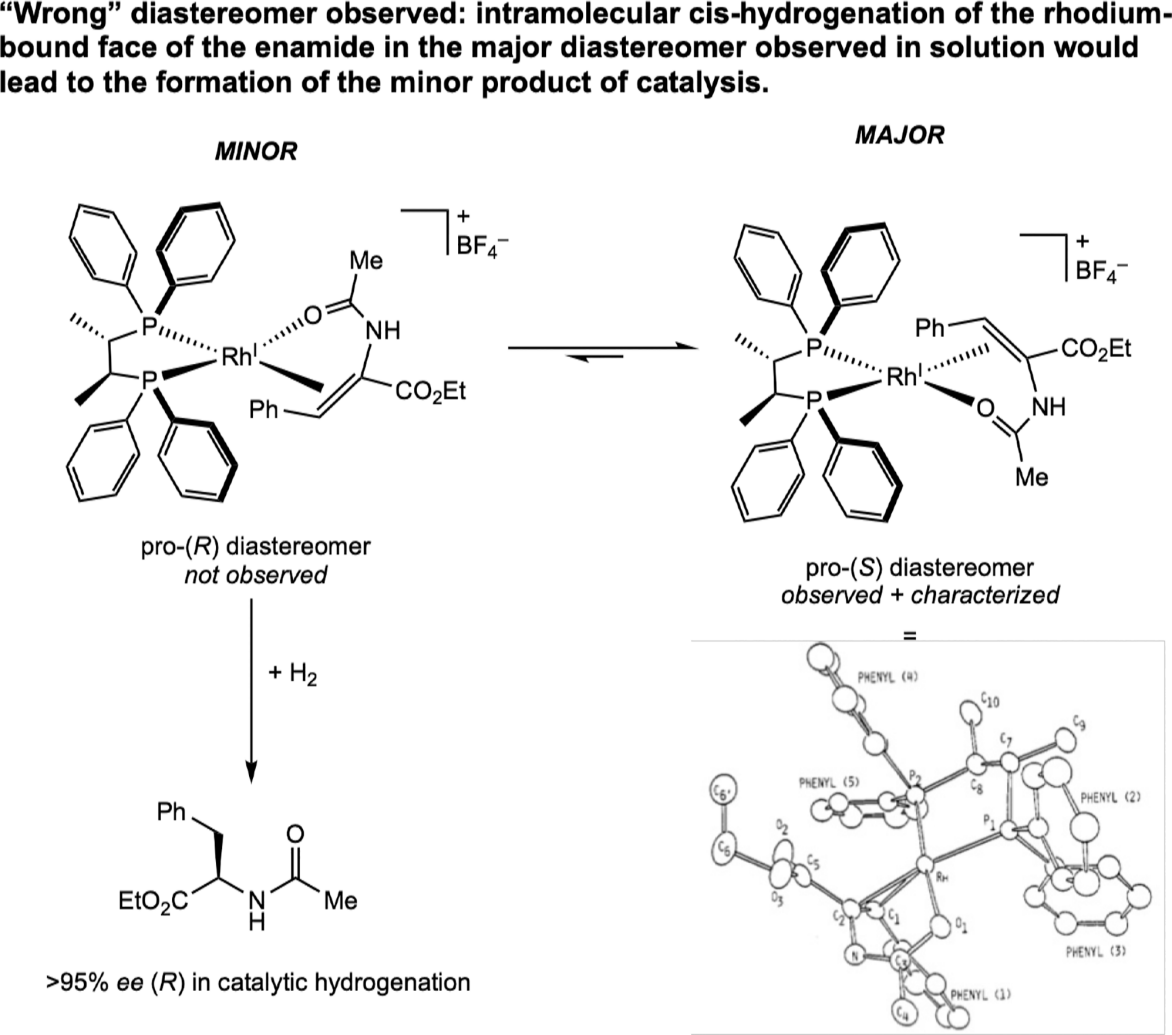
Observation and Solid-State Structure of the “Wrong” Diastereomer of the Chiral Bis­(phosphine) Rhodium­(I)^+^–Enamide Complex, [(ChiraPhos)­Rh­(EAC)]­[BF_4_] (Halpern, 1980 ^15^)­^a^

a Adapted from ref 15. Copyright 1980 American Chemical Society.

The authors proposed that isolation and crystallographic characterization of the “wrong” diastereomer of the rhodium complex could be a result of three possibilities: (1) that the unsaturated pathway is not actually operative, (2) that the minor diastereomer fortuitously crystallized, or (3) that the “wrong” diastereomer was the major intermediate in solution but reacts sufficiently slower with H_2_ so as to not influence the overall enantiomeric excess of the final product. The term “wrong” diastereomer refers to the case where hydrogenation of the face of the olefin coordinated to the metal center leads to formation of the minor product enantiomer from the catalytic experiment. Subsequent experimental evidence supported the third explanation. The solution-state circular dichroism (CD) spectrum of [(ChiraPhos)­Rh­(EAC)]­[BF_4_] matched the solid-state CD spectrum of the isolated pro-(*S*) crystals, suggesting that the “wrong” diastereomer was indeed the major rhodium compound in solution.^16^ Subsequent 2D NMR experiments that were not available at the time later confirmed these results.^17^

Observation of the “wrong” diastereomer in solution appeared to be general among this class of rhodium-catalyzed asymmetric hydrogenations. Using (1*R*,2*R*)-*trans*-1,2-bis­(diphenylphosphinomethyl)­cyclobutane as the supporting ligand, Brown and co-workers studied a variety of cationic rhodium–enamide diastereomers in solution by ^31^P NMR spectroscopy (Figure 1).^18^ No correlation was found between the ratio of rhodium–substrate diastereomers in solution and the *ee* of the product formed from the catalytic hydrogenation reaction. Isolation of [(DIPAMP)­Rh­(MAC)]­[BF_4_][Bibr ref19] and [(^Me^DuPhos)­Rh­(MAC)]­[BF_4_][Bibr ref20] (^Me^DuPhos = (+)-1,2-bis­[(2*S*,5*S*)-2,5-dimethylphospholano]­benzene) also supported formation of the “wrong” diastereomer as the major isomer of the rhodium compound in solution. These studies suggested that the ratio of diastereomers in solution was not the eventual source of chirality. The observation of the major rhodium–enamide diastereomer in solution leading to the minor amide product enantiomer became known as the “major–minor” or the “anti-lock-and-key” concept and widely appears in many organometallic chemistry and physical organic chemistry textbooks in the context of the Curtin–Hammett principle.

**Figure 1:**
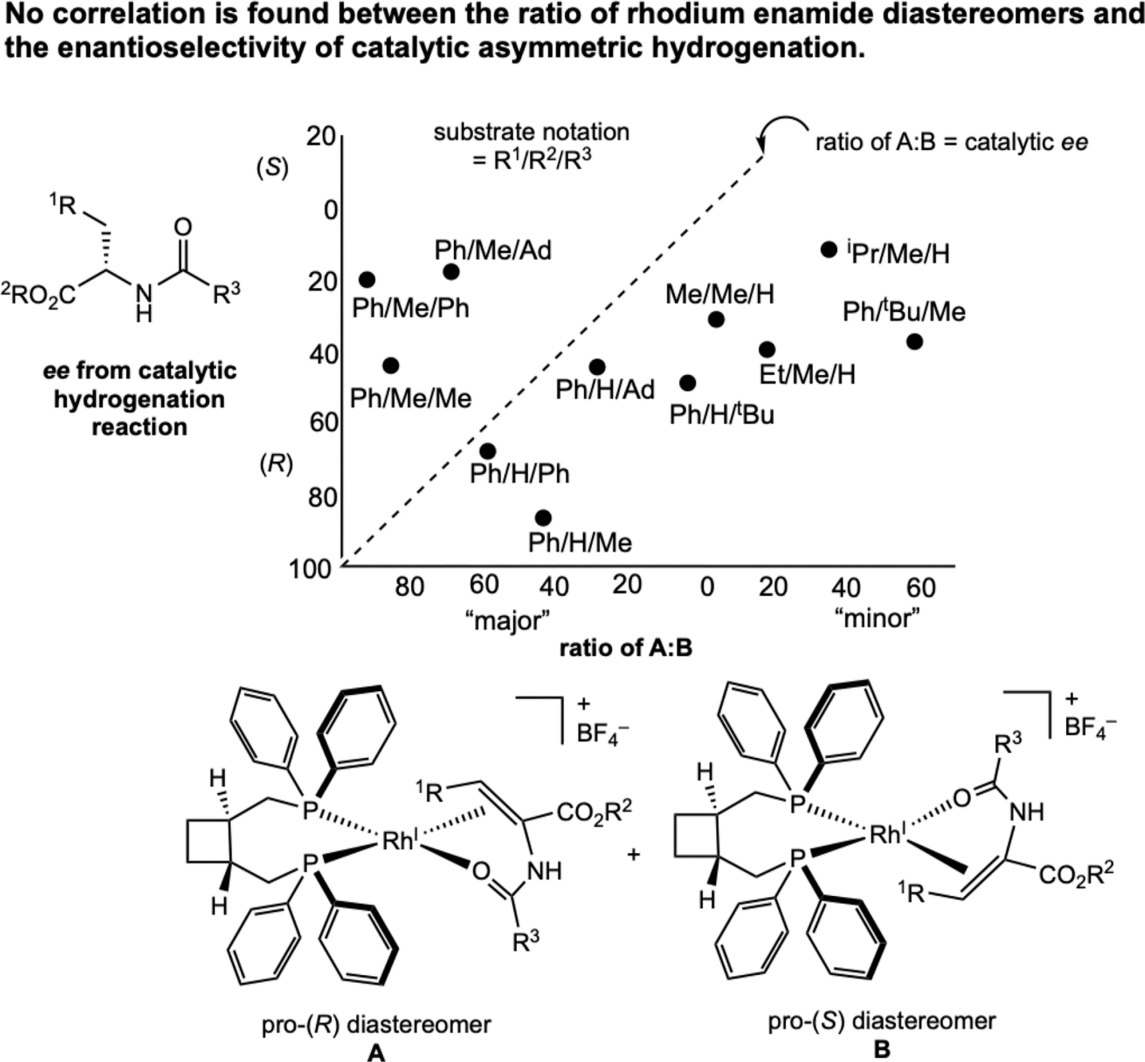
Plot of the diastereomer ratio vs enantiomeric excess for a series of [((1*R*,2*R*)-*trans*-1,2-bis­(diphenyl­phosphino­methyl)­cyclo­butane)­Rh­(substrate)]­[BF_4_] compounds, establishing little to no correlation between the equilibrium diastereomer ratio in solution and product chirality (Brown, 1979 ^18^). Adapted with permission from ref 18. Copyright 1979 Elsevier.

Importantly, there have also been examples of rhodium–substrate complexes where the “right” diastereomer was observed in solution. One example was reported by Heller in 2005, where [(DIPAMP)­Rh­(enamide)]­[BF_4_] complexes bearing β-aminoacrylates primarily formed the diastereomer with the same (pro) chirality as the product of catalytic hydrogenation reaction.^21^ However, even in this case, the ratio of diastereomers in solution did not correspond to the eventual product enantio­selectivity, demonstrating that enantio­selectivity is a product of subsequent reaction steps, not just the diastereomer ratio.

Substrate coordination to [bis­(phosphine)­Rh­(S)_2_]^+^ must therefore be reversible. As mentioned previously, ^31^P NMR spectroscopy established exchange between rhodium–substrate complexes and the free enamide in solution, supporting reversible coordination. In 1987, Landis and Halpern quantified the equilibrium constant for MAC coordination to [(DIPAMP)­Rh­(MeOH)_2_]­[BF_4_] and suggested a dissociative pathway for diastereomer interconversion, involving dissociation and re-coordination of the substrate facilitated through an intermediate rhodium­(I) bis­(solvento) complex.^22^ Later work by Brown published in 1993 suggested a rate 5 times faster for the intramolecular diastereomer interconversion of [(DIPAMP)­Rh­(methyl-(*Z*)-α-benzamido­cinnamate)]­[BF_4_] in methanol.^20^ A ^31^P,^31^P EXSY NMR study by Von Philipsborn and co-workers demonstrated that both intramolecular and intermolecular types of interconversion are operative with [(ChiraPhos)­Rh­(MAC)]­[BF_4_] and free MAC in solution (Scheme 6).^23^ In the intramolecular pathway, exchange occurs by dissociation of the C=C bond of the enamide (the oxygen of the amide carbonyl remains bound to the rhodium) in exchange for a single solvent molecule, followed by reassociation of the C=C bond. Conversely, in the intermolecular pathway, both the C=C bond and the oxygen of the amide carbonyl dissociate from the rhodium in exchange for two solvent molecules, followed by re-coordination of a substrate molecule. The relative rates of the intra- and intermolecular pathways were solvent dependent, where the intermolecular process was favored in methanol or acetone, while the intramolecular pathway was favored in less coordinating solvents such as CH_2_Cl_2_. In 1995, Brown reported an EXSY NMR study that established predominantly intramolecular exchange for [((2-methoxyphenyl)-P-phenyl-P-(2′-diphenyl-1-phosphino)­ethylphosphine)­Rh­(MAC)]­[BF_4_] in methanol.^24^ In summary, the coordination of enamide substrates to bis­(phosphine) rhodium­(I) complexes is reversible and is solvent dependent, relying on the assistance of at least one intermediary binding solvent molecule following dissociation of the C=C bond.

**Scheme 6:**
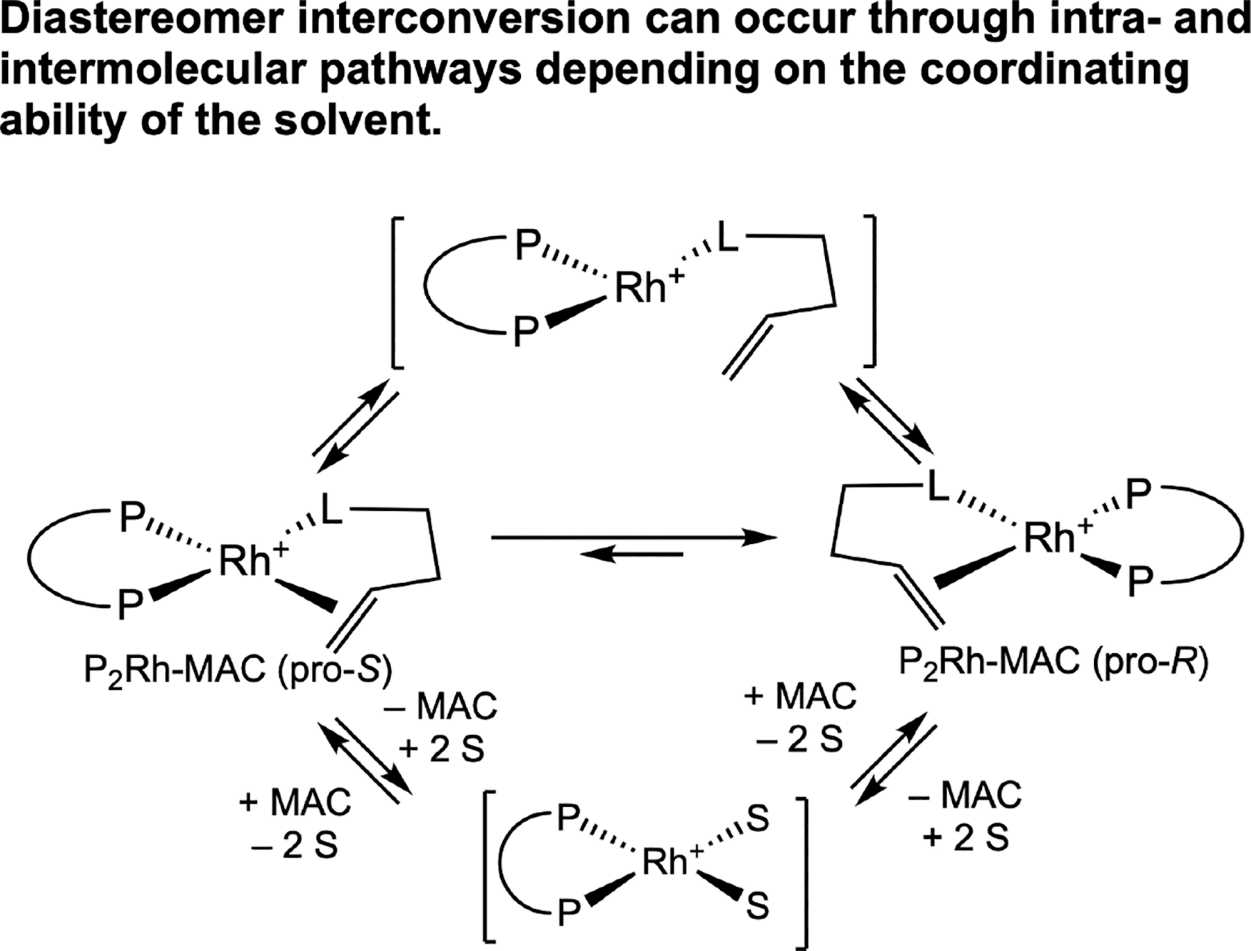
Intra- and Intermolecular Pathways for Rhodium–Enamide Diastereomer Interconversion (von Philipsborn, 1993 ^23^)­^a^

a Adapted with permission from ref 23. Copyright 1993 John Wiley and Sons.

Having established a deeper understanding of the coordination of enamides to cationic bis­(phosphine) rhodium­(I) complexes, the reaction of the diastereomers with H_2_ was of interest. If the ratio of rhodium–substrate diastereomers in solution is not the source of eventual product chirality, then the enantio­selectivity of the reaction must be determined at a subsequent stage in the catalytic cycle. To explore the succeeding reaction steps, Halpern and co-workers passed hydrogen gas through a methanol solution of [(dppe)­Rh­(MAC)]­[BF_4_] at −78 °C.^25^ Rather than a rhodium dihydride alkene complex, the direct product of H_2_ oxidative addition, the rhodium monohydride alkyl arising from migratory insertion was observed (Figure 2A). This result supports a fast migratory insertion step and that the putative dihydride alkene complex is very reactive. In fact, no bis­(phosphine) rhodium dihydride alkene complex was observed until 1997, when Bargon and co-workers used a diphosphite ligand and an itaconic acid derivative at low temperature to observe this elusive intermediate using parahydrogen-induced polarization NMR methods (Figure 2B).^26^

**Figure 2:**
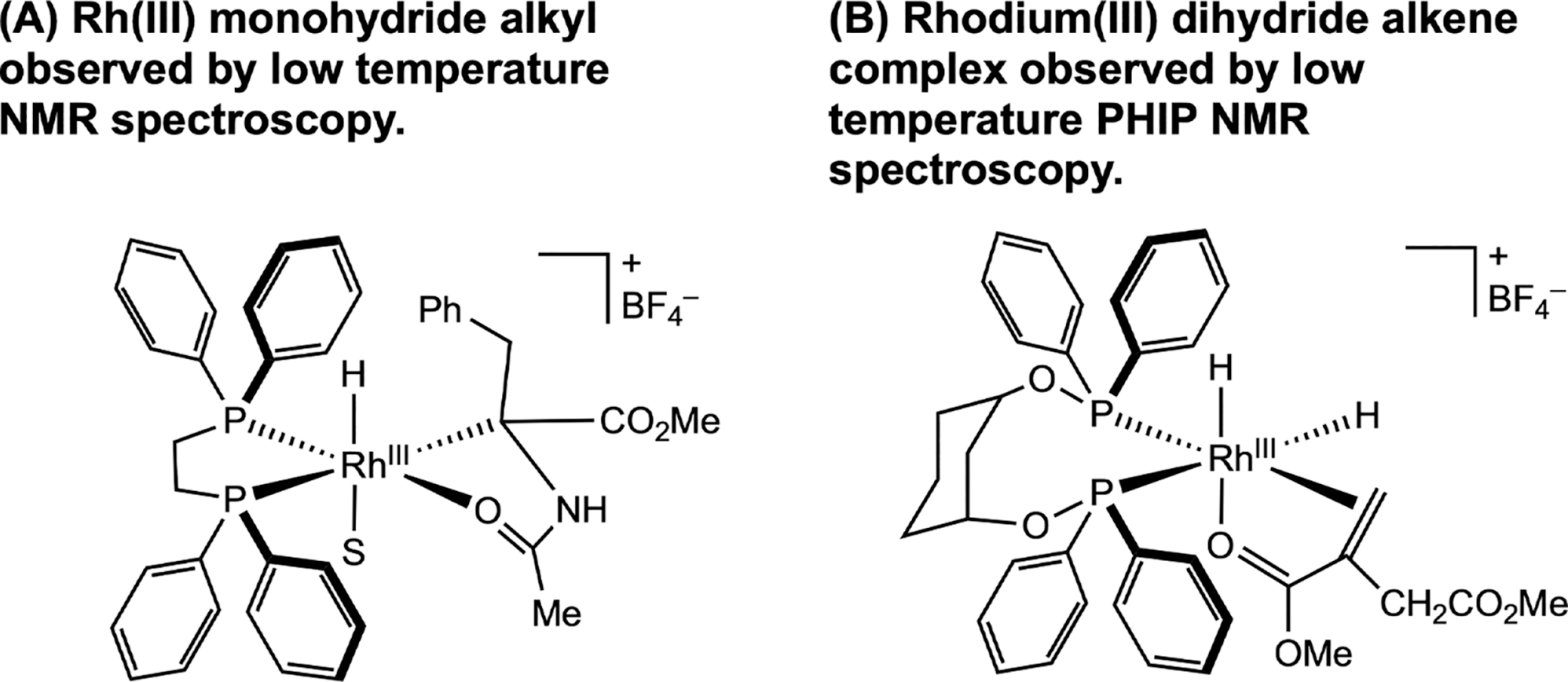
(A) Bis­(phosphine) rhodium monohydride alkyl complex observed at −78 °C by NMR spectroscopy (Halpern, 1980 ^25^). (B) Rhodium dihydride–alkene complex observed by low-temperature parahydrogen-induced polarization (PHIP) NMR methods (Bargon, 1997 ^26^).

In 1980, Halpern studied the kinetics of catalytic MAC hydrogenation using [(ChiraPhos)­Rh]^+^ and found a rate law equal to k­[H_2_]­[(ChiraPhos)­Rh­(EAC)^+^], indicating that oxidative addition of H_2_ to [(ChiraPhos)­Rh­(EAC)]­[BF_4_] is the rate-determining step.^27^ In this case, the *major* catalyst–substrate diastereomer in solution led to the *minor* hydrogenation product. Therefore, the minor rhodium–EAC diastereomer must be sufficiently more reactive toward H_2_ that it dominates the enantio­selectivity of the reaction (Figure 3). Consequently, at higher H_2_ pressures, the *ee* should be diminished and eventually be reversed as the rate of H_2_ oxidative addition to the “wrong” diastereomer is increased, a feature that had in fact been observed in such hydrogenation reactions.

**Figure 3:**
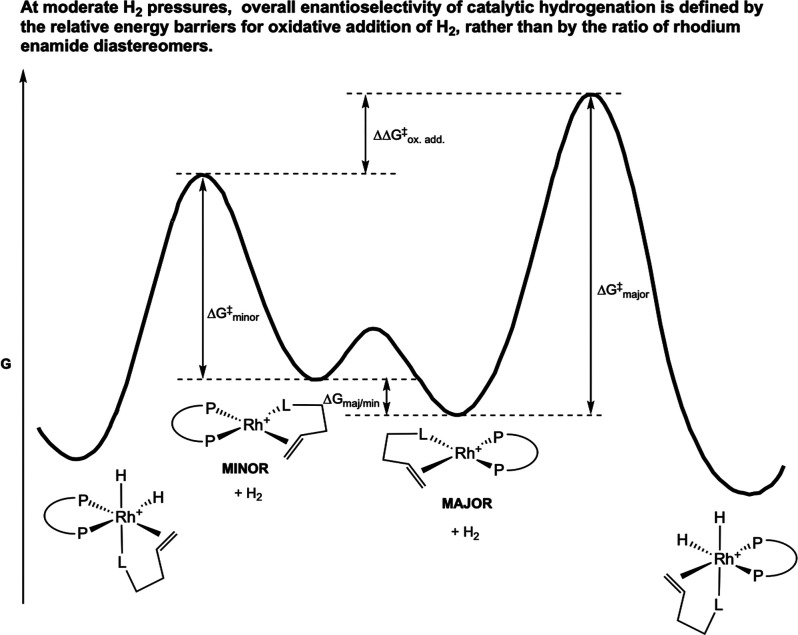
Curtin–Hammett principle as the foundation for the “major–minor” concept in the unsaturated pathway in rhodium-catalyzed asymmetric alkene hydrogenation (Halpern, 1987 ^27^).

This phenomenon was studied more thoroughly by Landis and Halpern in 1987.^22^ Kinetic measurements at low H_2_ pressure also established a first order dependence on the pressure of dihydrogen and rhodium concentration, with a zeroth order dependence on the substrate concentration. Such a rate law supports H_2_ oxidative addition as the rate-determining step. At higher hydrogen pressures, the rate of H_2_ oxidative addition to the minor (“right”) diastereomer plateaued (saturation kinetics), while that of the major (“wrong”) diastereomer continued to increase, leading to a decrease in their relative rates of product formation and a decrease in the overall reaction enantio­selectivity. Derivatization of rate laws for “low” (eq 1), “intermediate” (eq 2), and “high” (eq 3) pressure regimes showed that in the limit of infinitely high H_2_ pressures, the rate-determining step would become the initial substrate coordinating step, and the product chirality should entirely reverse. The decrease in *ee* at higher pressures was a result of the “intermediate” pressure regime. At any experimentally accessible H_2_ pressures, the rate-determining step of the reaction is the oxidative addition of H_2_ to the rhodium–substrate complex, suggesting that enantio­selectivity occurs at or following this step.

Low pressure:

1 $$\begin{array}{l} \mathrm{total}\;\mathrm{rate}=-\frac{d\left[H_{2}\right]}{dt}=\frac{(k_{2}^{\mathrm{maj}}K_{1}^{\mathrm{maj}}\left[H_{2}\right]+k_{2}^{\min}K_{1}^{\min}\left[H_{2}\right]{\left[\mathrm{Rh}\right]}_{\mathrm{tot}}}{K_{1}^{\mathrm{maj}}+K_{1}^{\min}}\\ \frac{d\left[R\mathrm{‐product}\right]}{dt}=\frac{k_{2}^{\mathrm{maj}}K_{1}^{\mathrm{maj}}\left[H_{2}\right]{\left[\mathrm{Rh}\right]}_{\mathrm{tot}}}{K_{1}^{\mathrm{maj}}+K_{1}^{\min}}\\ \frac{d\left[S\mathrm{‐product}\right]}{dt}=\frac{k_{2}^{\min}K_{1}^{\min}\left[H_{2}\right]{\left[\mathrm{Rh}\right]}_{\mathrm{tot}}}{K_{1}^{\mathrm{maj}}+K_{1}^{\min}} \end{array}$$

Intermediate H_2_ pressure:

2 $$\begin{array}{l} \frac{d\left[R\mathrm{‐product}\right]}{dt}=k_{2}^{\mathrm{maj}}\left[H_{2}\right]{\left[\mathrm{Rh}\right]}_{\mathrm{tot}}\\ \frac{d\left[S\mathrm{‐product}\right]}{dt}=\frac{k_{2}^{\min}k_{1}^{\min}\left[H_{2}\right]{\left[\mathrm{Rh}\right]}_{\mathrm{tot}}}{K_{1}^{\mathrm{maj}}\left(k_{-1}^{\min}+k_{2}^{\min}\left[H_{2}\right]\right)}\\ \frac{{\left[\mathrm{Rh}\right]}_{\mathrm{tot}}}{\frac{d\left[S\mathrm{‐product}\right]}{dt}}=\frac{K_{1}^{\mathrm{maj}}k_{-1}^{\min}}{k_{1}^{\min}k_{2}^{\min}\left[H_{2}\right]}+\frac{K_{1}^{\mathrm{maj}}}{k_{1}^{\min}} \end{array}$$

High H_2_ pressure:

3 $$\begin{array}{l} \mathrm{total}\;\mathrm{rate}=-\frac{d\left[H_{2}\right]}{dt}=k_{1}^{\mathrm{maj}}{\left[\mathrm{Rh}\right]}_{\mathrm{tot}}\left[\mathrm{mac}\right]+k_{1}^{\min}{\left[\mathrm{Rh}\right]}_{\mathrm{tot}}\left[\mathrm{mac}\right]\\ \frac{\left[S\mathrm{‐product}\right]}{\left[R\mathrm{‐product}\right]}=\frac{k_{1}^{\min}}{k_{1}^{\mathrm{maj}}} \end{array}$$

To gain more insight into the nature of the reaction between rhodium–substrate complexes and H_2_, deuterium-labeling experiments were performed. In 1982, Brown and co-workers measured both the H_2_/D_2_ kinetic isotope effect (KIE) and the HD partitioning ratio for the hydrogenation of (*Z*)-acetamido­cinnamic acid with [(dppe)­Rh­(NBD)]­[BF_4_].^28^ An H_2_/D_2_ KIE of 1.23 (20 °C) was measured, whereas the catalytic reaction with HD led to a 1.36:1.00 ratio of deuterium in the α:β-positions (Scheme 7A). Due to the similarity of these values, the authors concluded only that these results suggested an “irreversible hydride step”. The preference for deuterium incorporation in the α-position is consistent with a 2,1-insertion step.

**Scheme 7:**
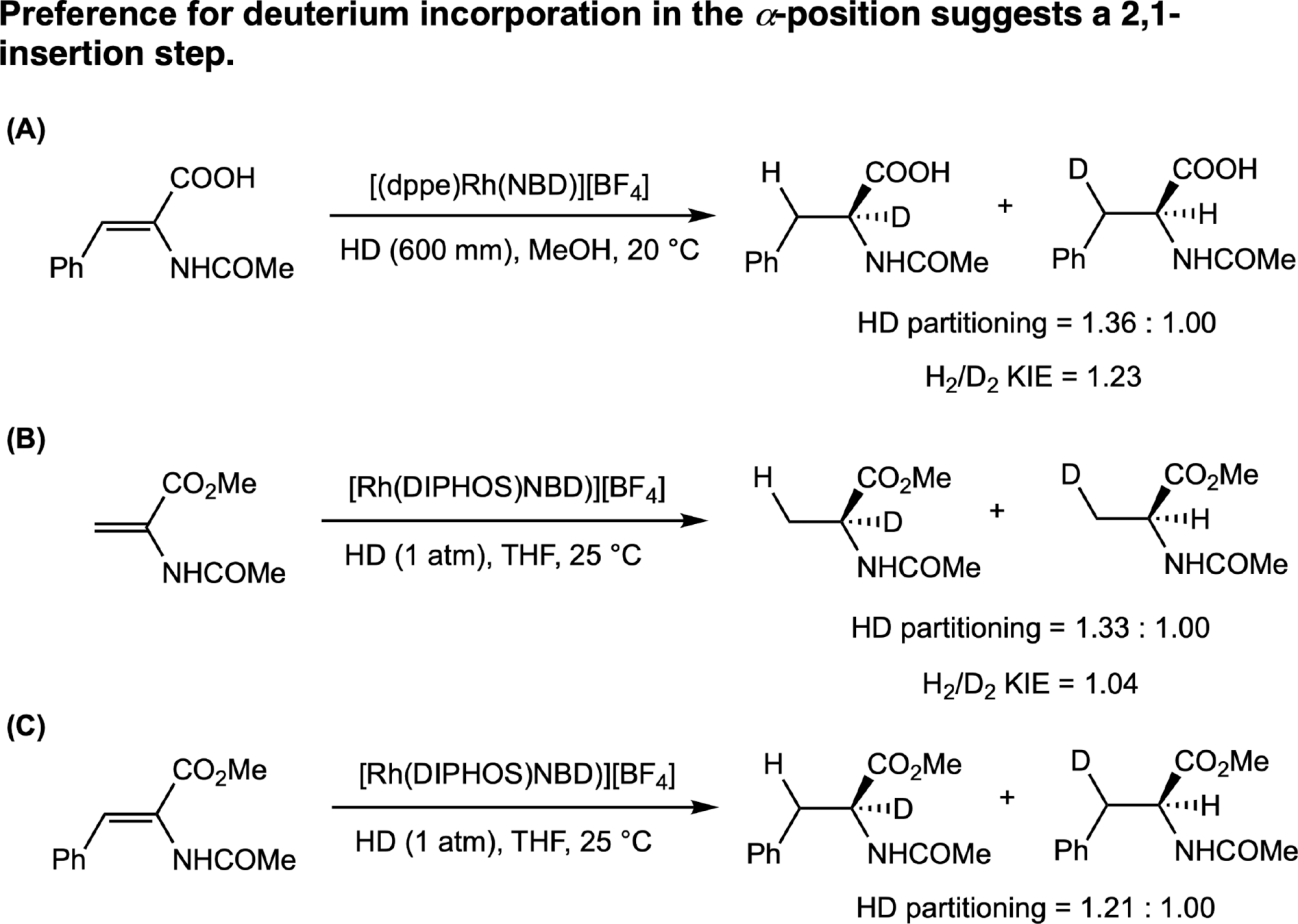
Combined HD Labeling Experiments (Brown, 1982,^28^ and Landis, 1998 ^29^)

A more thorough study was performed by Landis in 1998.^29^ Using mixtures of H_2_ and D_2_ gas added into the same tube and assayed by mass spectroscopic headspace analysis, Landis measured an average H_2_/D_2_ KIE of 1.04 for the catalytic hydrogenation of MAA (MAA = methyl 2-acetamido­acrylate) with [(dppe)­Rh­(NBD)]­[BF_4_] at 25 °C. The catalytic reaction of HD with MAA and MAC yielded a preference for deuterium in the α-position in a 1.33:1.00 and 1.21:1.00 ratio, respectively (Scheme 7B and C). Four possibilities for the nature of the reaction of the rhodium–substrate complex with dihydrogen were considered: (1) oxidative hydride transfer, (2) reversible oxidative addition and irreversible migratory insertion, (3) irreversible oxidative addition and reversible insertion, and (4) irreversible oxidative addition and irreversible insertion (Scheme 8). In an oxidative hydride-transfer step, alkene reduction occurs in a metathesis-type fashion directly into the σ-bound dihydrogen, concomitant with a two-electron oxidation of the metal center.

**Scheme 8:**
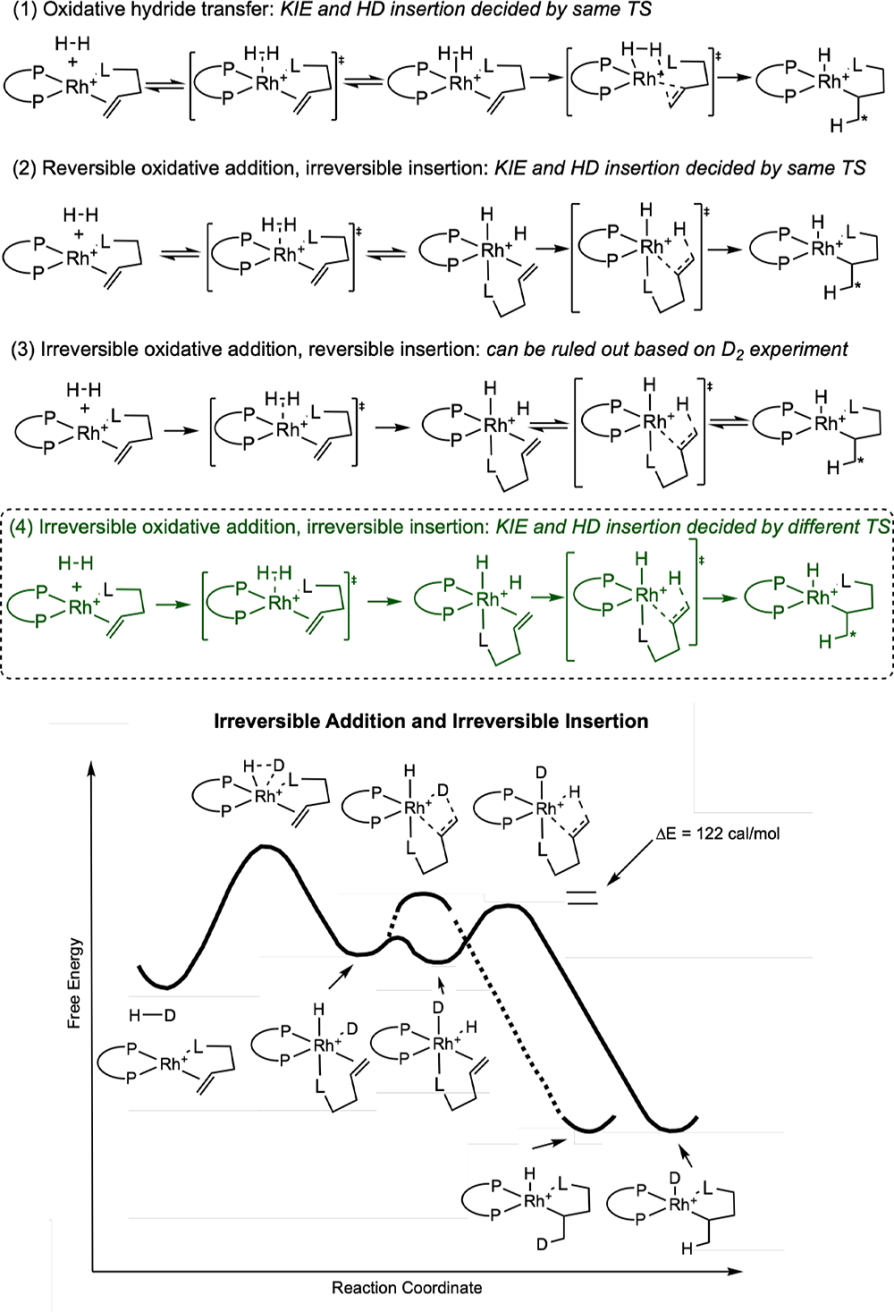
Potential Pathways for Reaction of Rhodium–Enamide Complexes with H_2_ and Explanation of HD Partitioning by an Irreversible Oxidative Addition, Irreversible Insertion Pathway (4) (Landis, 1998 ^29^)­^a^

a Adapted with permission from ref 29. Copyright 1998 Elsevier.

The first and second possibilities were ruled out from the observation of an HD partitioning ratio that is larger than the H_2_/D_2_ KIE. In either the case of oxidative hydride transfer or reversible oxidative addition followed by irreversible migratory insertion, the overall reaction should be controlled by a single irreversible transition state. Because they are reporting on the same transition state, the HD partitioning ratio should be between 1.00 and the value of the KIE (1.04). Because the HD partitioning ratio is significantly larger than the H_2_/D_2_ KIE, it must be reporting on different transition states, and therefore neither (1) nor (2) can be the operative pathway. Pathway (3) is ruled out from the observation that the catalytic reaction with D_2_ does not lead to HD scrambling in the product, meaning that migratory insertion cannot be reversible. Therefore, only path (4), irreversible oxidative addition followed by irreversible migratory insertion, is feasible. Here, the H_2_/D_2_ KIE reports on the relative rates of oxidative addition, whereas the HD partitioning ratio reports on the relative rates of migratory insertion into the Rh–D and Rh–H bonds, where insertion into the Rh–H bond is faster (Scheme 8 bottom).

To understand why the reaction of H_2_ occurred at such a faster rate with the minor diastereomer, Landis and co-workers performed a computational study in 2001 using ^Me^DuPhos as the representative ligand and a variety of substrates bearing different α-substituents.^30^ This work aimed to rationalize a (then) recent finding by Burk that hydrogenation of an enamide containing a *tert*-butyl α-substituent resulted in a reversal of absolute stereochemistry.^31^ Assuming that the reaction follows the unsaturated pathway presented in Scheme 4, the relative energies of the major and minor rhodium–substrate diastereomers were controlled by the electronic nature of the α-substituent on the substrate. Electron-withdrawing substrates led to a strong preference for the α-carbon to lie in the square plane, therefore forcing the β-carbon out of the plane (Scheme 9). Steric interactions between the β-carbon and the methyl groups of the ligand control which enantioface coordinates more tightly. In the minor diastereomer, a distortion is required to remove steric constraint, raising the energy of this diastereomer and increasing its reactivity toward H_2_. In addition, oxidative addition of H_2_ to the major rhodium–substrate diastereomer is disfavored because it involves motion of the α-substituent across the hindered quadrant. These computational results rationalize the experimental observations that the minor diastereomer in solution leads to the major product in very high *ee*. Reversal of this effect with bulky, electron-donating α-substituents helps explain the previously observed enantio-reversal with these substrates and is useful for understanding new catalysts.

**Scheme 9:**
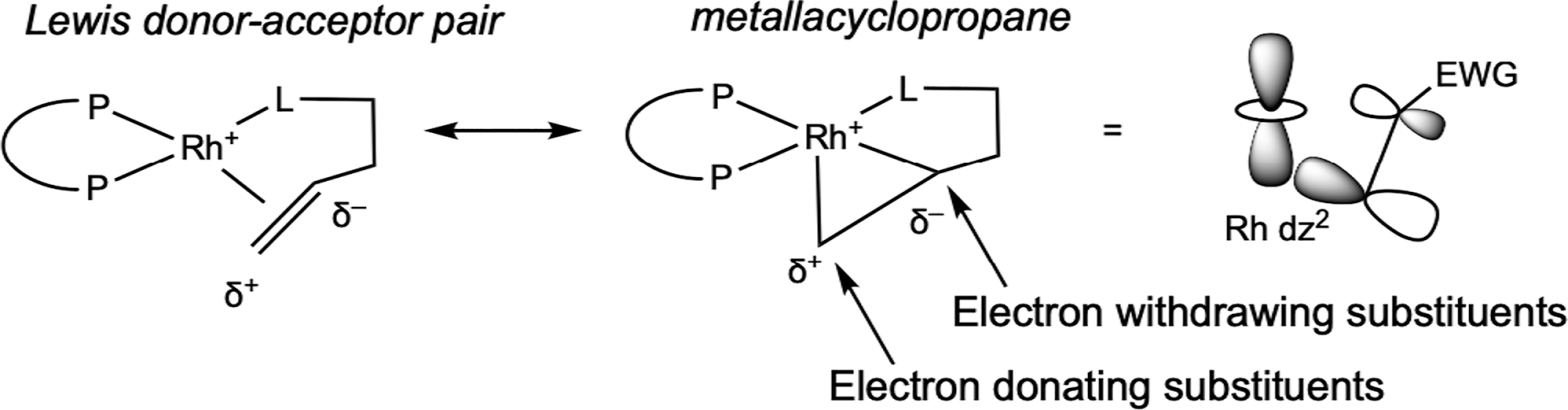
Simplified Orbital Interaction between Filled d_*z* ^2^_ Orbital of the Rhodium Center and C=C π* Anti-bonding Orbital of the Alkene, Where Electron-Withdrawing Groups on the α-Carbon Influence the β-Carbon to Move Axially Closer to Rh (Landis, 2001 ^30^)­^a^

a Adapted from ref 30. Copyright 2001 American Chemical Society.

Overall, the combined experimental and computational studies have painted a relatively comprehensive picture of the unsaturated pathway for α,β-dehydroamino acid and enamide hydrogenation with cationic bis­(phosphine) rhodium­(I) complexes (Scheme 10).^22^ In a standard case following an “anti-lock-and-key” unsaturated pathway, reaction of [bis­(phosphine)­Rh­(S)_2_]^+^ with a chelating substrate reversibly leads to the formation of two rhodium–substrate complexes. In most reported examples, the major rhodium–substrate diastereomer in solution does not lead to the major product. Instead, a significantly faster rate of reaction for the minor rhodium–substrate diastereomer with H_2_ through irreversible oxidative addition generates the rhodium dihydride substrate intermediate in the rate- and enantio-determining step. This complex undergoes rapid, irreversible migratory insertion to generate a rhodium monohydride alkyl. Finally, reductive elimination, followed by coordination of either solvent or a new molecule of substrate releases the hydrogenated product and restarts the catalytic cycle. While this is what is often presented in many organometallic chemistry classes and textbooks, this is not where the story ends.

**Scheme 10:**
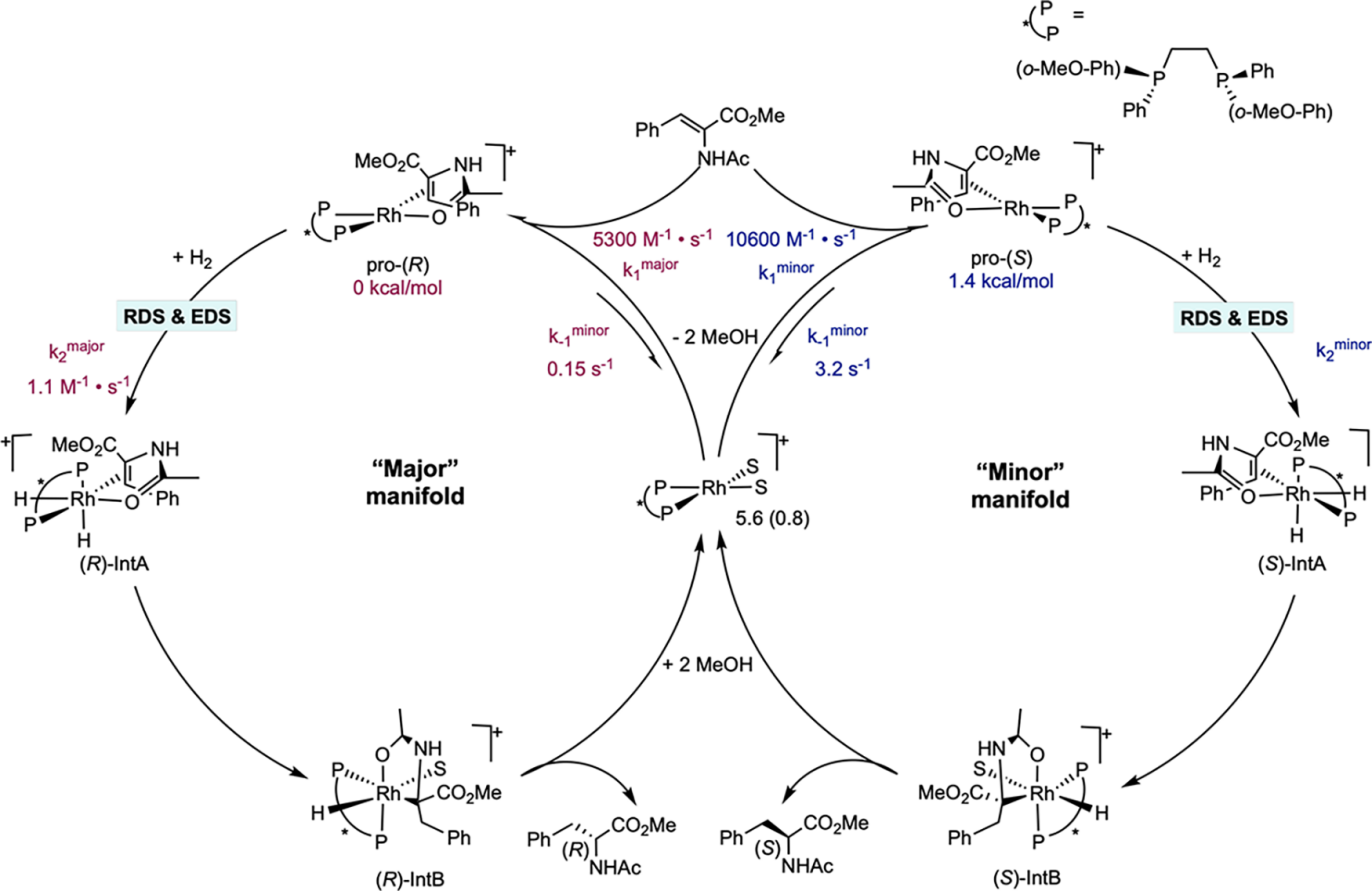
“Anti-Lock-and-Key” Unsaturated Mechanism for Enamide Hydrogenation by Bis­(phosphine) Rhodium­(I) Cationic Complexes (Halpern, 1987 ^22^)­^a^

a Adapted from ref 22. Copyright 1987 American Chemical Society.

### Evidence for Dihydride Pathways

Following the comprehensive studies of Halpern and Brown, many considered the mechanism of rhodium-catalyzed asymmetric olefin hydrogenation to be fully understood. However, in the early 2000s Gridnev and Imamoto reported that when the bis­(phosphine) ligand is changed from an electron-poor ligand, such as DIPAMP, to a more electron-donating variant, such as BisP* (BisP* = (*S*,*S*)-1,2-bis­(*tert*-butylmethylphosphino)­ethane), a dihydride pathway for rhodium-catalyzed asymmetric enamide hydrogenation may become operative. The major theme in this work is that the relative rate of H_2_ oxidative addition is accelerated such that it is kinetically preferred over coordination of the unsaturated substrate.

The initial step in the dihydride pathway is the reaction of a [bis­(phosphine)­Rh­(S)_2_]^+^ with dihydrogen, first to form a σ-bound intermediate, followed by H–H cleavage to form a [bis­(phosphine)­Rh­(H)_2_(S)_2_]^+^ complex. In 2000, Gridnev and Imamoto demonstrated that when hydrogen gas was mixed with a methanol solution of [(BisP*)­Rh­(MeOH)_2_]­[BF_4_] at −90 °C, two diastereomeric dihydride complexes of [(BisP*)­Rh­(H)_2_(MeOH)_2_]­[BF_4_] were observed in a 10:1 ratio by NMR spectroscopy (Scheme 11).^32^ Warming of this reaction mixture to room temperature resulted in loss of dihydrogen from the rhodium complex to regenerate rhodium­(I). This work demonstrated that rhodium­(III) dihydrides are observable, albeit at low temperature and in the presence of H_2_. This attribute of complexes bearing more electron-rich phosphine ligands logically follows from their ability to generate a more reducing metal center, and hence they are more prone to form stable structures in higher oxidation states.

**Scheme 11:**
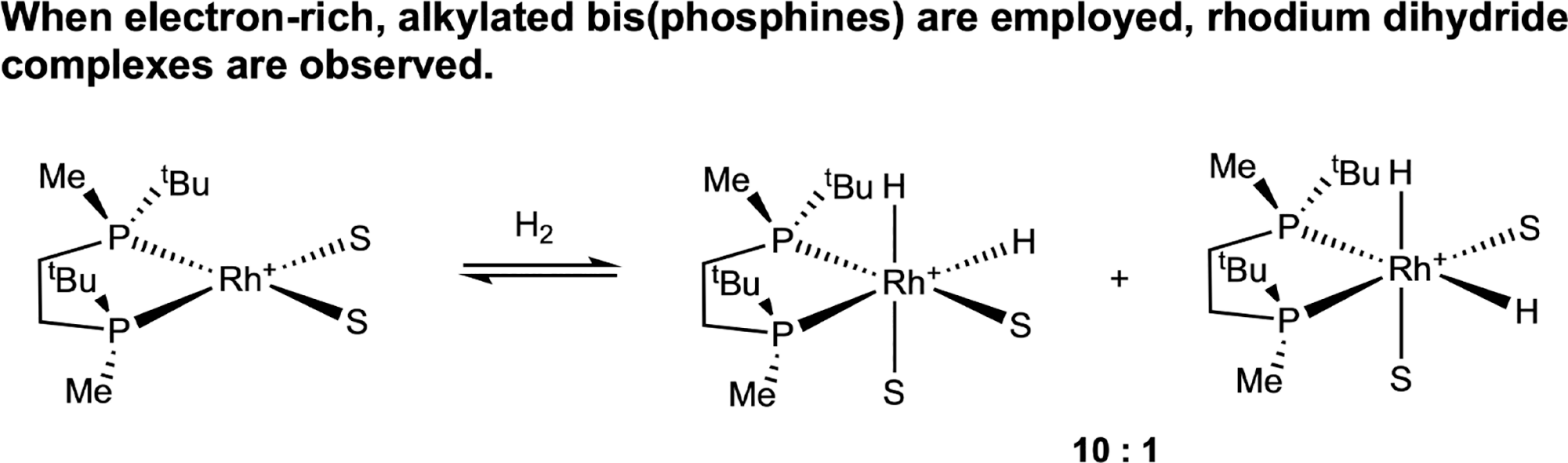
Formation of Rhodium­(III) Dihydride Complexes from Oxidative Addition of H_2_ (Imamoto, 2000 ^32^)­^a^

a Adapted from ref 32. Copyright 2000 American Chemical Society.

In a subsequent example using a 7-membered ring chelating bis­(phosphine), BixP* (BixP* = (*S*,*S*)-α,α′-bis­(*tert*-butyl­methyl­phosphino)-*o*-xylene), mixing of hydrogen gas with a methanol solution of [(BixP*)­Rh­(NBD)]­[BF_4_] at −70 °C produced diastereomeric [(BixP*)­Rh­(H)_2_(MeOH)_2_]­[BF_4_] complexes.^33^ Warming of this reaction mixture to room temperature generated a new, stable bridging tetrahydride dimer, [(BixP*)­Rh­(H)­(MeOH)]_2_(μ_2_-H)_2_[BF_4_]_2_ (Figure 4), as determined by NMR spectroscopy. This report demonstrated that the formation of rhodium dihydride complexes with electron-rich bidentate phosphines is not isolated to a single example and that in the appropriate coordination environment these dihydride complexes persist at room temperature.

**Figure 4:**
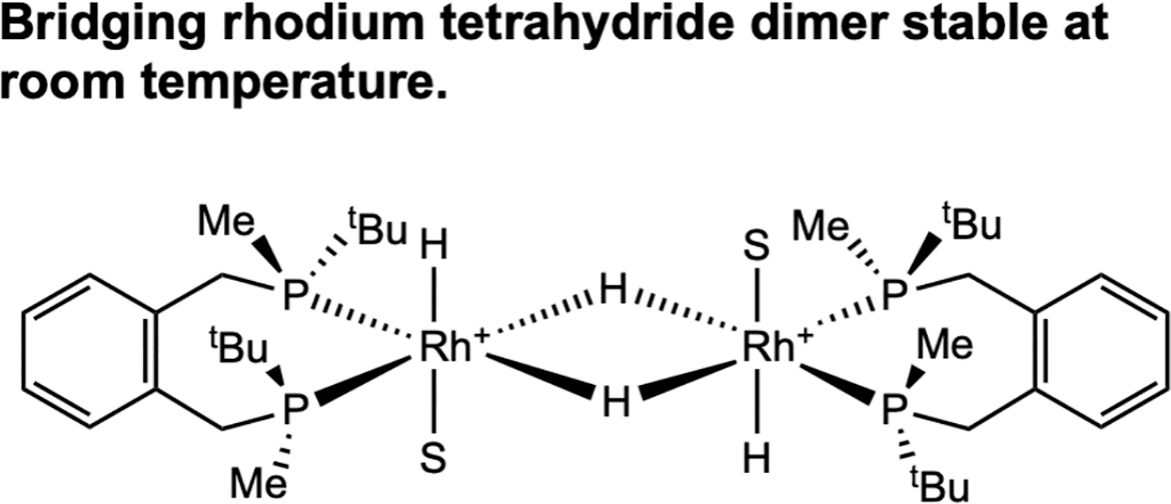
Bridging tetrahydride dimer, [(BixP*)­Rh­(H)­(MeOH)]_2_(μ_2_-H)_2_[BF_4_]_2_, observed by NMR spectroscopy at room temperature (Imamoto, 2001 ^33^). Adapted from ref 33. Copyright 2001 American Chemical Society.

In the dihydride mechanism, the rhodium dihydride bis­(solvento) complex reacts with an equivalent of substrate to form the catalyst–substrate complex that undergoes migratory insertion to yield the rhodium­(III) monohydride alkyl complex. Addition of one equivalent of MAC to [(BisP*)­Rh­(H)_2_(MeOH)_2_]­[BF_4_] at low temperature enabled observation of the rhodium monohydride alkyl (Scheme 12).^32^ As with the examples reported by Halpern and Brown, this supports formation of a highly reactive rhodium­(III) dihydride substrate intermediate. Subsequent reductive elimination from the rhodium monohydride alkyl released the hydrogenated product in high ee, reproducing the observed catalytic enantio­selectivity. Repeating this reaction sequence using HD gas generated a partitioning ratio of 1.18:1.00 for the α-deuterated product, whereas *catalytic* hydrogenation with HD produced a 1.17:1.00 ratio, suggesting that the dihydride pathway may be operative. Likewise, when one equivalent of MAC was added to [(BixP*)­Rh­(H)_2_(MeOH)_2_]­[BF_4_] at low temperature, the formation of a rhodium monohydride alkyl was observed as the product of migratory insertion and subsequent reductive elimination generated the hydrogenated product.^33^

**Scheme 12:**
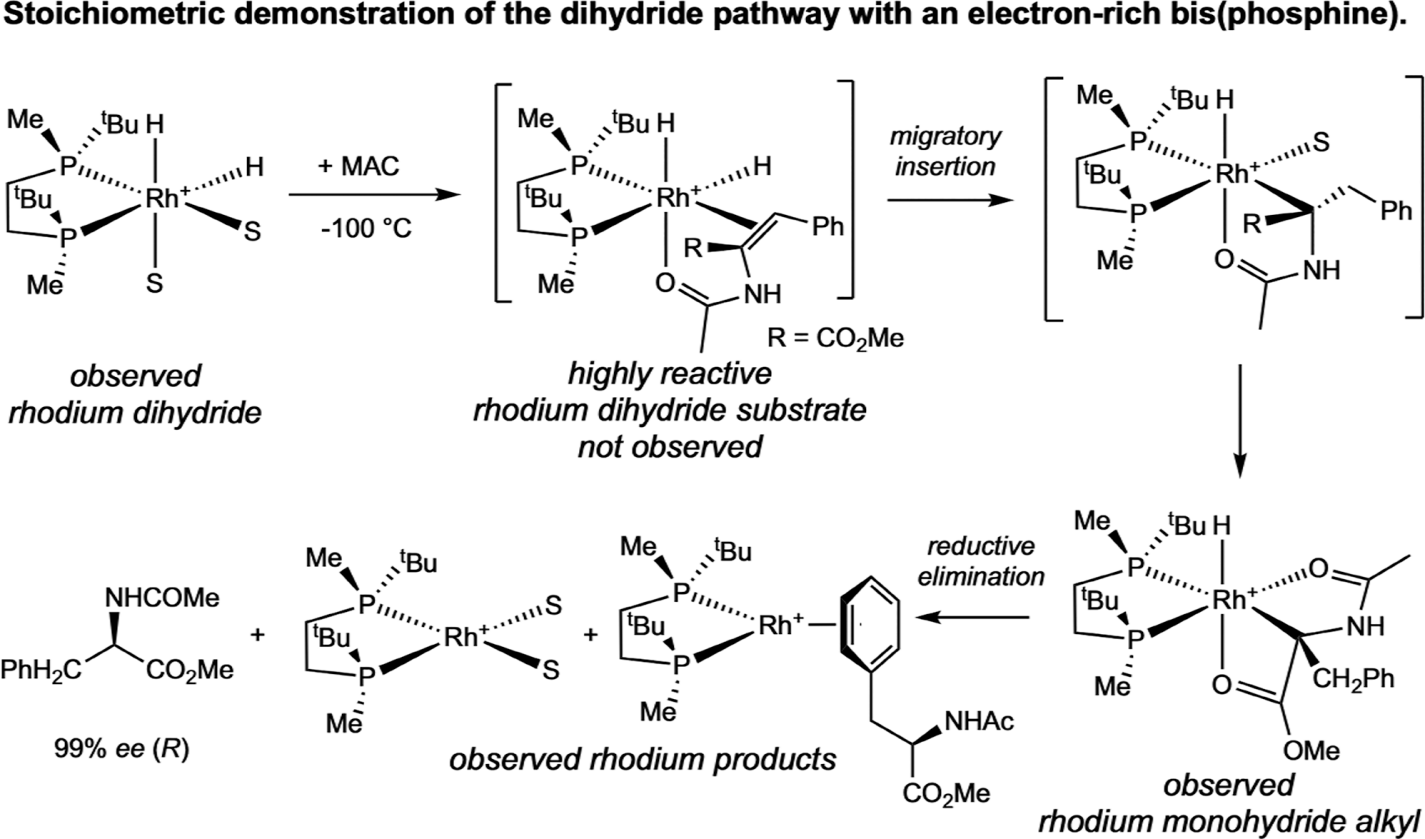
Addition of Enamide to [(BisP*)­Rh­(H)_2_(MeOH)_2_]­[BF_4_] Gives a Rhodium Monohydride Alkyl Observable at Low Temperatures (Imamoto, 2000 ^32^)­^a^

a Adapted from ref 32. Copyright 2000 American Chemical Society.

Use of a tethered BisP*-type tetra­(phosphine) ligand to form a dinuclear rhodium complex allowed the direct observation of a dirhodium tetrahydride substrate complex through reaction of a tethered dirhodium tetrahydride complex with MAC (Scheme 13).^34^ In the observed structure, MAC is coordinated to one of the rhodium centers through only the oxygen of the amide carbonyl, with the C=C bond dissociated. Subsequent olefin coordination and migratory insertion generated the observable dirhodium trihydride alkyl, followed by reductive elimination in the presence of excess MAC to release the hydrogenated product. This work supports that rhodium dihydride substrate complexes, where the C=C bond is dissociated from the metal center, may be relevant to catalysis.

**Scheme 13:**
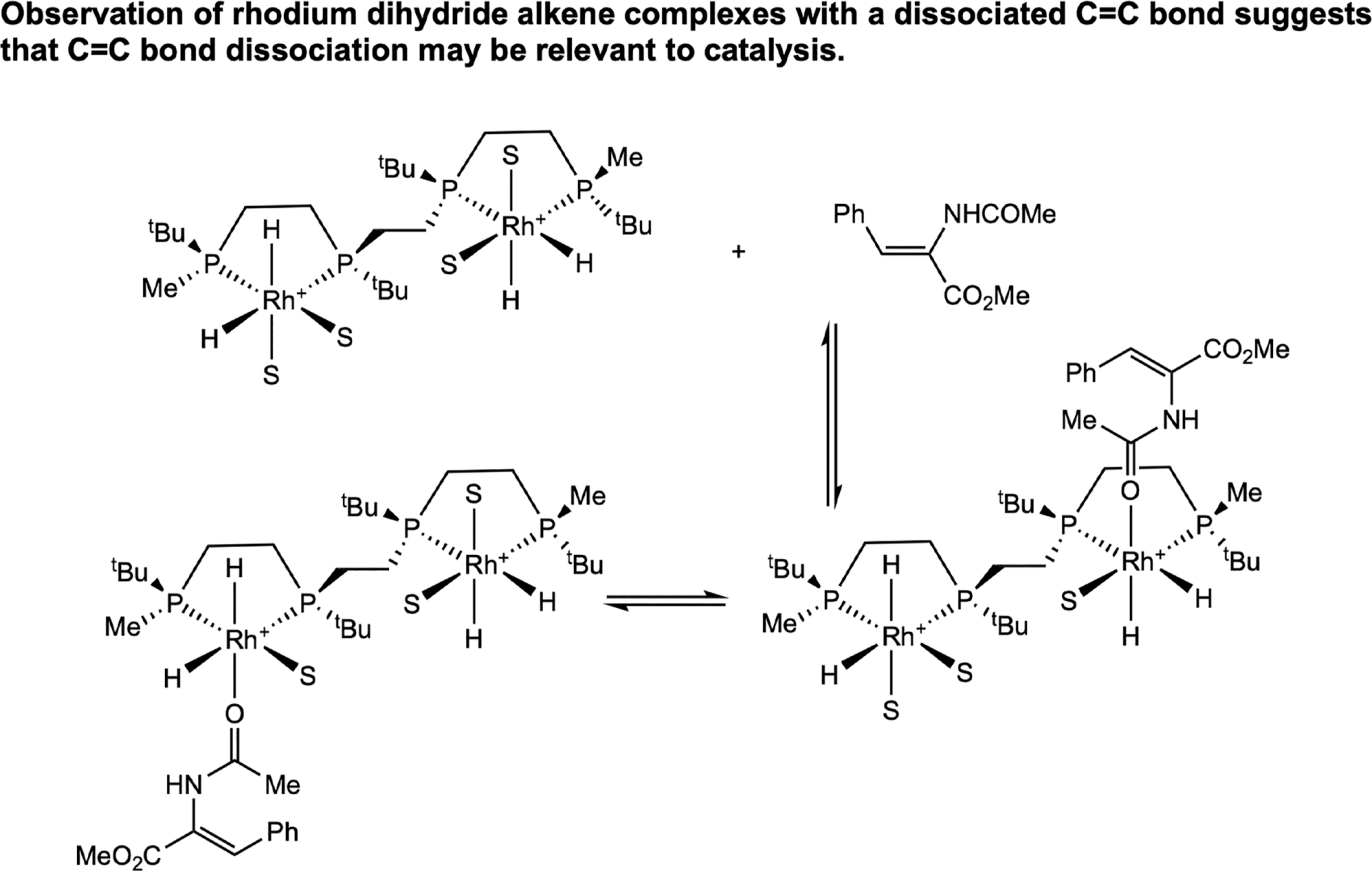
Observation of a Dihydride Substrate Complex with a Dissociated C=C Bond Using a Tethered BisP* Ligand (Imamoto, 2001 ^34^)­^a^

a Adapted from ref 34. Copyright 2001 American Chemical Society.

It should be noted that in these examples, the unsaturated pathway was not convincingly eliminated. Simulations established that at low temperature, unsaturated pathways with electron-rich bis­(phosphine) ligands were energetically feasible. Formation of [(BisP*)­Rh­(MAC)]­[BF_4_] complexes, followed by low-temperature reaction with H_2_, did generate the hydrogenated product, although with slightly lower ee and at a slower rate than the corresponding simulated dihydride pathway.^32^ However, because these reactions were performed at low temperature, it is difficult to rule out the catalytic relevance of an unsaturated pathway based on this evidence alone. In addition, Gridnev and Imamoto do not provide catalytic kinetic data, making it difficult to determine which pathway is responsible for productive turnover.

Importantly, one of the primary contributions of this work was to demonstrate that simulation of a dihydride pathway reproduces the observed catalytic enantio­selectivity. Computational studies reported by Gridnev and Imamoto in 2008 offer a plausible explanation for the origin of enantio­selection in the dihydride pathway.^35^ Models with both BisP* and MiniPhos (MiniPhos = (*R,R*)-1,2-bis­(*tert*-butylmethylphosphino)­methane) ligands established a low-energy isomerization between dihydride substrate chelate complexes through C=C dissociation prior to migratory insertion. Use of explicit solvent molecules in the computations allowed for modeling of an energetically plausible pathway for this isomerization, through methanol assistance with double bond dissociation. The authors proposed that preferred placement of the substrate chelate in the less hindered quadrant of the rhodium dihydride substrate complex is the source of eventual enantio­selectivity, which is locked in during a subsequent irreversible migratory insertion step.^36^

This model challenges the commonly held view that the enantio­selectivity-determining step in rhodium-catalyzed asymmetric hydrogenation derives exclusively from the unsaturated pathway and the relative barrier heights derived from the oxidative addition of H_2_. Both the dihydride and unsaturated pathways were reconciled through isomerization at the rhodium dihydride substrate chelate intermediate, the first converging intermediate between the two pathways (Scheme 14).^37^ Through this explanation of enantio­selection provided by Gridnev and Imamoto, both pathways could produce highly enantio­selective hydrogenation despite the divergence in the initial stages. An alternative explanation is that the much faster rate of oxidative addition to the minor rhodium substrate complex along the unsaturated pathway provides the same result, forming the rhodium dihydride substrate complex with the substrate chelate in the less hindered quadrant without the need for further isomerization.

**Scheme 14:**
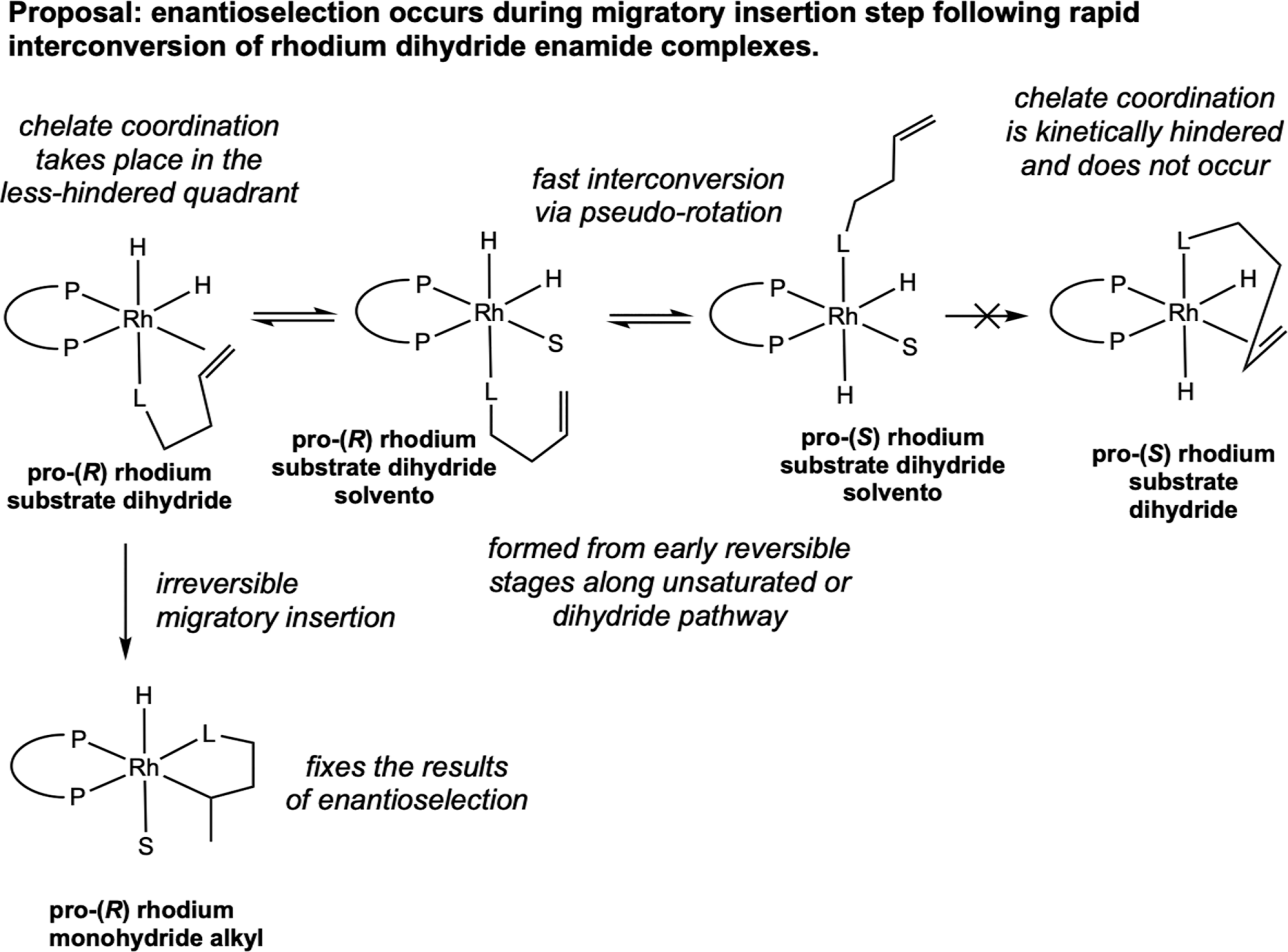
A Unified Mechanistic Proposal for Enantioselection in Rhodium-Catalyzed Asymmetric Hydrogenation (Imamoto, 2004 ^37^)­^a^

a Adapted from ref 37. Copyright 2004 American Chemical Society.

In their efforts to explain the remarkable enantio­selectivities obtained with cationic rhodium hydrogenation catalysts bearing both electron-rich and electron-poor bis­(phosphine) ligands, Gridnev and Imamoto offered a new quadrant model for predicting the hand of enantio­selectivity, updating the original quadrant model proposed by Knowles in 1983 ^38^ (Figure 5). This model accounted for all known examples of rhodium-catalyzed asymmetric hydrogenation, whether the bis­(phosphine) ligand possessed backbone chirality or P-stereogenicity. Sterically demanding substituents on the phosphorus atoms in the top-left and bottom-right quadrants furnished the *R*-hydrogenation products, while large substituents in the top-right and bottom left quadrants give *S*-hydrogenation products. When the substituents in all four quadrants are the same, the quasi-axially positioned groups can be considered as the large groups. According to this explanation, this steric hindrance primarily comes into play during the formation of the substrate chelate of the rhodium dihydride substrate complex prior to enantio-determining migratory insertion.

**Figure 5:**
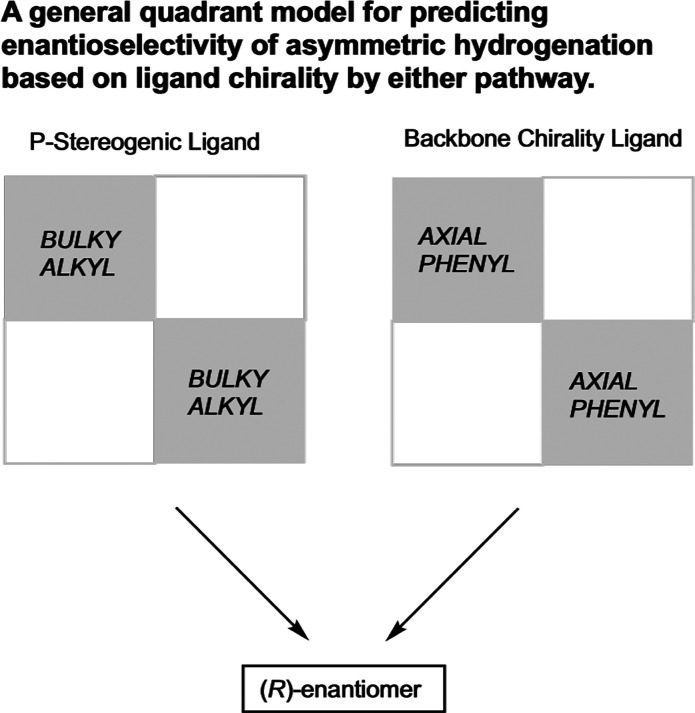
Quadrant model for rhodium-catalyzed asymmetric hydrogenation of enamides (Imamoto, 2004 ^37^). Adapted from ref 37. Copyright 2004 American Chemical Society.

### Summary of the Mechanistic Pathways for Rhodium-Catalyzed Asymmetric Hydrogenation

In the preceding section, both the unsaturated and dihydride pathways were presented as limiting mechanistic frameworks to understand and account for the highly enantio­selective hydrogenation of alkenes promoted by cationic bis­(phosphine) rhodium catalysts. What emerges is a nuanced mechanistic picture that depends on the electronic properties of the bis­(phosphine) supporting ligand. These pathways primarily differ in the initial stages of the catalytic cycle, influenced by a preference for either substrate coordination or oxidative addition of hydrogen to the rhodium catalyst. Both pathways have been thoroughly studied both experimentally and computationally and should help guide future research on rhodium-catalyzed asymmetric hydrogenation.

In the unsaturated pathway proposed by Halpern, Landis, and Brown, the rate- and enantio-determining step is the oxidative addition of hydrogen to the reversibly formed rhodium substrate complex and occurs with relatively electron-poor phosphine ligands. The enantio­selectivity of this reaction proceeds from the much faster reaction of the “right”/minor diastereomer of the rhodium substrate complex with H_2_ as compared to the “wrong”/major diastereomer. Hence, the initial ratio of diastereomer formation is not the origin of eventual enantio­selectivity.

In the dihydride pathway proposed by Gridnev and Imamoto, substrate coordination occurs following formation of a rhodium dihydride bis­(solvento) complex and is operative with complexes bearing more electron-rich phosphines. In this mechanism, reversible chelation of the C=C bond occurs to place the substrate chelate in the less hindered quadrant of the rhodium complex, followed by rate- and enantio-determining migratory insertion. While no kinetic data has been provided to establish the overall preference for this pathway and relevance to catalysis, the authors do provide compelling stoichiometric evidence that a dihydride pathway can be simulated to generate highly enantio­enriched products with the same absolute sense of stereochemistry as the catalytic reaction.

To reconcile these two pathways, Gridnev and Imamoto suggest that reversible C=C bond dissociation, followed by enantio-determining migratory insertion is the source of the remarkable enantio­selectivity observed for catalysts bearing both electron-rich and electron-poor bis­(phosphine) ligands. Along with this explanation, the authors offered a new quadrant model that consolidates all known examples with chelating substrates, independent of the nature of the bis­(phosphine) ligand. One important takeaway from these studies is that thorough experimental efforts including kinetics and determination of isotope effects should be combined with computational studies to provide support for or refute particular mechanistic scenarios.

In the years following Knowles’s initial discovery, the scope of rhodium-catalyzed asymmetric hydrogenation has been expanded to include a wide variety of substrates, ligands and new catalysts. As asymmetric reactions are discovered, mechanistic possibilities other than the limiting pathways described in the previous sections for α,β-unsaturated dehydroamino acid derivatives are possible. One notable example is the asymmetric hydrogenation of 2-pyridyl-substituted alkenes, the mechanism of which was thoroughly studied by Blackmond and co-workers. Though an unsaturated-type pathway is proposed to lead to productive catalysis, persistent rhodium hydrides are observed as off-cycle intermediates.^39^ Another notable example is the asymmetric hydrogenation of 3-amino-4-alkyl/aryl-disubstituted maleimides, published by Zhang and co-workers, where a pseudo dihydride-type all-Rh­(III) cycle involving two different molecules of hydrogen in the product forming step is proposed.^40^ These examples highlight that though the study of rhodium-catalyzed asymmetric hydrogenation has been extensive, there is still much to learn and discover.

## Iridium

### Brief Overview of Iridium-Catalyzed Asymmetric Olefin Hydrogenation

While the catalytic alkene hydrogenation activity of iridium complexes had long been known, synthetically useful asymmetric variants lagged behind the more widely used rhodium examples for decades. Pioneering examples of iridium catalysts for asymmetric alkene hydrogenation were reported by Pfaltz and co-workers in 1998.^41^ Taking inspiration from Crabtree’s achiral iridium hydrogenation catalyst, which bears both single monodentate phosphine and pyridine ligands, Pfaltz developed chiral bidentate variants known as phosphine–oxazoline (PHOX) ligands. These ligands derive their chirality from the sterically demanding α-substituent on the oxazoline moiety and have proven useful in asymmetric hydrogenation of minimally functionalized olefins. A breakthrough example was the asymmetric hydrogenation of α-methyl stilbene employing a [(PHOX)­Ir­(COD)]­[BAr^F^_4_] (BAr^F^_4_ = tetrakis­[3,5-bis­(trifluoromethyl)­phenyl]­borate) as the precatalyst, which generated the desired alkane in quantitative conversion and 98% *ee* (Scheme 15). The identity of the anion proved important to the stability of these catalysts, as analogues bearing either [PF_6_]^−^ or [BF_4_]^−^ counterions were susceptible to decomposition.

**Scheme 15:**
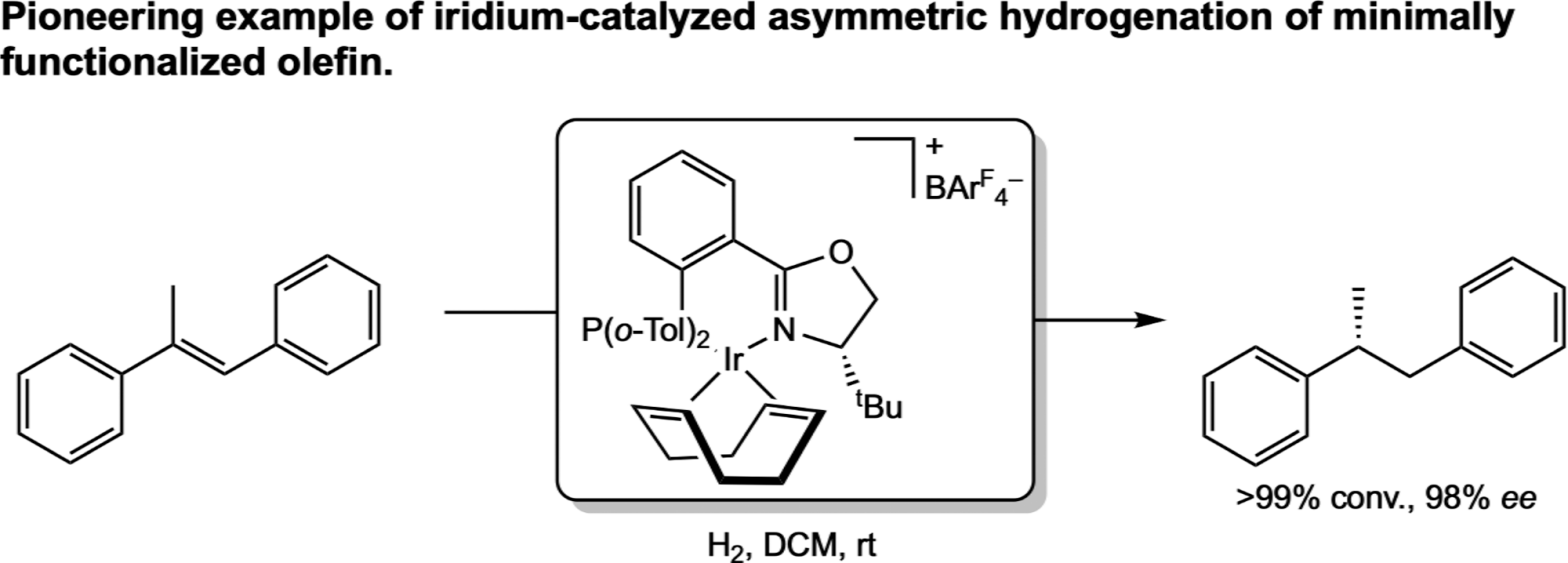
(PHOX)­Ir-Catalyzed Hydrogenation of α-Methyl Stilbene (Pfaltz, 1998 ^41^)

Since this initial work, iridium complexes bearing chiral bidentate P,N- or C,N- ligands have been applied to the asymmetric hydrogenation of a variety of substrates, including minimally functionalized olefins, furans, alcohols, esters, carboxylic acids, and phosphates (Scheme 16).^5^ Chiral iridium catalysts have also found widespread application in the asymmetric hydrogenation of imines, ketones, and *N*-heterocycles, though these examples and their mechanisms of action are outside of the scope of this Tutorial. Due to the lack of functional groups on the alkene substrates, iridium catalysis is typically conducted in non-coordinating solvents as those than can function as ligands often suppress turnover. Even when using poorly coordinating solvents, such as dichloromethane, the solvent may bind to vacant coordination sites on the strongly Lewis acidic iridium center. Such solvents are often used during the hydrogenation of minimally functionalized olefins.

**Scheme 16:**
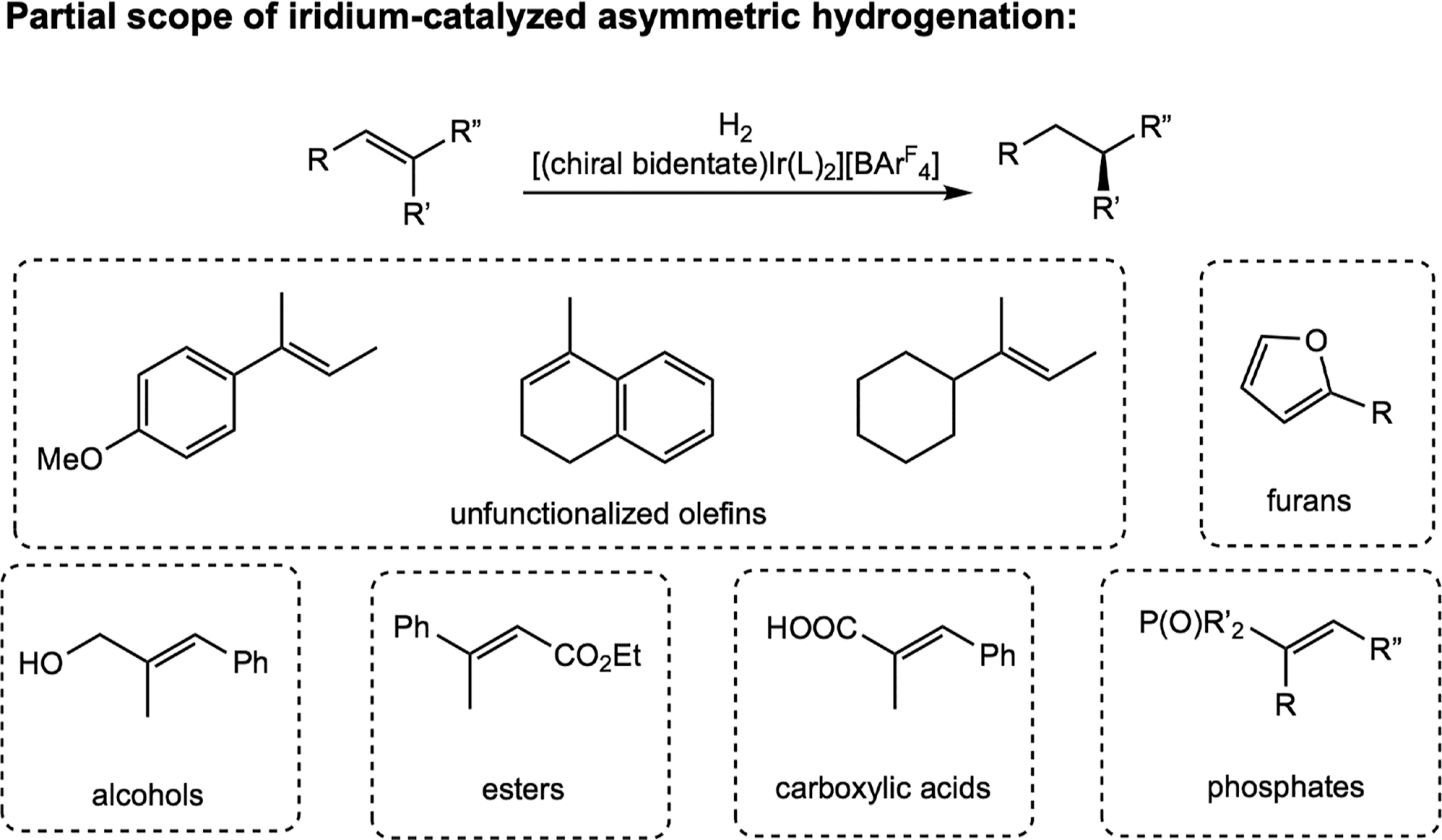
General Scheme for Iridium-Catalyzed Asymmetric Hydrogenation with Examples of Substrate Classes That Undergo Highly Enantioselective Hydrogenation^5^

The mechanism of iridium-catalyzed asymmetric hydrogenation has been thoroughly investigated through a combination of experimental and computational studies. Distinct from rhodium, iridium can not only access the +1 and +3 oxidation states but also the +5, expanding the range of possible pathways. Due to the unique performance, mechanistic studies in iridium-catalyzed asymmetric hydrogenation have principally focused on minimally functionalized alkenes although select examples with coordinating functional groups have been reported and will be discussed at the end of this section.

### Mechanistic Proposals for Iridium-Catalyzed Asymmetric Hydrogenation of Minimally Functionalized Alkenes

Both Ir­(I/III) and Ir­(III/V) cycles have been considered for the mechanism of iridium-catalyzed asymmetric hydrogenation of minimally functionalized olefins. The proposed iridium­(I/III) cycle is similar to the mechanism of rhodium-catalyzed asymmetric hydrogenation, though without substrate chelation (Scheme 17A). Substrate coordination and H_2_ oxidative addition lead to the formation of a key Ir­(III) dihydride alkene complex, which undergoes migratory insertion to form an Ir­(III) monohydride alkyl, followed by reductive elimination and ligand exchange to furnish an Ir­(I) intermediate and the hydrogenated product.

**Scheme 17:**
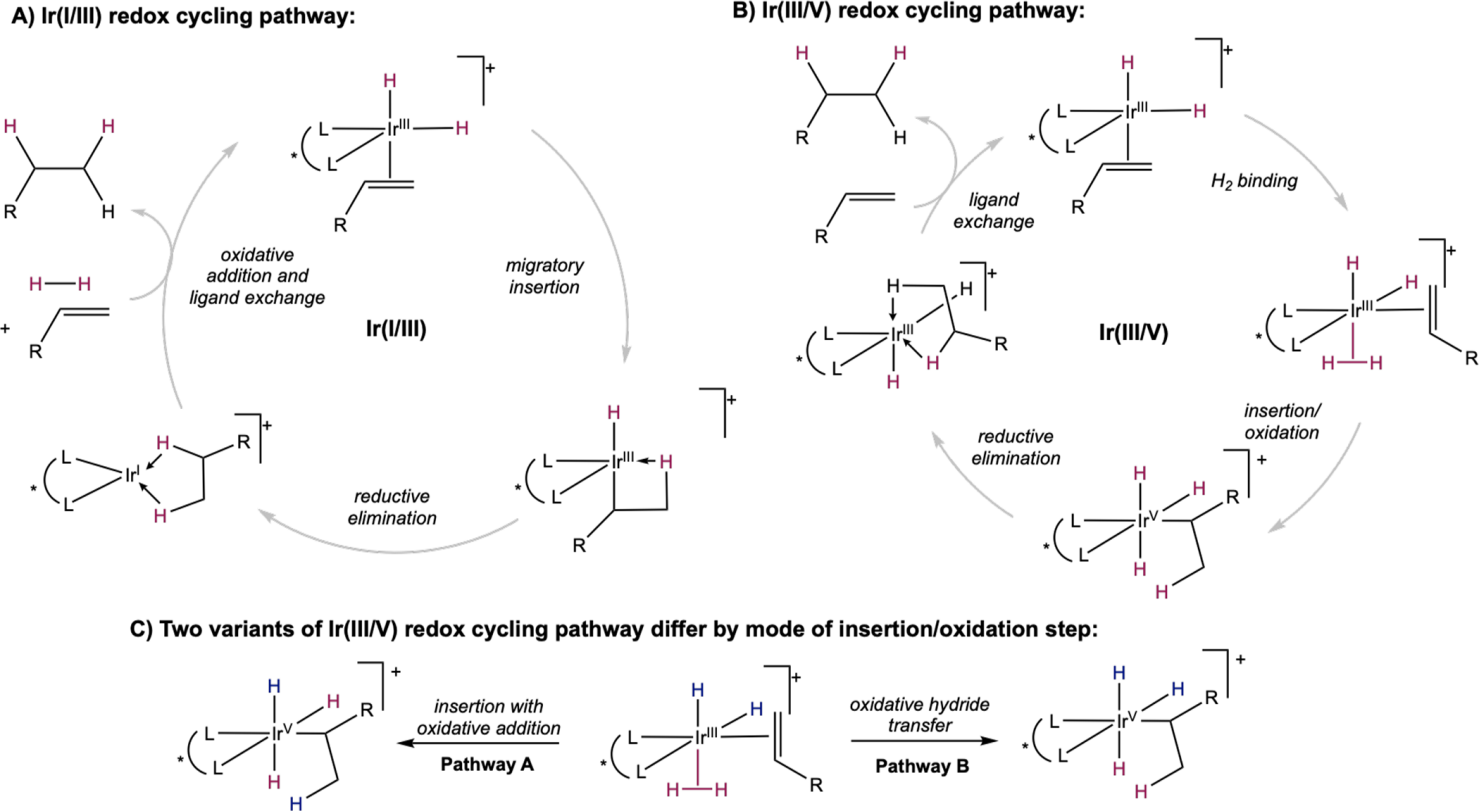
Proposed Pathways for Iridium-Catalyzed Asymmetric Hydrogenation of Minimally Functionalized Olefins

In the Ir­(III/V) cycle, it is proposed that migratory insertion does not occur directly from the Ir­(III) dihydride substrate complex, but rather that coordination of an additional dihydrogen molecule is required to induce insertion (Scheme 17B). Once the key Ir­(III) dihydride dihydrogen alkene complex is formed, insertion occurs concomitantly with oxidative addition to form an Ir­(V) trihydride alkyl. This insertion/oxidation step may occur in one of two ways (Scheme 17C). One possibility is that insertion of the alkene into an Ir–H bond occurs in concert with oxidative addition of the bound σ-H_2_ (pathway A). The other possibility is that alkene insertion occurs directly into the σ-bound H_2_ by oxidative hydride transfer (pathway B). Regardless of the pathway of insertion/oxidation, an Ir­(V) trihydride alkyl is formed, which can then undergo reductive elimination and ligand exchange to furnish an Ir­(III) dihydride complex and the hydrogenated product.

### Mechanistic Results in Iridium-Catalyzed Hydrogenation of Minimally Functionalized Alkenes

Kinetic experiments performed by Andersson and co-workers in 2003 using [(PHOX)­Ir­(COD)]­[BAr^F^_4_] for the hydrogenation of (*E*)-methyl-*trans*-stilbene established a first-order dependence on H_2_ pressure and catalyst along with a zeroth-order dependence on substrate, supporting rate-determining addition of H_2_ to the iridium catalyst.^42^ Similarly, Burgess and co-workers reported in 2005 that in the hydrogenation of dienes using an *N*-heterocyclic carbene–oxazole (C,N) ligand, that the kinetic data showed a first-order dependence on catalyst and hydrogen and a zeroth-order dependence on diene.^43^ Taken alone, these results could either support an “unsaturated”-type Ir­(I/III) cycle, with rate-limiting oxidative addition of H_2_, or an Ir­(III/V) cycle, where reaction with a second molecule of dihydrogen is rate-limiting.

A key intermediate in both the Ir­(I/III) or Ir­(III/V) pathways is the Ir­(III) dihydride alkene complex. In 2004, Pfaltz and co-workers found by NMR spectroscopy that addition of H_2_ to a THF-*d*_*8*_ solution of [(PHOX)­Ir­(COD)]­[BAr^F^_4_] at −40 °C generated a new iridium dihydride diene complex, [(PHOX)­Ir­(H)_2_(COD)]­[BAr^F^_4_] (Scheme 18A).^44^ Only one of the four possible diastereomers of this intermediate iridium complex was observed and is derived from the addition of H_2_ to the more sterically encumbered face of the starting complex. The resulting Ir–H bond is formed *trans* to the nitrogen of the PHOX ligand, an arrangement also observed with Crabtree’s catalyst. In 2014, Pfaltz and co-workers performed a similar study using both C,N (C,N = *N*-heterocyclic carbene/oxazoline) and P,N-type (PHOX) ligands.^45^ Addition of excess dihydrogen and pro-chiral alkene to a solution of [(C,N)­Ir­(COD)]­[BAr^F^_4_] at −35 °C resulted in the formation of a new iridium dihydride alkene complex, [(C,N)­Ir­(alkene)­(H)_2_)]­[BAr^F^_4_], as determined by NMR spectroscopy. Similar results were observed when the same series of experiments was carried out using the PHOX ligand to form [(PHOX)­Ir­(alkene)­(H)_2_)]­[BAr^F^_4_] complex (Scheme 18B). This complex was obtained as a mixture of rapidly equilibrating iridium dihydride alkene major and minor diastereomers. Interestingly, the less stable minor diastereomer was converted to the major product enantiomer. Similarly, in 2015, Diéguez and co-workers reported that addition of both H_2_ and substrate to a [(P,S)­Ir­(COD)]­[BAr^F^_4_] (P,S = phosphine-thioether) precatalyst produced a mixture of two dihydride COD isomers, as well as two dihydride–substrate diastereomers (Scheme 18C).^46^ Both hydrides were identified as *cis* to the phosphorus ligand. Again, the less stable isomer generated the major enantiomer of the product.

**Scheme 18:**
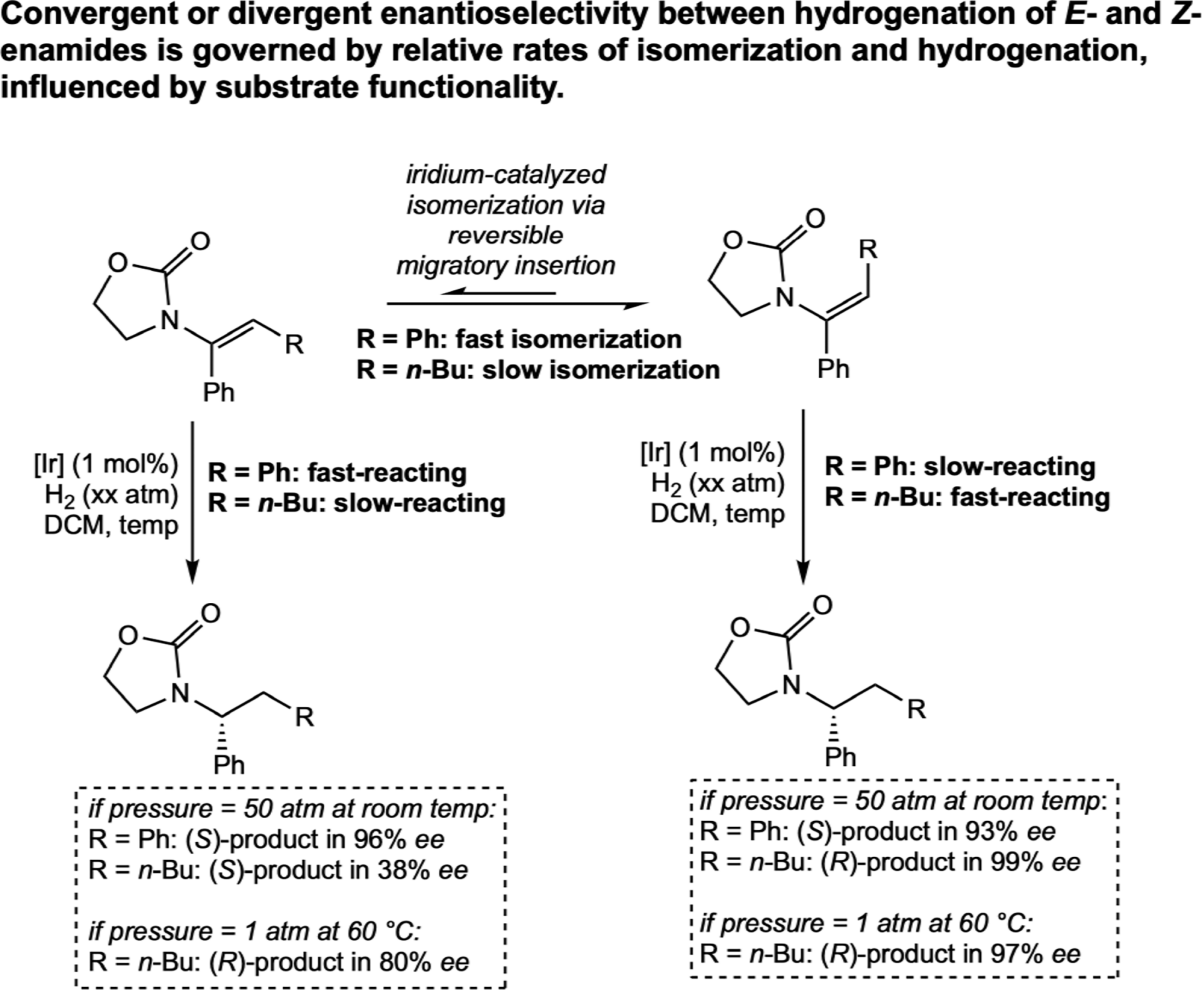
Isolation of Iridium­(III) Dihydride Alkene Complexes (A: Pfaltz, 2004,^44^ B: Pfaltz, 2014,^45^ and C: Diéguez, 2015 ^46^)­^a^

a Adapted from refs 44–46 . Copyrights 2004 American Chemical Society, 2014 John Wiley and Sons, and 2015 American Chemical Society, respectively.

That rapidly interconverting Ir­(III) dihydride alkene complexes were readily observable by low-temperature NMR experiments illustrates a key distinction from the Halpern-type mechanism of rhodium-catalyzed asymmetric hydrogenation. These results suggest that neither alkene coordination nor oxidative addition of dihydrogen is the rate-limiting step in the iridium-catalyzed olefin reduction pathway. In agreement, Pfaltz’s 2004 study found that, while oxidative addition of H_2_ to [(PHOX)­Ir­(COD)]­[BAr^F^_4_] occurred rapidly at low temperatures to form [(PHOX)­Ir­(H)_2_(COD)]­[BAr^F^_4_], subsequent migratory insertion of the diene into the iridium hydride was the slower step.^44^ As the temperature of the solution was raised from −40 to 0 °C, migratory insertion occurred and released cyclooctane and formed an iridium dihydride bis­(solvento) complex, [(PHOX)­Ir­(H)_2_(THF)_2_]­[BAr^F^_4_] (Scheme 19A). Two of the four possible diastereomers of this product were observed in solution, again placing the Ir–H *trans* to the oxazoline nitrogen. Similarly, in the 2014 Pfaltz study, while initial formation of [(C,N)­Ir­(alkene)­(H)_2_)]­[BAr^F^_4_] complexes was fast, subsequent migratory insertion was slower.^45^ In fact, additional H_2_ was required to induce migratory insertion from the iridium dihydride complex (Scheme 19B). These results support an Ir­(III/V) cycle involving a [(C,N)­Ir­(H)_2_(alkene)­(H_2_)­(L)]^+^ intermediate.

**Scheme 19:**
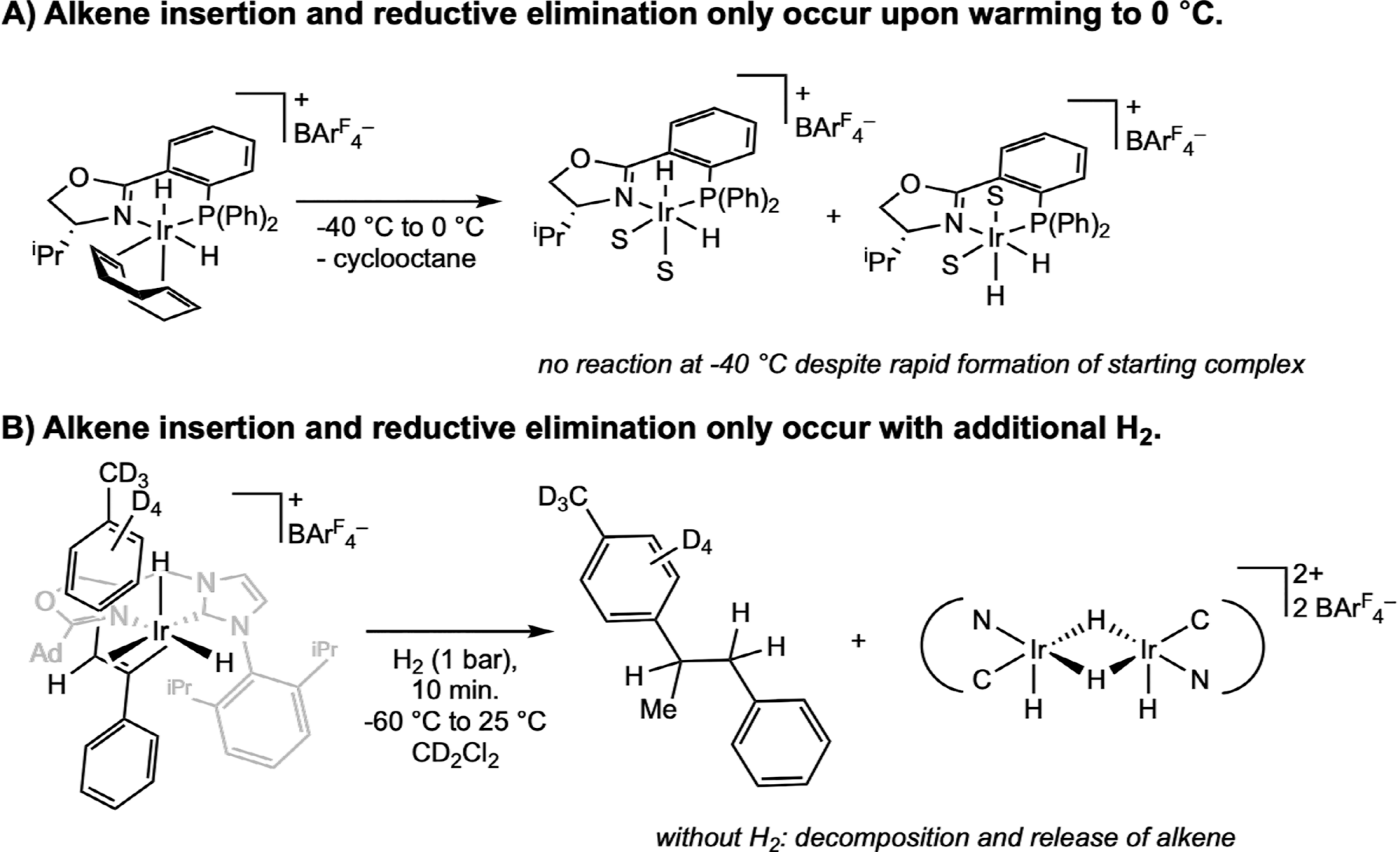
Stoichiometric Experimental Evidence for an Ir­(III/V) Pathway with H_2_-Induced Migratory Insertion as the Rate-Limiting Step (A: Pfaltz 2004,^44^ and B: Pfaltz 2014 ^45^)­^a^

a Adapted from refs 44 and 45 . Copyrights 2004 American Chemical Society and 2014 John Wiley and Sons, respectively.

The only report that contrasts this conclusion comes from Dietiker and co-workers in 2004 using electrospray tandem mass spectrometry to detect iridium-containing reaction intermediates during a gas-phase hydrogenation of styrene.^47^ Efforts to maintain hydrogen pressure throughout the course of the reaction were undertaken and addition of excess styrene and H_2_ to [(PHOX)­Ir­(COD)]­[BAr^F^_4_] generated three new peaks with masses equal to that of [(PHOX)­Ir­(styrene)­(H_2_)_2_]^+^, [(PHOX)­Ir­(styrene)­(H_2_)]^+^, and [(PHOX)­Ir­(styrene)]^+^. Despite the observation of an intermediate with a mass corresponding to either the Ir­(III) dihydride dihydrogen substrate complex or the Ir­(V) trihydride alkyl complex in the Ir­(III/V) catalytic cycle, the authors rule this mechanism out on the basis that preformed [(PHOX)­Ir­(styrene)]^+^ does not react with D_2_ to yield a product with a mass of [(PHOX)­Ir­(styrene)­(D_2_)_2_]^+^ and that there is an absence of trideuterated styrene complexes. On the other hand, the authors found that isolated [(PHOX)­Ir­(ethylbenzene)]^+^ in the absence of hydrogen undergoes dehydrogenation to form [(PHOX)­Ir­(styrene)]^+^. Based on this evidence, the authors concluded that an Ir­(I/III) cycle is operative. However, the lack of solvent in the experimental setup leads to questions about its relevance to the standard catalytic pathway of iridium-catalyzed olefin hydrogenation.

In totality, the body of experimental evidence for mechanisms in iridium-catalyzed asymmetric hydrogenation is significantly smaller than that available for rhodium-catalyzed asymmetric hydrogenation. In lieu of added experimental evidence, significant computational work has been performed with a range of chiral bidentate ligands and informs the nature of the steps following formation of the iridium alkene dihydride complex. Studies performed by Andersson,^42^ Hall,^48^ Hopmann,^49,50^ Diéguez,^51^ and Neese^52^ all support an Ir­(III/V) mechanism for minimally functionalized olefin hydrogenation including formation of the key iridium alkene–dihydride–dihydrogen complex (Scheme 20). Hopmann and co-workers calculated a pathway for the isomerization of this complex, involving a concerted dihydrogen splitting, proton shuttling, and dihydrogen reformation pathway (Scheme 21). In each case, rate- and enantio-determining migratory insertion occurs from the iridium­(III) alkene–dihydride–dihydrogen complex to form an iridium­(V) alkyl–trihydride species. According to the quadrant model proposed by Andersson and co-workers for trisubstituted olefins, the olefin is oriented so as to put the larger substituents in the least hindered ligand-enforced quadrants in the optimized transition state (Figure 6 bottom).^53^ Results suggest that the nature of the migratory insertion step (pathway A or pathway B) is related to the identity of the ligand. Using PHOX ligands, Andersson, Hopmann, and Neese all suggest that pathway A (concomitant migratory insertion and oxidative addition) is favored. Using *N*-heterocyclic carbene–oxazoline (C,N) and pyranoside phosphite–oxazoline (P,S) ligands respectively, Hall and Diéguez support pathway B (oxidative hydride transfer into the bound dihydrogen). The mechanism is then concluded with reductive elimination and ligand exchange to furnish the hydrogenated product. In each when a full system is studied, the computational results replicate the experimental enantio­selectivities to an acceptable degree.

**Scheme 20:**
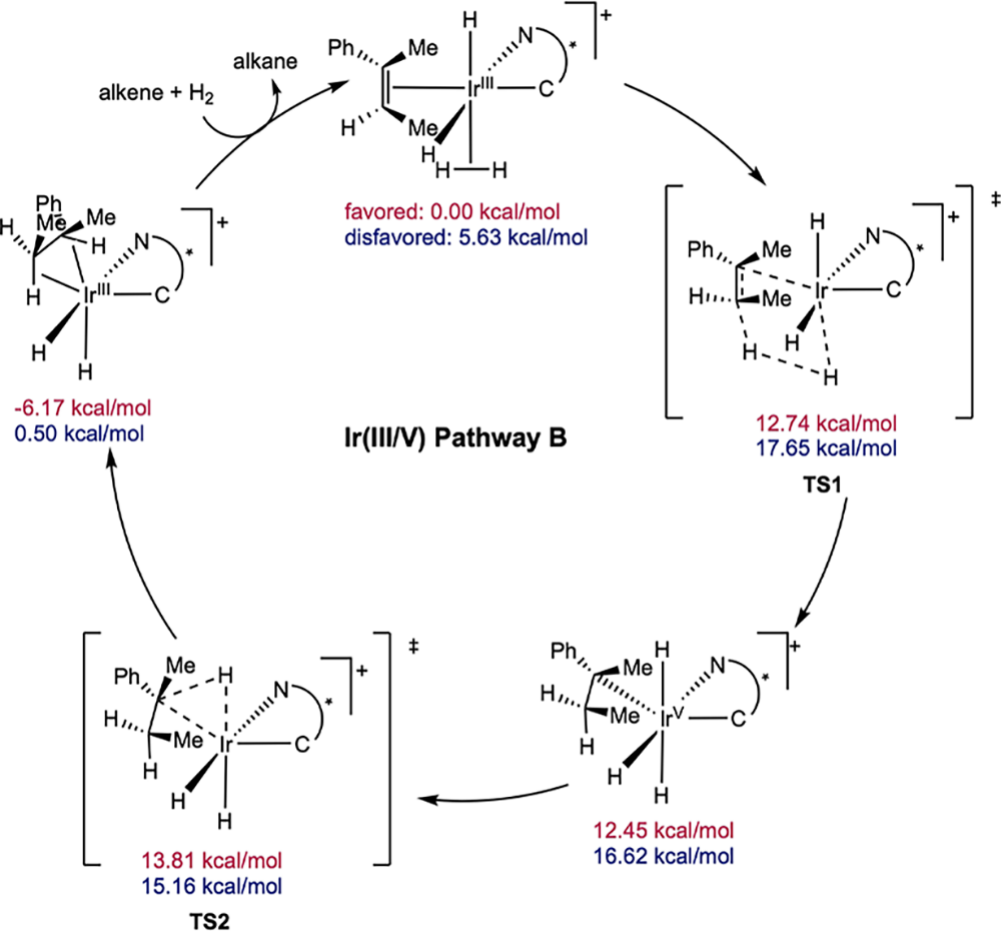
DFT Computed Pathway for *N*-Heterocyclic Carbene–Oxazoline (C,N) Ir-Catalyzed Asymmetric Hydrogenation by Ir­(III/V) Pathway B for the Formation of Favored and Disfavored Enantiomers (Hall, 2004 ^48^)­^a^

a Adapted from ref 48. Copyright 2004 American Chemical Society.

**Scheme 21:**
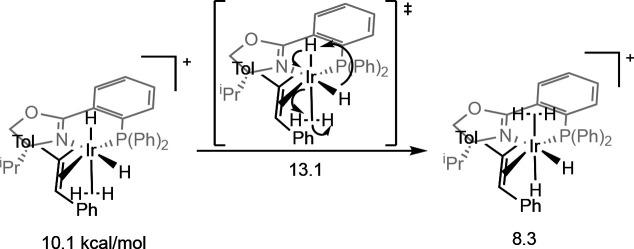
Calculated Isomerization Pathway Proposed for Ir–Dihydrogen–Dihydride–Substrate Complex (Hopmann, 2014 ^50^)­^a^

a Adapted from ref 50. Copyright 2014 American Chemical Society.

**Figure 6:**
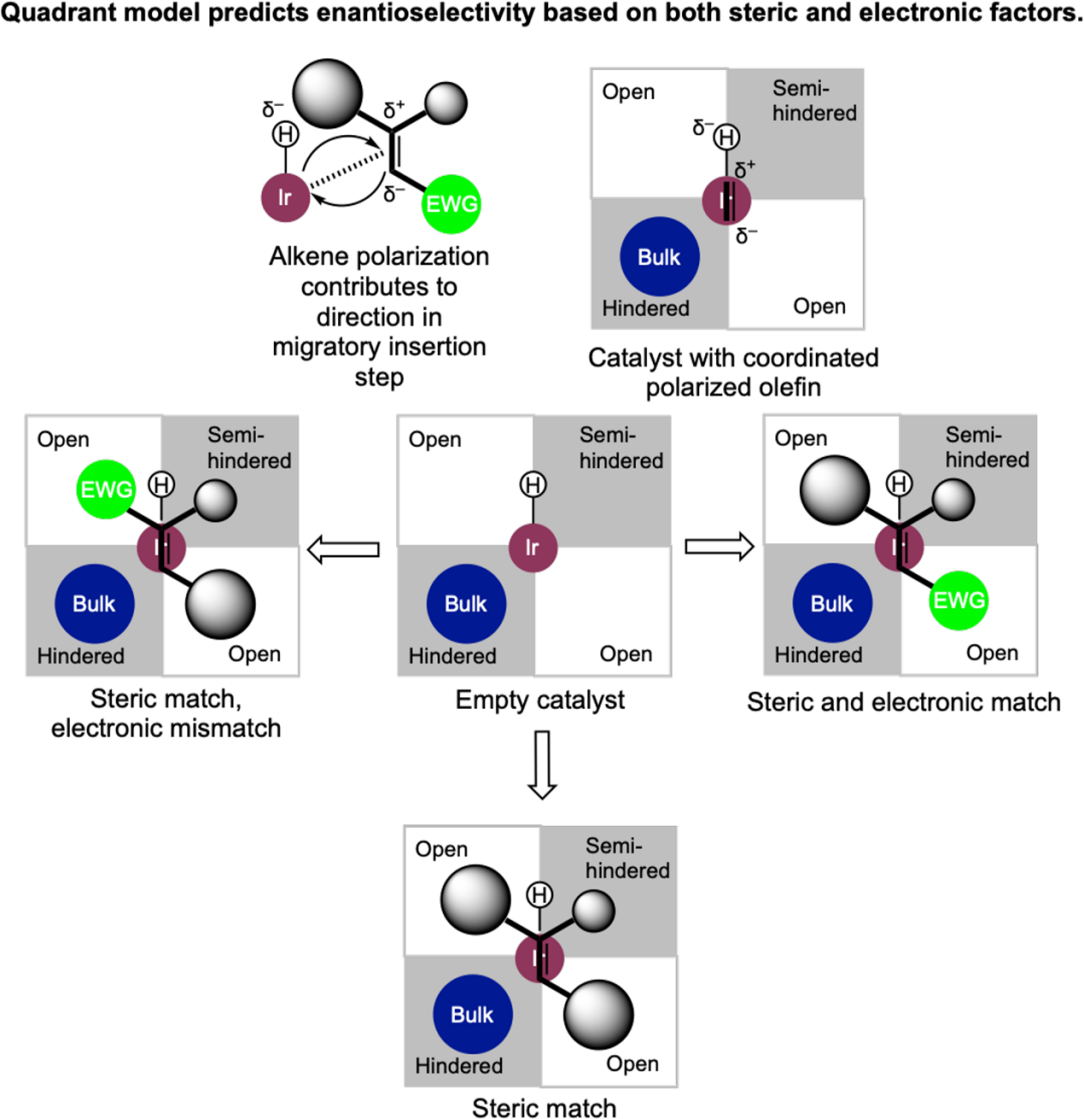
Quadrant model proposed for trisubstituted alkenes with and without electron-withdrawing substituents (Andersson, 2006 ^53^). Adapted from ref 53. Copyright 2006 American Chemical Society.

### Summary of Mechanistic Studies in the Iridium-Catalyzed Asymmetric Hydrogenation of Minimally Functionalized Olefins

In general, there has been strong agreement between experimental and computational mechanistic studies on the iridium-catalyzed asymmetric hydrogenation of minimally functionalized olefins. From these studies, it has become evident that with the examples explored thus far, an Ir­(III/V) cycle is favored when chiral bidentate ligands are used to support the iridium catalyst. Computational studies have distinguished that the nature of the rate-determining step (Pathway A or B) is dependent on the identity of the ligand, although the majority of studies support Pathway A.

### Mechanistic Studies on the Iridium-Catalyzed Hydrogenation of Functionalized Olefins

Iridium-catalyzed asymmetric alkene hydrogenation has primarily been focused on minimally functionalized olefins, as traditional rhodium catalysts typically require two-point binding of the substrate and are therefore less effective. However, iridium catalysts with chiral bidentate ligands have also been applied to the hydrogenations of functionalized substrates including unsaturated furans, alcohols, amides, esters, carboxylic acids, and phosphates.^5^

Before discussing proposed mechanistic pathways in detail, it is worth noting a study performed by Andersson and co-workers in 2006, comparing the outcomes of iridium-catalyzed asymmetric hydrogenation between minimally functionalized olefins, as well as those bearing alcohols and esters.^53^ The phosphine sulfoximine iridium catalysts exhibited much better stereo­selectivity with trisubstituted (*E*)-alkenes and those with electron-withdrawing groups on the less substituted carbon. Assuming the Ir­(III/V) cycle proposed for minimally functionalized olefins was operative for all substrates, the authors amended the quadrant model presented in Figure 6 to include the effect of electron-withdrawing substituents. Namely, calculations of the enantio-determining migratory insertion transition state demonstrated that the electron-withdrawing effect could be attributed to the polarization matching of the hydride-transfer migratory insertion step: sterically, iridium preferentially forms a bond to the less hindered carbon, whereas electronically it preferentially forms a bond to the most electron-poor carbon. When these effects work in concert, higher enantio­selectivities were obtained. While these results do not consider the impact of substrate chelation, they do show that functional groups have the potential to impact the outcome of asymmetric hydrogenation by purely electronic and steric means.

The remaining studies to be discussed all consider the impact of substrate chelation through two-point binding to the iridium center through a heteroatom, as well as the carbon–carbon double bond. In contrast to the mechanism of iridium-catalyzed hydrogenation of minimally functionalized olefins, which is generally accepted to go by an Ir­(III/V) mechanism despite changes to the bidentate ligand, much more mechanistic variability is proposed for the asymmetric hydrogenation of functionalized substrates, particularly those that can participate in two-point binding. A combination of experimental and computational results has led to the conclusion of a variety of mechanisms, including Ir­(III/V), Ir­(I/III), and all Ir­(III) pathways, depending on the ligand and substrate.

An Ir­(III/V) cycle was proposed by Burgess in 2007 for the asymmetric hydrogenation of α,β-unsaturated esters using an NHC–oxazoline (C,N) ligand, which demonstrate facial selectivity opposite to that of allylic alcohols, on the basis of DFT calculations.^54^ This mechanism differs from that proposed for minimally functionalized olefins, due to chelation of the substrate to the iridium center through the carbonyl oxygen throughout the catalytic cycle. In this mechanism, the coordination of a second molecule of dihydrogen is proposed to occur after migratory insertion, rather than before. The mechanism begins with an Ir­(III) substrate dihydride, which directly undergoes migratory insertion to form an Ir­(III) monohydride alkyl in the rate- and enantio-determining step (Scheme 22). A second molecule of dihydrogen then binds to iridium, which undergoes oxidative addition to form an Ir­(V) trihydride alkyl complex, that then reductively eliminates to form the hydrogenated product. In contrast to the Ir­(III/V) mechanism proposed in the previous section lacking chelation, this alternative predicts the experimentally observed facial selectivity.

**Scheme 22:**
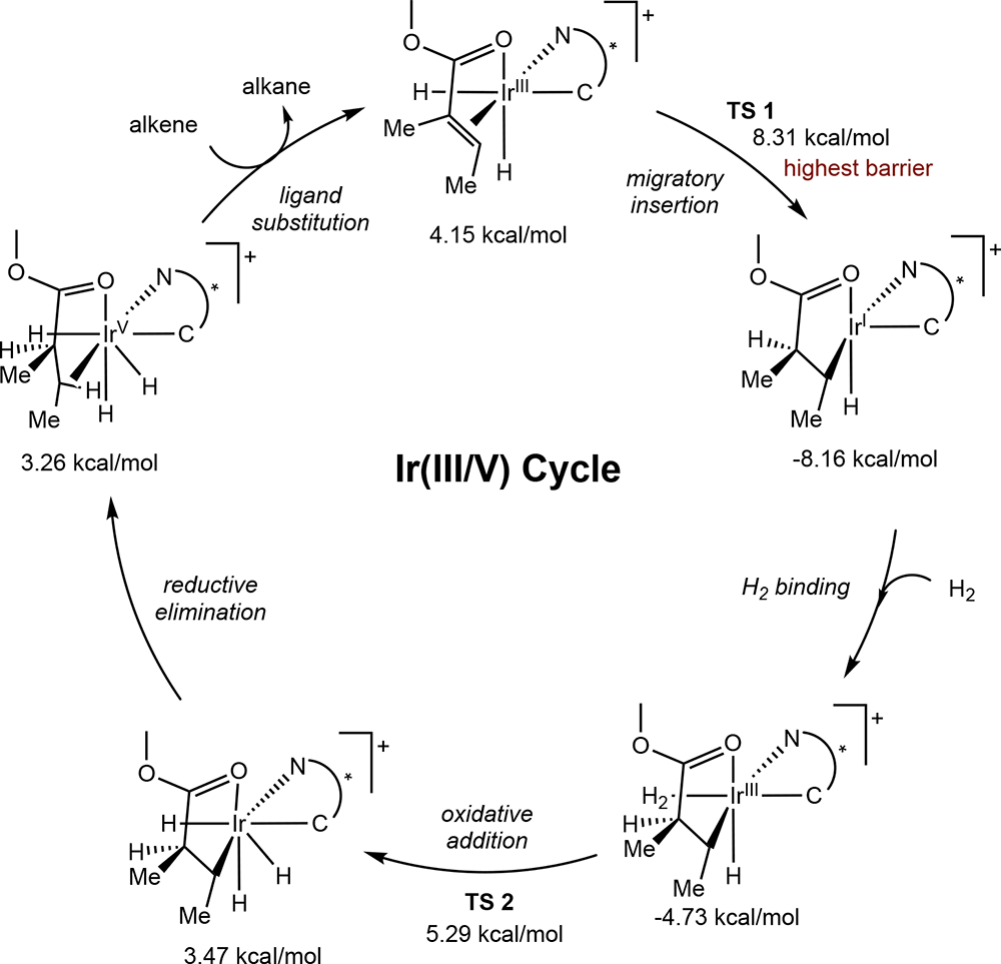
Computed Ir­(III/V) Mechanism for the Hydrogenation of α,β-Unsaturated Carboxylic Acid Derivatives with (C,N) Ir Catalysts (Burgess, 2007 ^54^)­^a^

a Adapted with permission from ref 54. Copyright 2007 John Wiley and Sons.

A similar Ir­(III/V) mechanism was proposed by Zhou in 2017 for the asymmetric hydrogenation of α,β-unsaturated carboxylic acids utilizing modified backbone-chiral spiro phosphine–oxazoline (SIPHOX) and SpiroBAP ligands that is strongly supported by both experimental and computational evidence.^55^ Addition of dihydrogen to a solution of [(SIPHOX)­Ir­(COD)]­[OTf] furnished the dihydride bis­(solvent) iridium complex, [(SIPHOX)­Ir­(MeOH)_2_(H)_2_]­[OTf]. Treatment of this product complex with an α,β-unsaturated carboxylate at room temperature resulted in insertion into an Ir–H bond to yield the corresponding alkyl hydride with the carboxylate oxygen coordinated to the iridium center (Scheme 23). Heating a solution of the resulting alkyl hydride to 65 °C resulted in β-hydride elimination, generating a bridging bimetallic iridium tetrahydride substrate complex, where the carboxylate of the substrate, as well as two hydrides, are bridging the iridium centers. In contrast, addition of dihydrogen to the alkyl hydride at room temperature induces reductive elimination of the alkane, generating a new bimetallic iridium tetrahydride product, where two hydrides and the carboxylate of the product are bridging between the iridium centers. These results pointed to the possibility that additional hydrogen may be required to induce reductive elimination from the monohydride alkyl complex, suggesting an Ir­(III/V) cycle. Deuteration of the monohydride alkyl resulted in 50% deuterium incorporation in the β-position of the product, supporting this hypothesis. DFT calculations also support an Ir­(III/V) cycle starting from the (SIPHOX)­Ir­(H)_2_(substrate)^+^ complex, which undergoes migratory insertion to generate the Ir­(H)­(alkyl). Oxidative addition of H_2_ to this complex generates an Ir­(H)_3_(alkyl), which forms the enantio­enriched product upon reductive elimination and ligand exchange with free substrate.

**Scheme 23:**
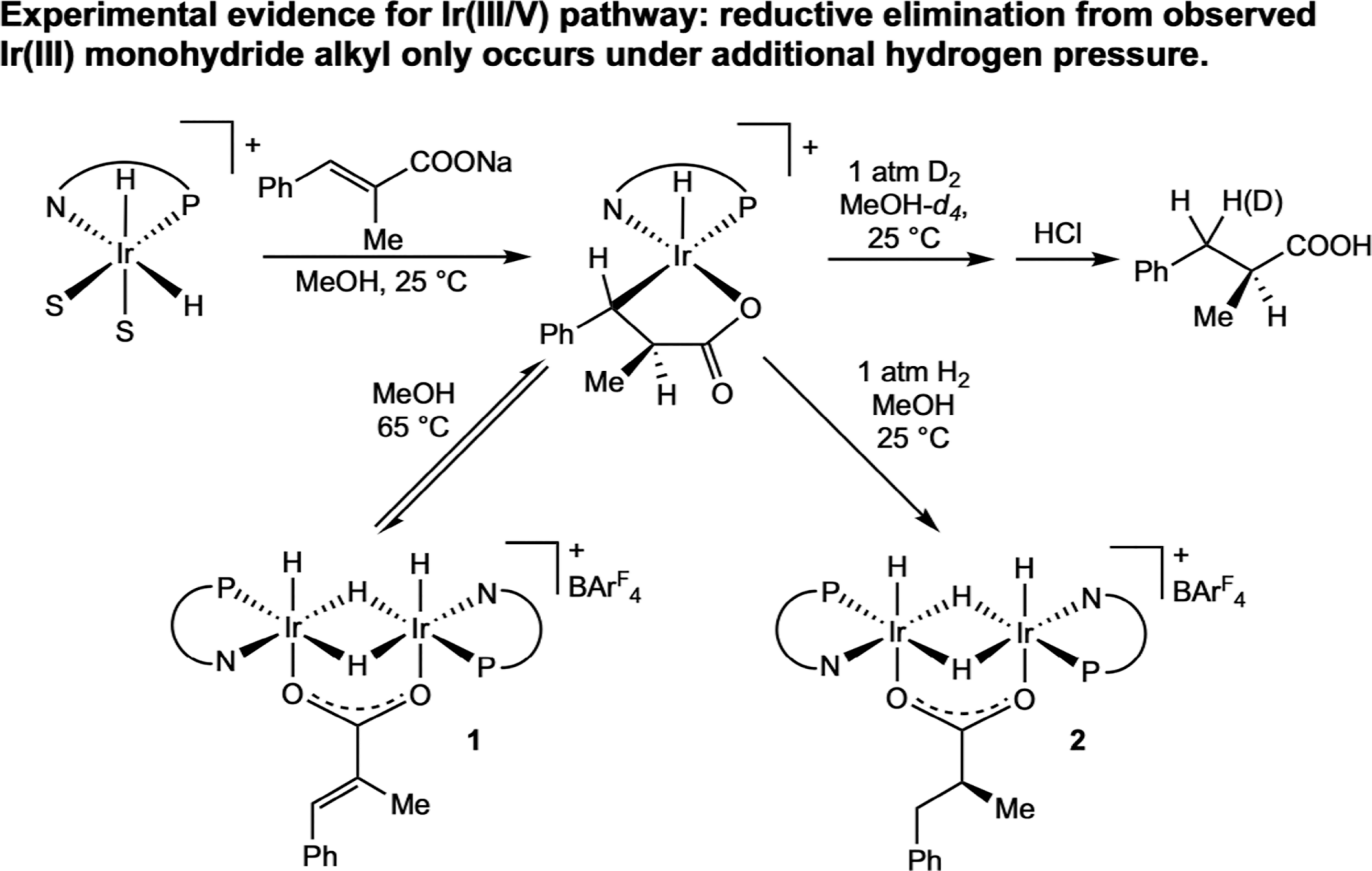
Iridium Complexes Observed by NMR Spectroscopy for the Iridium-Catalyzed Hydrogenation of α,β-Unsaturated Carboxylic Acids Supporting an Ir­(III/V) Mechanism (Zhou, 2017 ^55^)­^a^

a Adapted from ref 55. Copyright 2017 American Chemical Society.

In contrast, an Ir­(I/III) cycle was proposed by Gridnev and Zhang for the asymmetric hydrogenation of α,β-unsaturated exocyclic carbonyl compounds and by Diéguez for the asymmetric hydrogenation of tetrasubstituted acyclic enones, supported primarily by DFT calculations.^56,57^ Both mechanisms begin from an Ir­(III) dihydride substrate complex, where the substrate is chelated through the carbonyl oxygen (Scheme 24). In Gridnev and Zhang’s work, attempts to calculate structures with an additional dihydrogen bound to the complex all led to dissociation of the dihydrogen, leading to the conclusion that an Ir­(I/III) cycle must be operative. Diéguez considered a variety of Ir­(III/V) and all Ir­(III) pathways, but determined that neither adequately fit the experimental results, whereas a computed Ir­(I/III) cycle did. In both studies, migratory insertion occurs directly from the Ir­(III) dihydride substrate complex to form an Ir­(III) monohydride alkyl, followed by direct reductive elimination to form an Ir­(I) product species. The resting state is regenerated through oxidative addition of H_2_ and ligand substitution with a substrate molecule.

**Scheme 24:**
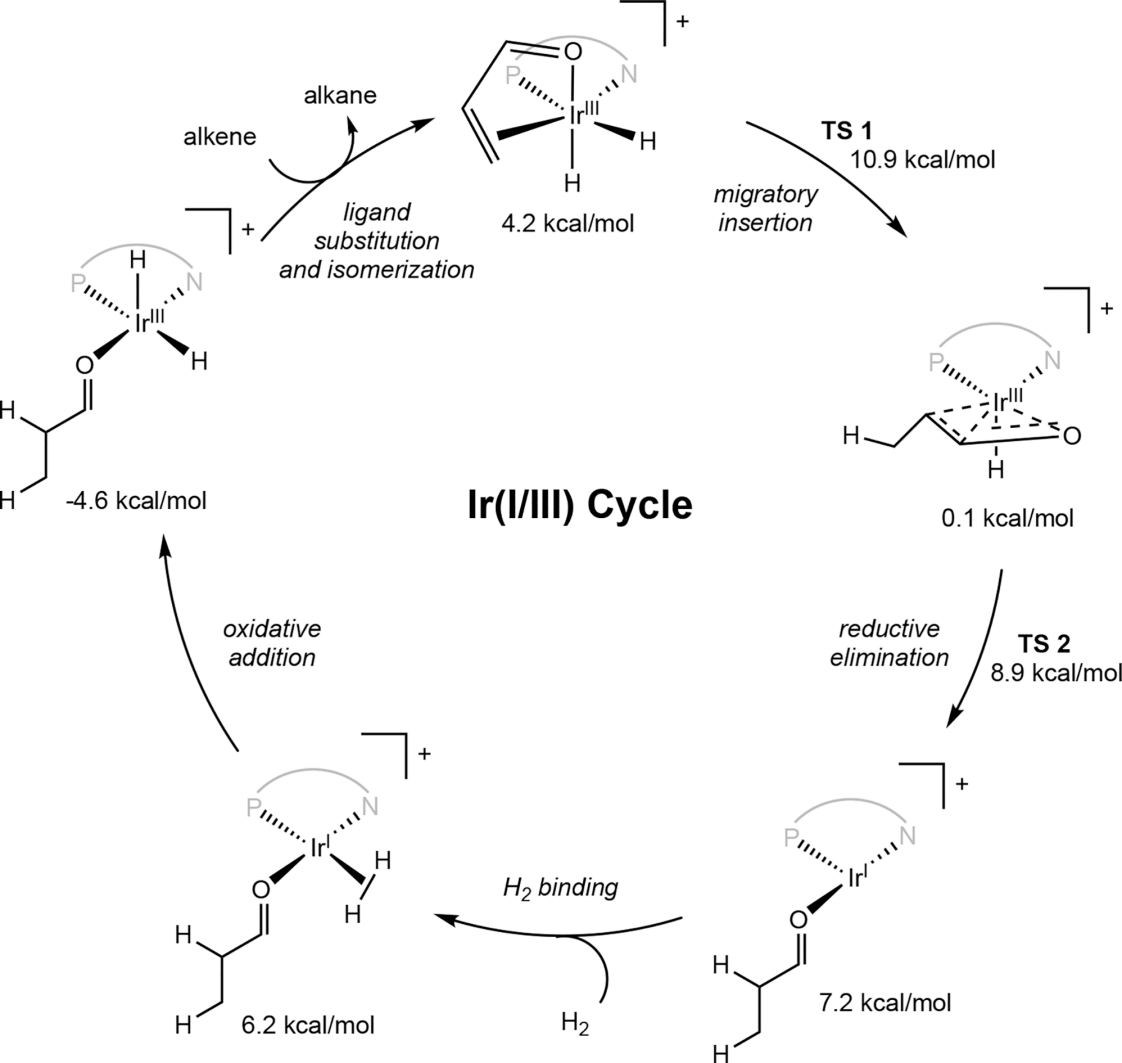
Simplified Ir­(I/III) Cycle Calculated for the Asymmetric Hydrogenation of a Tetrasubstituted Enone (Diéguez, 2024 ^57^)

Lastly, an all Ir­(III) cycle was proposed by Norrby and Bolm in 2016 for the asymmetric hydrogenation of α,β-unsaturated ketones and by Andersson in 2021 for the asymmetric hydrogenation of enamides using computational results.^58,59^ In Norrby and Bolm’s study, both Ir­(I/III) and Ir­(III/V) pathways were considered, but the authors found that an all Ir­(III) pathway was preferred. This pathway initiates from an iridium dihydride substrate complex, where the α,β-unsaturated ketone is coordinated to the iridium through the C=C bond as well as through the oxygen of the carbonyl (Scheme 25). Initial migratory insertion to transfer a hydride to the β-carbon occurs, followed by rearrangement, and then coordination of free dihydrogen to form an alkyl–hydride–dihydrogen intermediate. Finally, a metathesis-type transition state transfers a proton to the substrate from the bound dihydrogen, forming a final product–dihydride. Here, the most energetically costly step is the metathesis step to form the fully hydrogenated product, suggesting that the initial migratory insertion to form the iridium monohydride alkyl should be reversible. This is in contrast to the previous two pathways proposed, where the migratory insertion step is proposed to be enantio-determining.

**Scheme 25:**
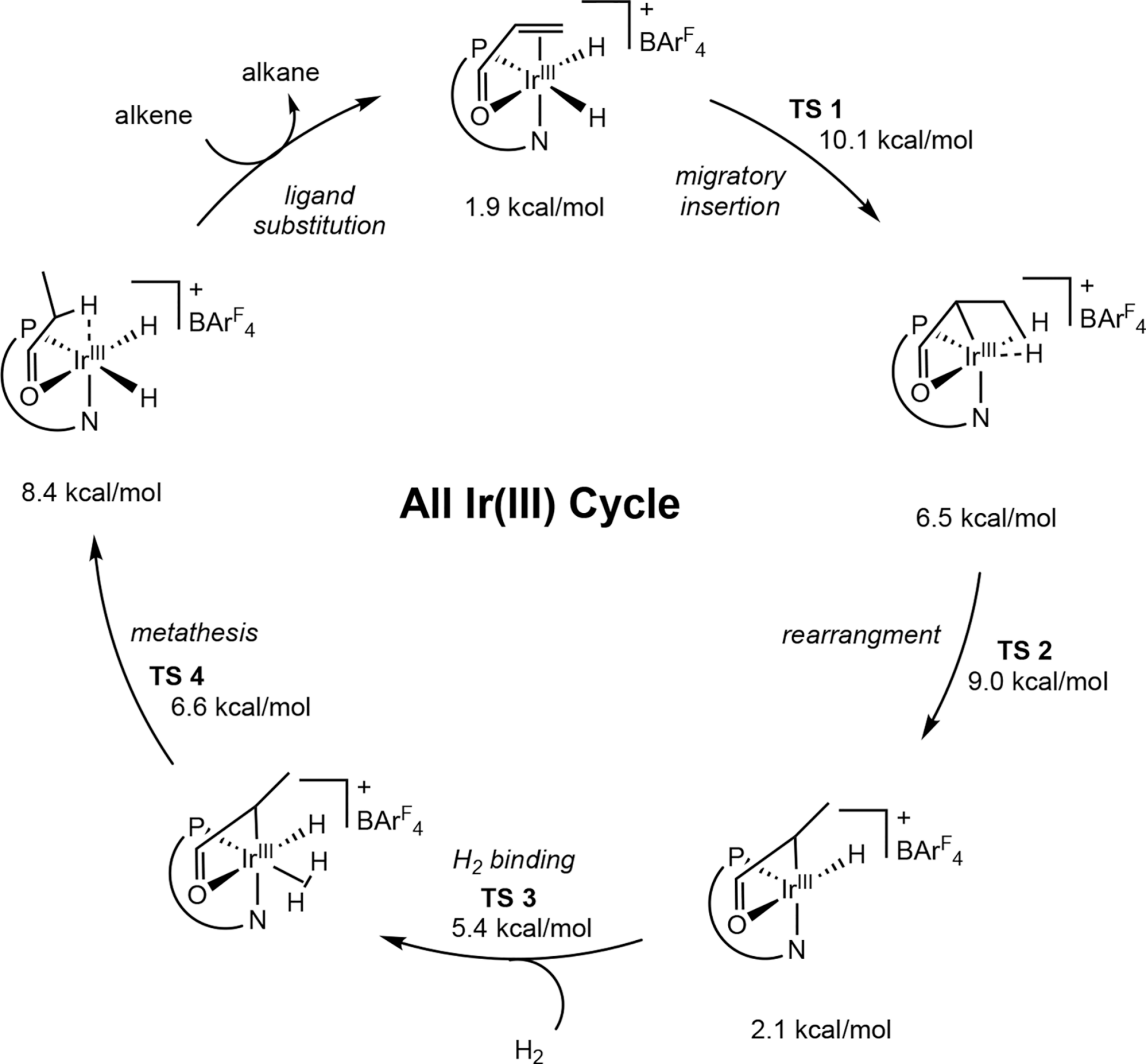
Simplified All-Ir­(III) Cycle Calculated for the Asymmetric Hydrogenation of α,β-Unsaturated Ketones (Norrby and Bolm, 2016 ^58^)­^a^

a Adapted from ref 58. Available under a CC-BY license. Copyright 2023 Jorge Faiges, Maria Biosca, Miquel A. Pericàs, et al.

Andersson proposed a similar mechanism for the asymmetric hydrogenation of enamides.^59^ In this work, the all-Ir­(III) mechanism with reversible migratory insertion was used to explain different outcomes in enantio­selectivity among four different classes of trisubstituted enamides. For example, the (*E*) and (*Z*) isomers of α,β-diaryl enamides undergo convergent enantio­selectivity, while α-aryl,β-alkyl enamides only do so under low hydrogen pressures and high temperatures (Scheme 26). By kinetically tracking the formation of organic reaction intermediates, in combination with deuterium labeling studies and DFT calculations, the authors concluded that this difference is a result of α,β-diaryl enamides undergoing iridium-catalyzed isomerization at a significantly faster rate than hydrogenation, while with α-aryl,β-alkyl enamides this process occurs at a slower rate that is competitive with hydrogenation. These results are in agreement with the all Ir­(III) mechanism proposed by the authors and could not be explained by a mechanism where migratory insertion is rate-limiting.

**Scheme 26:**
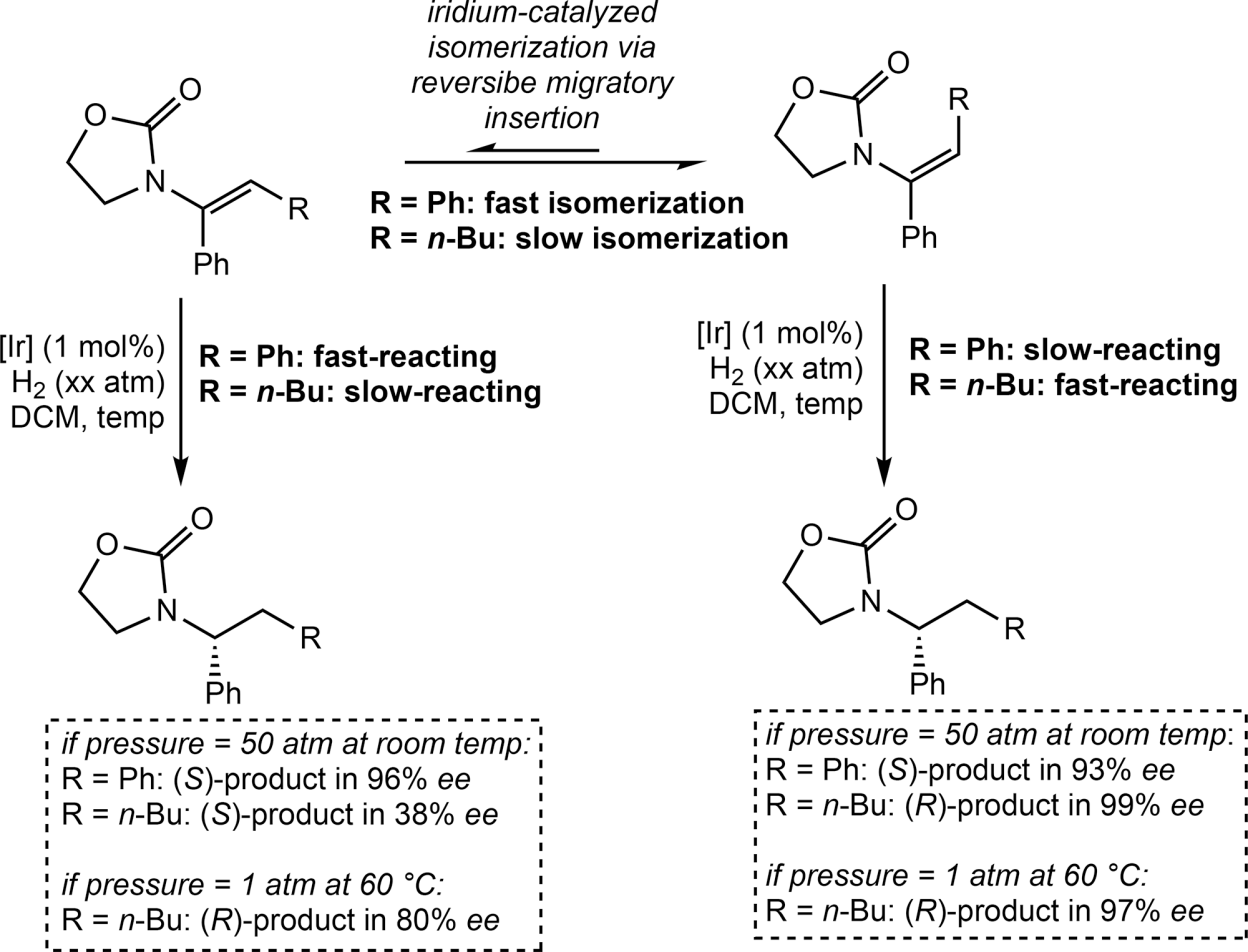
Divergent Outcome for Iridium-Catalyzed Asymmetric Hydrogenation of Enamide Substrates Explicable by an All-Ir­(III) Mechanism with Reversible Migratory Insertion (Andersson, 2021 ^59^)­^a^

a Adapted from ref 59. Available under a CC-BY 4.0 license. Copyright 2021 Jiangping Yang, Luca Massaro, et al.

A key intermediate in all three of these proposed mechanisms is a monomeric iridium monohydride alkyl. Although not necessarily relevant to catalysis, Maurer and Kazmeier isolated such a complex in 2013 with a β-amido ketone substrate (Scheme 27).^60^ The authors found that β-amido ketones were not only unreactive toward hydrogenation but also resulted in an erosion of catalyst activity when mixed with otherwise active substrates. Mixing the [(P,N)­Ir­(COD)]­[BAr^F^_4_] precatalyst with an unsaturated β-amido ketone in the presence of dihydrogen resulted in the formation of a new diamagnetic complex, which was identified as a σ-alkyl iridium hydride complex where both carbonyl oxygens were coordinated to the iridium center. This hapticity forms an 18-electron complex, which suppresses reductive elimination and inhibits catalysis but enables observation of an elusive intermediate that was previously implicated but unobserved in iridium-catalyzed hydrogenations. This study represents both a pitfall of chelating functionality in iridium catalysis, as well as use of that same chelation as a potential mechanistic tool for isolating otherwise elusive intermediates.

**Scheme 27:**
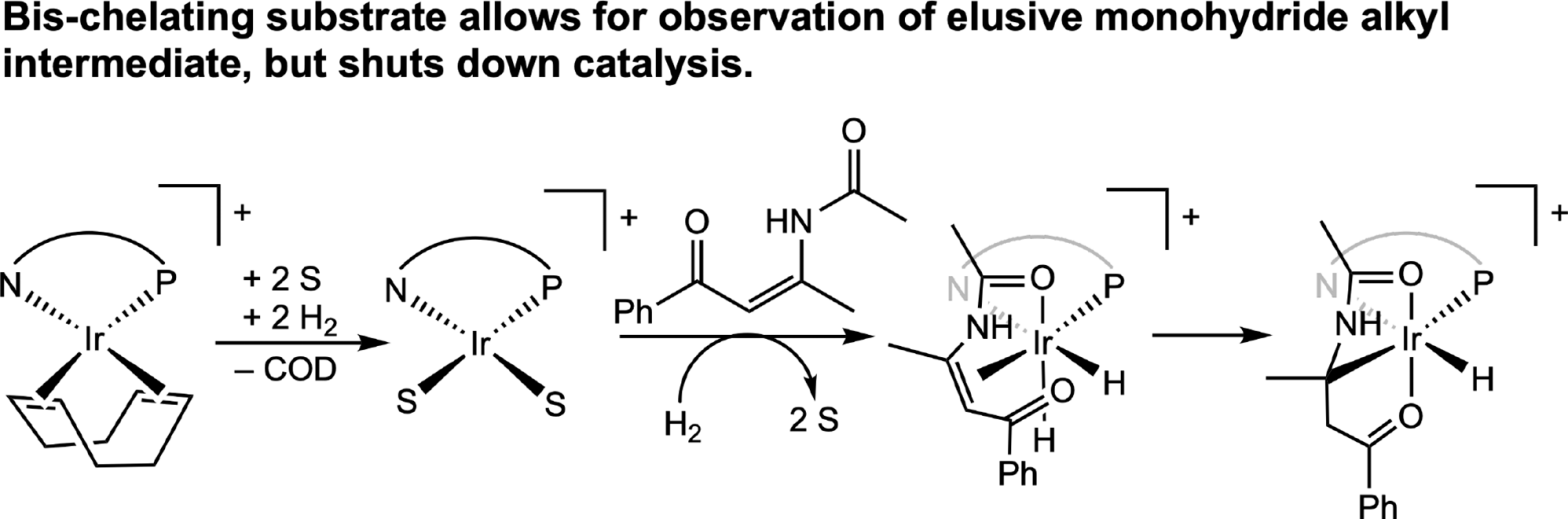
Isolation of a Monomeric Iridium Monohydride Alkyl (Maurer and Kazmeier, 2013 ^60^)­^a^

a Adapted from ref 60. Copyright 2013 American Chemical Society.

### Summary of the Mechanistic Studies on the Iridium-Catalyzed Asymmetric Hydrogenation of Functionalized Alkenes

The varied results in the above studies demonstrate how polar and coordinating functionalities have a profound effect on the preferred mechanism of iridium-catalyzed asymmetric hydrogenation. Substrate functional groups, as well as ligand choice, play a significant role in mechanistic preference and ultimately the oxidation states accessed during the catalytic cycle. In contrast to minimally functionalized olefins, based on the small set of mechanistic studies discussed above, a single overarching mechanistic pathway is not operative, even when either the same ligand or the same class of substrates is used.

## Cobalt

### Brief Overview of Cobalt-Catalyzed Asymmetric Olefin Hydrogenation

Asymmetric catalysis utilizing first-row transition metals, in comparison to their second- and third-row counterparts, has been less extensively studied. Interest in developing more sustainable chemistry has resulted in a marked increase in the study and use of earth-abundant metals in catalysis. Because of the lower *d*-orbital splitting energy, first-row transition-metal complexes form both high- and low-spin complexes and access more oxidation states, including those separated by one electron. While rhodium catalysis reliably cycles between I/III oxidation states through oxidative addition and reductive elimination, isolable cobalt precatalysts in the −I, 0, I, II, and III oxidation states have been synthesized (Figure 7). These fundamental differences in atomic properties offer the opportunity for new reaction mechanisms.^6^ While important examples of cobalt-catalyzed asymmetric hydrogenation have been reported using tridentate pincer ligands,^61,62^ this Tutorial will be limited to examples employing chiral bidentate phosphines for mechanistic comparison to the widely studied rhodium catalysts.

**Figure 7:**
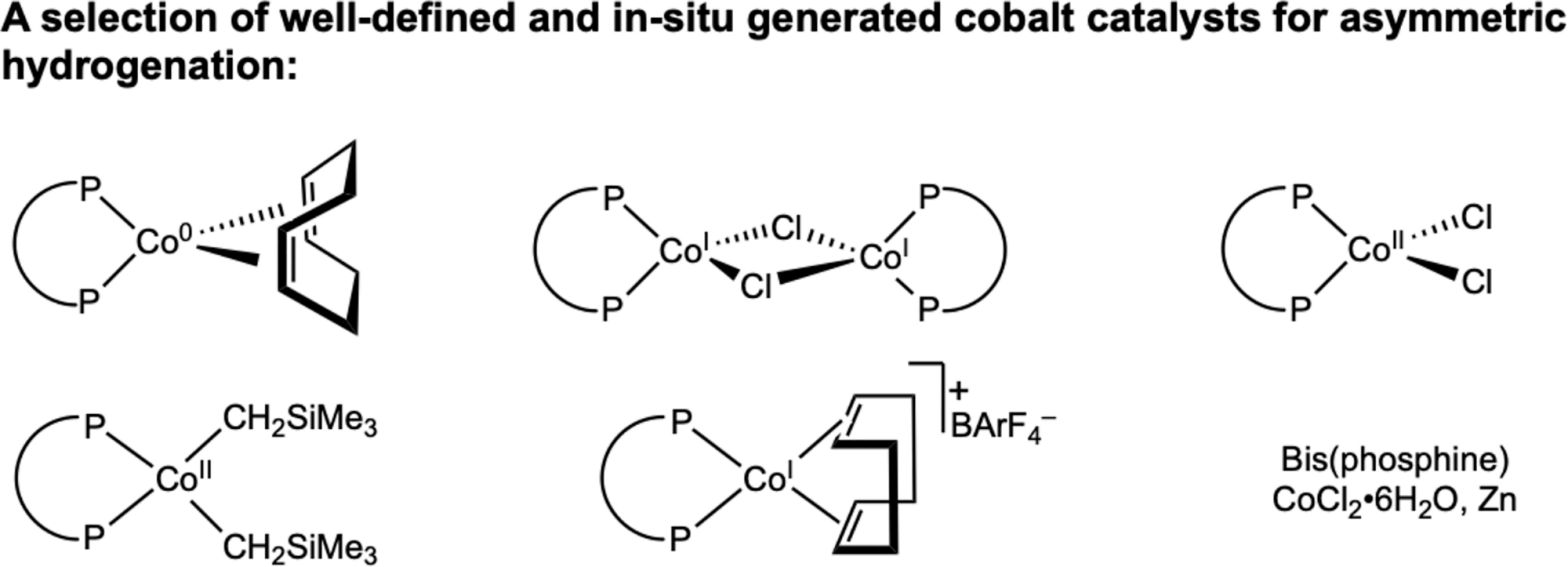
Bis­(phosphine) cobalt precatalysts in the 0, I, and II oxidation states.^63–65^

In 2013, our laboratory reported the synthesis of chiral bis­(phosphine) cobalt­(II) bis­(neosilyl) complexes as precatalysts for the asymmetric hydrogenation of prochiral enamides and minimally functionalized alkenes.^63^ In contrast to cationic rhodium and iridium precatalysts that are typically used in asymmetric alkene hydrogenation, the cobalt precatalysts were neutral. In 2018, a more convenient and robust catalyst activation mode was reported, where the combination of bidentate phosphine and common cobalt salts, such as CoCl_2_·6H_2_O were combined with excess zinc as a single-electron reductant.^64^ This approach has enabled high activity and selectivity for the hydrogenation of prochiral enamides with a much larger scope of bis­(phosphine) ligands as compared to the previously reported method from well-defined cobalt dialkyl complexes. Interestingly, the highest activity was observed in protic solvents, such as methanol that is environmentally preferred but has typically been deleterious in catalysis with first-row transition metals. Using the zinc-reduction method in methanol, well-defined neutral cobalt precatalysts have been isolated in the 0, I, and II oxidation states. In 2019, our laboratory reported synthesis of cationic bis­(phosphine) cobalt­(I) complexes, which also exhibited exceptional performance for the hydrogenation of prochiral enamides.^65^ Bis­(phosphine) cobalt catalysis across both oxidation states has demonstrated remarkable functional group tolerance, having so far been applied to the asymmetric hydrogenation of minimally functionalized olefins,^63^ enamides,^64^ α,β-unsaturated carboxylic acids,^66–68^ hydrazones,^69^ enynes,^70^ cyclic enamides,^71^ and azole-containing alkenes (Scheme 28).^72^

**Scheme 28:**
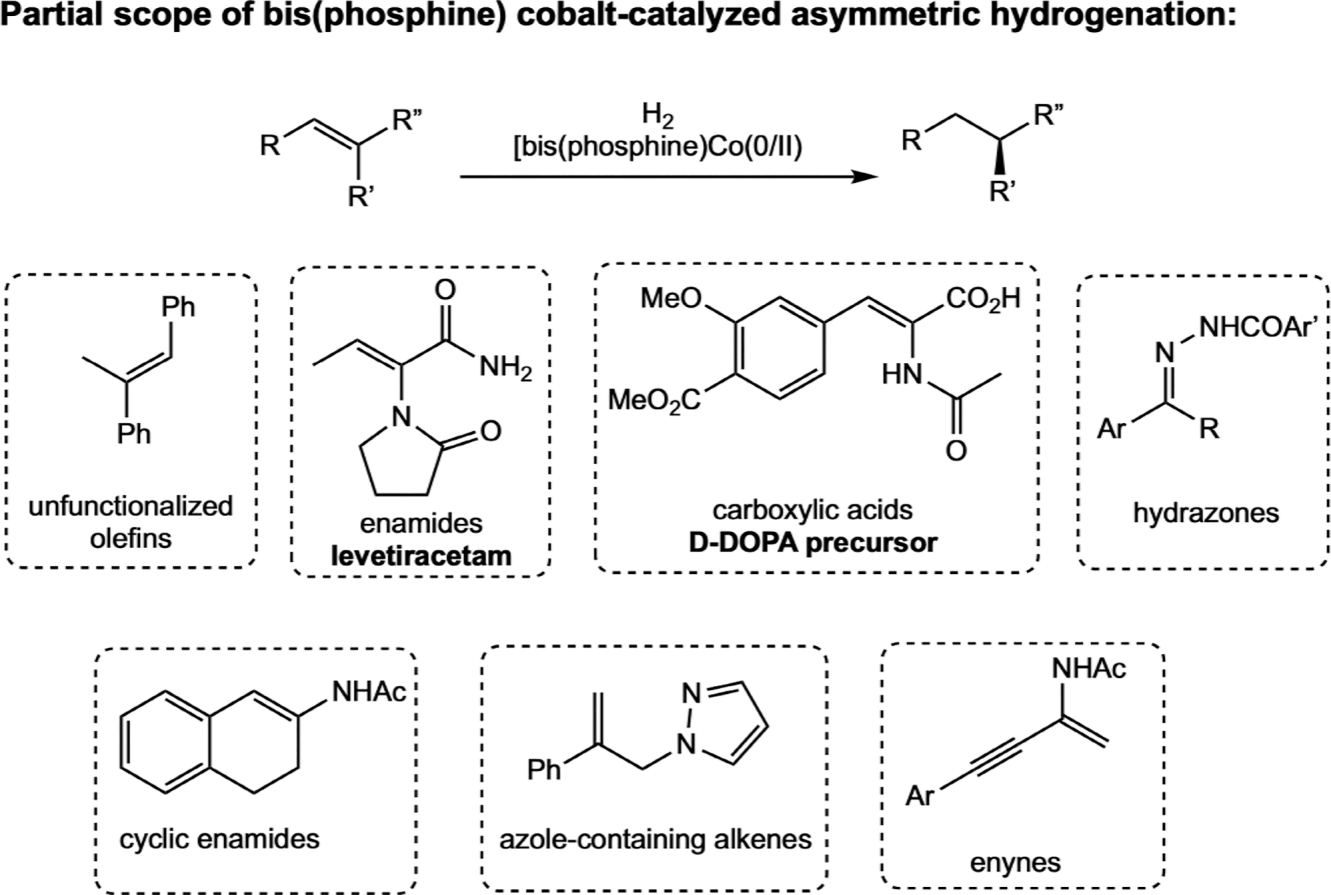
Classes of Substrates Reported in Cobalt-Catalyzed Asymmetric Hydrogenation^63–69,72^

While bis­(phosphine) cobalt complexes perform comparably to their rhodium counterparts in asymmetric hydrogenation, additional questions have arisen for the cobalt-catalyzed mechanism due to the larger number of accessible oxidation states. The role of various oxidation states, potential one-electron pathways, and redox cycling must all be considered. While the literature on the mechanism of bis­(phosphine) cobalt-catalyzed asymmetric hydrogenation is still in its infancy, recent experimental and computational work has shed light on some of these key mechanistic questions.

### Mechanistic Proposals for Neutral Cobalt-Catalyzed Asymmetric Alkene Hydrogenation

Because the neutral Co­(0/II) precatalysts have received more attention and mechanistic scrutiny than the cationic cobalt­(I) variants, the former will be discussed in more detail. While several mechanisms have been proposed for neutral bis­(phosphine) cobalt-catalyzed asymmetric hydrogenation of olefins, only two have been supported by both experimental and computational data. In all examples studied thus far with neutral, paramagnetic cobalt precatalysts, asymmetric hydrogenation is proposed to occur through an unsaturated pathway, where the cobalt center first coordinates the olefin before the addition of H_2_. This is preferred despite the presence of relatively electron-donating ligands. Among the possible mechanisms, Pathway A is a traditional, two-electron pathway cycling between Co(0) and Co­(II) (Scheme 29A). This mechanism is like that proposed for rhodium-catalyzed asymmetric hydrogenation, save for the reduction of the metal by one-electron and the intermediacy of paramagnetic compounds. As such the interaction of substrate with H_2_ likely proceeds by oxidative hydride transfer through direct metathesis-type insertion from a σ-bound dihydrogen complex rather than by stepwise oxidative addition and migratory insertion. This furnishes a Co­(II) alkyl hydride, which following reductive elimination, yields the hydrogenated product.

**Scheme 29:**
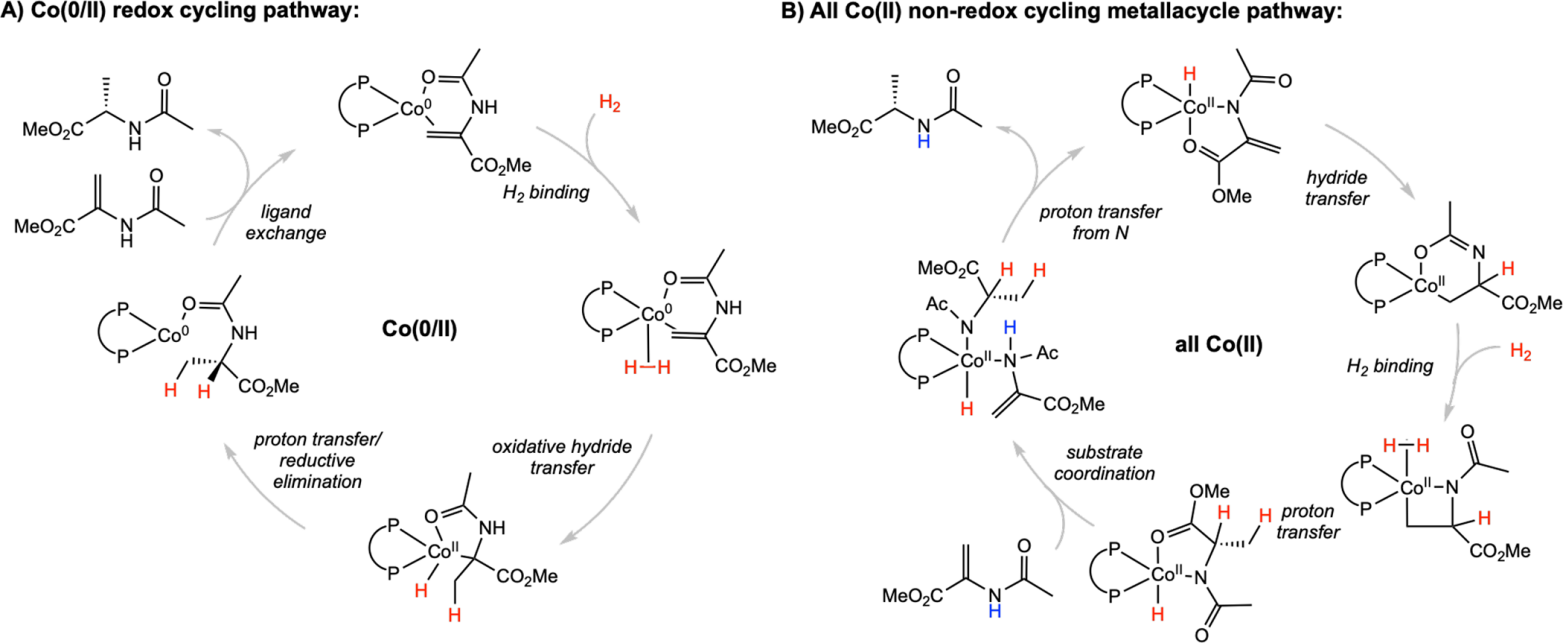
Mechanistic Proposals for Cobalt-Catalyzed Asymmetric Hydrogenation: (A) Two-Electron Redox Cycling between Co(0) and Co­(II) and (B) Redox-Neutral Cobalt­(II) Pathway Involving Metallacycles

An alternative is Pathway B that operates through a redox neutral mechanism involving cobalt­(II) complexes (Scheme 29B). This pathway is only possible for the hydrogenation of substrates containing proximal X–H groups (X = heteroatom). Formal oxidative addition of the X–H bond generates a Co­(II)­(X-olefin)­(H) compound that undergoes migratory insertion/hydride transfer to form a Co­(II)­(alkyl)­(X) metalla­cycle. Coordination of dihydrogen generates a σ-bound complex, which can transfer a proton to the alkyl through a σ-bond metathesis transition state to form a Co­(II)­(X-product)­(H) compound. Turnover occurs upon proton exchange with free substrate and release of product. This pathway is related to that proposed by Norrby and Bohm for the iridium-catalyzed hydrogenation of α,β-unsaturated carboxylic acids^58^ but has no precedent in the rhodium hydrogenation literature, highlighting how the metal center may play a key role in determining mechanism.

Strong evidence has been presented to support both pathways A and B for neutral cobalt-catalyzed asymmetric alkene hydrogenation, dependent on both the ligand and the substrate. Due to the paramagnetism of many reaction intermediates, techniques such as EPR and UV/vis spectroscopy, X-ray crystallography, and DFT calculations have been relied on, in lieu of the diamagnetic NMR spectroscopy frequently employed in the study of rhodium-catalyzed asymmetric hydrogenation. The Co­(0/II) redox-cycling pathway A has been proposed for both the asymmetric hydrogenation of enamides,^74–76^ as well as the asymmetric hydrogenation of allylic azoles.^72^ All-Co­(II) metallacycle pathway B has been proposed for the asymmetric hydrogenation of enamides^76^ and α,β-unsaturated carboxylic acids,^66–68^ as well as for the diastereo­selective hydrogenation of unsaturated alcohols.^73^ This discussion will commence with studies of the asymmetric hydrogenation of enamides, for direct comparison with rhodium.

### Mechanistic Studies on Cobalt-Catalyzed Asymmetric Olefin Hydrogenation of Enamides

Enamides are thus far the most studied substrate for cobalt-catalyzed asymmetric hydrogenation, owing in part to the observed high activities and enantiomeric excesses. As in rhodium catalysis, a key question is whether cobalt reacts first with enamide by an unsaturated pathway or with hydrogen through a dihydride pathway. In 2022, our laboratory sought to answer this fundamental question by targeting the synthesis of both cobalt(0) enamide and cobalt­(II) dihydride complexes, the key intermediates along each respective pathway.^74^

A series of (*R*,*R*)-(^*i*Pr^DuPhos)­enamide ((*R*,*R*)-^iPr^DuPhos = (+)-1,2-bis­[(2*R*,5*R*)-diisopropyl­phospholano]­benzene) complexes bearing each of three representative enamides was independently synthesized through the consecutive reactions of (*R*,*R*)-(^*i*Pr^DuPhos)­Co­(CH_2_SiMe_3_)_2_ with H_2_, followed by addition of the enamide (Scheme 30A). Notably, the solid-state structures of all three formally Co(0) enamide complexes established the formation of the “wrong” pro-(*R*) diastereomer, as compared to the (*S*)-enantiomers obtained from asymmetric hydrogenation of these substrates. While the NMR spectra of these complexes were broad and featureless, their EPR spectra were informative. A diamagnetic bridging dicobalt tetrahydride dimer, [(*R*,*R*)-(^*i*Pr^DuPhos)­Co]_2_(μ_2_-H)_3_(H), was also isolated and characterized by NMR spectroscopy and X-ray diffraction (Scheme 30B). Evaluation of both the cobalt(0) enamides and dicobalt­(II) tetrahydride dimer in stoichiometric and catalytic reactions supported that the Co(0) enamide was more likely relevant to catalysis. Resting state analyses by UV–visible and freeze–quench EPR spectroscopies demonstrated that the Co(0) enamide complexes were indeed the catalyst resting states, consistent with an unsaturated mechanism (Scheme 30C). Also consistent with an unsaturated mechanism was the experimental rate law, which demonstrated a first order dependence on cobalt and H_2_ and a zeroth order dependence on enamide.

**Scheme 30:**
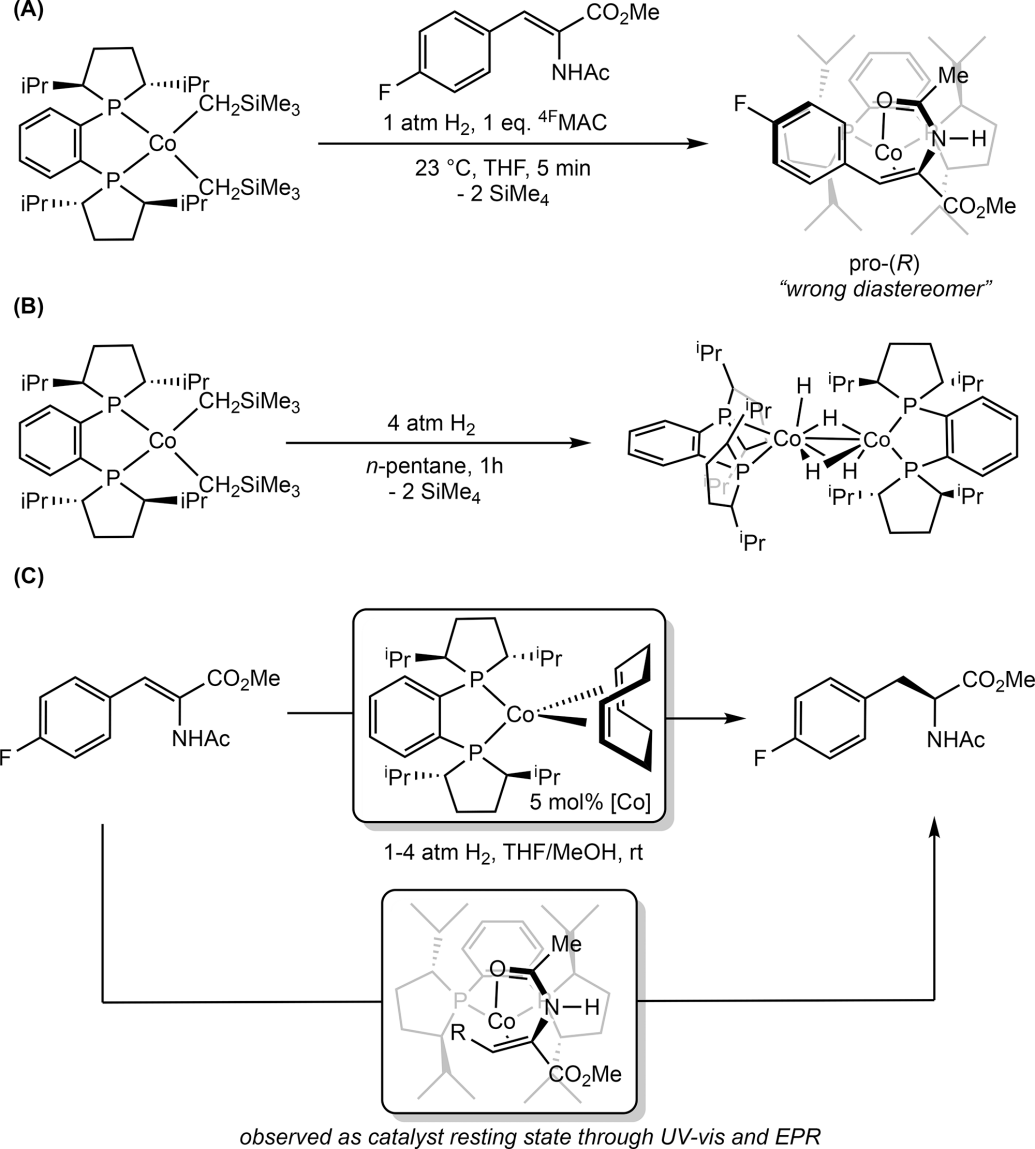
Experimental Evidence for an Unsaturated Mechanism in Cobalt-Catalyzed Enamide Hydrogenation (Chirik and Hopmann, 2022 ^74^)­^a^

a Adapted from ref 74. Copyright 2022 American Chemical Society.

In this study, no additional intermediates along either Pathway A or Pathway B were experimentally observed. Therefore, a combination of deuterium labeling studies, as well as DFT calculations performed by the Hopmann laboratory, were used to distinguish between these two and other mechanisms. Deuterium labeling experiments in MeOH demonstrated exclusive 1,2-*d*_*2*_-incorporated product formation (Scheme 31A). An H_2_/D_2_ KIE demonstrated a value very similar to that shown for cationic bis­(phosphine) rhodium-catalyzed asymmetric hydrogenation of enamides (Scheme 31B).^28^ HD labeling experiments demonstrated preferential deuterium incorporation in the α-position of the enamide, supporting a 2,1-insertion step (Scheme 31C). DFT calculations on the Gibbs free energies of the full catalyst profile supported an unsaturated Co­(0/II) redox cycling mechanism A, commencing from a Co(0) enamide complex (Scheme 32). Here, the rate- and enantio-determining step is the oxidative hydride transfer to the β-carbon of the enamide from a cobalt enamide dihydrogen complex, in agreement with the experimental rate law and deuterium labeling. This pathway provides the hydrogenated product with a predicted 98% *ee*, in excellent agreement with the 98% *ee* observed experimentally using either (*R*,*R*)-(^*i*Pr^DuPhos)­Co­(COD) or (*R*,*R*)-(^*i*Pr^DuPhos)­Co­(enamide) as precatalyst. Directed, redox-neutral Co­(II) metallacycle mechanism B and Co­(0/II) enamide-to-imine tautomerization pathways, as well as a dihydride pathway, could be ruled out by the computations.

**Scheme 31:**
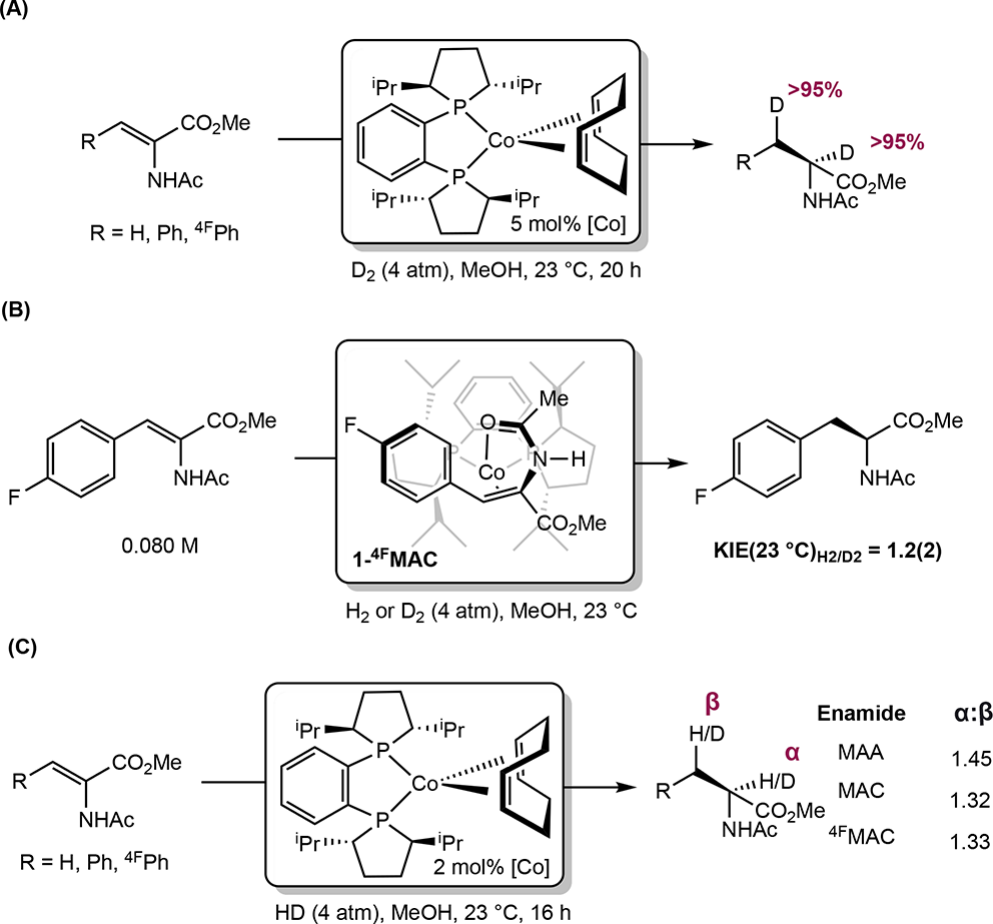
Deuterium Labeling Studies: (A) Deuteration of Enamides, (B) H_2_/D_2_ KIE Measurement, and (C) HD Labeling Experiments (Chirik and Hopmann, 2022 ^74^)­^a^

a Adapted from ref 74. Copyright 2022 American Chemical Society.

**Scheme 32:**
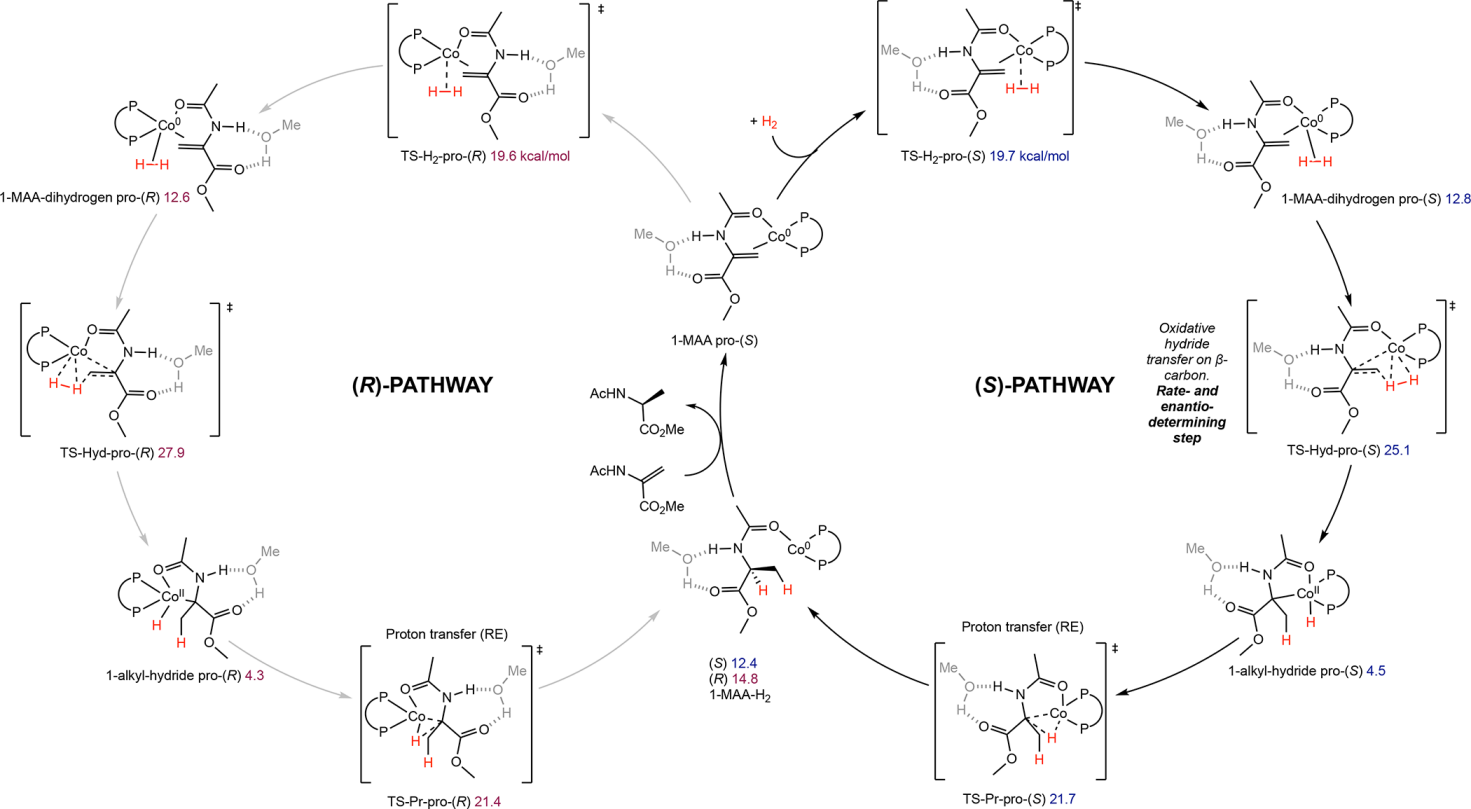
DFT-Computed Mechanism for the Asymmetric Hydrogenation of MAA by (*R*,*R*)-(^*i*Pr^DuPhos)­Co­(COD) (Chirik and Hopmann, 2022 ^74^)­^a^

a Adapted from ref 74. Copyright 2022 American Chemical Society.

A Co­(0/II) redox cycling pathway A for enamide hydrogenation was also supported by Wanbin Zhang’s group in 2023 for the (*S*,*S*)-(^Ph^BPE)­Co-catalyzed ((*R*,*R*)-^Ph^BPE = (+)-1,2-bis­((2*R*,5*R*)-2,5-diphenyl­phospholano)­ethane) enantio­convergent asymmetric hydrogenation of *E*/*Z* enamides.^75^ Here, the strategy was to find a catalyst that would hydrogenate both the *E*- and *Z*-alkenes to provide the product with the same major enantiomer (92% *ee* and 99% *ee* (*S*), respectively). DFT studies support mechanism A, similar to that proposed in the previous example. Oxidative hydride transfer is proposed to be the stereo­determining step, where the proton adds to the β-position of the alkene. A quadrant model was proposed that explains the convergent enantio­selectivity (Figure 8). In both conformers, the nature of the two-point coordination of the substrate makes the methyl group responsible for distinguishing between the (*R*) and (*S*) configurations. For *Z*-conformers, the aryl group extends out away from the cobalt center, meaning that placing the methyl group in the less hindered quadrant is strongly distinguishing. In the *E*-conformers, the aryl group sits to the side, generating large amounts of steric hindrance in both the pro-(*R*) and pro-(*S*) configurations. This pushes the substrate away from the cobalt center. While the methyl group is still enantio-distinguishing, it is a weaker interaction, and so the energy difference between the two possible transition states is lower. This rationale explains why the two conformers react to give the same major enantiomer, but also why the enantio­selectivity is higher for the (*Z*)-isomers with this ligand.

**Figure 8:**
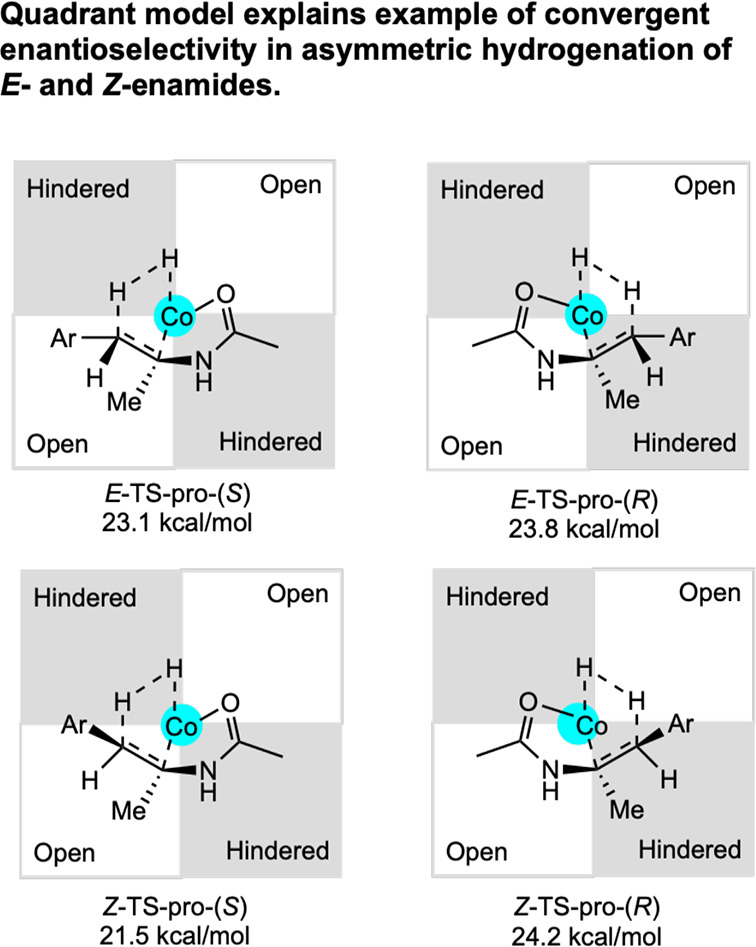
Quadrant model for the convergent asymmetric hydrogenation of *E*- and *Z*-enamides (Zhang, 2023 ^75^). Adapted with permission from ref 75. Copyright 2023 John Wiley and Sons.

However, the mechanistic picture for the cobalt-catalyzed asymmetric hydrogenation of enamides is not so simple. Contemporaneously with the 2022 (*R*,*R*)-(^*i*Pr^DuPhos) work by our group and the Hopmann lab, a second, primarily computational study was published focusing on the (*R*,*R*)-(^Ph^BPE)­Co-catalyzed asymmetric hydrogenation of prochiral enamides, including MAA and levetiracetam.^76^ With both substrates, the combined Gibbs free energy calculations and HD labeling results suggested that both pathways A and B were feasible, in contrast to the results obtained with (*R*,*R*)-(^*i*Pr^DuPhos) where only Co­(0/II) redox cycling pathways are possible. The redox-neutral cobalt­(II) metallacycle pathway (Scheme 33) begins with a Co­(II) monohydride amidate, the product of formal N–H oxidative addition to the cobalt, although this direct pathway of formation was too high in energy. Rather, assistance of proton transfer from the MeOH solvent was required for formation of the cobalt­(II) monohydride amidate. Both Co­(0/II) redox cycle A and all-Co­(II) metalla­cycle B pathways gave calculated enantio­selectivities comparable to that derived from catalytic experiments. This study highlights how the mechanistic picture for cobalt catalysis can become quite complicated when substrates may access both pathways A and B, and that the chosen ligand seems to have a major effect on the mechanistic outcome.

**Scheme 33:**
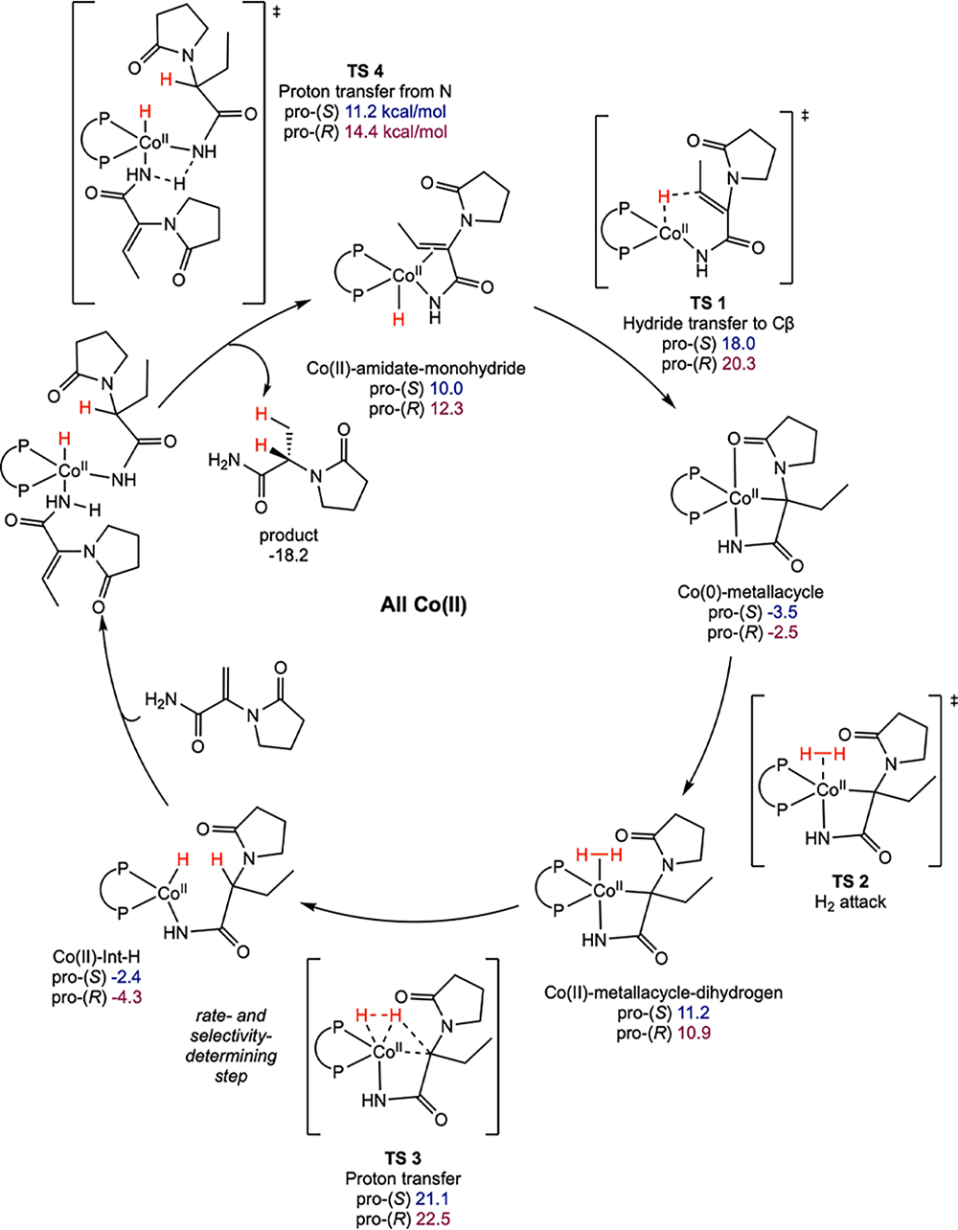
DFT-Calculated All-Co­(II) Metallacycle Mechanism B for the Hydrogenation of *dehydro*-Levetiracetam (Hopmann and Chirik, 2022 ^76^)­^a^

a Adapted from ref 76. Copyright 2022 American Chemical Society.

In contrast to these studies, in 2023, de Vries and co-workers supported a third mechanism for the cobalt-catalyzed asymmetric hydrogenation of cyclic enamides.^71^ A combination of (*S*,*S*)-^Ph^BPE and CoCl_2_ was employed in the absence of zinc or other metal reductants under relatively high (60 atm) hydrogen pressures at 60 °C. With CoCl_2_ as the cobalt source, the active catalyst was formed only in methanol, as sluggish reactions were observed in other protic solvents. Deuterium labeling studies demonstrated that the source of hydrogen is from the gas and not the protic solvent, and that migratory insertion is irreversible. Significant efforts were made to rule out nanoparticle formation during the reaction. Reaction progress monitoring experiments demonstrated a somewhat sinusoidal behavior attributed to both substrate and product inhibition. EPR spectroscopy established the formation of high-spin cobalt­(II) complexes following mixing of the phosphine, CoCl_2_, and substrate in MeOH at 60 °C. Repeating this experiment under 10 bar of H_2_ generated a similar signal. Running a standard hydrogenation in a high-pressure autoclave, followed by EPR measurement, again established the formation of high-spin Co­(II) compounds. These combined results led to the conclusion that Co­(II) is involved as the active species in the hydrogenation reaction. A mass spectroscopy experiment was also performed in which CoCl_2_ and (*S*,*S*)-^Ph^BPE were stirred in MeOH in the presence of substrate at 60 °C but in the absence of H_2_. The subsequent reaction mixture was then frozen and analyzed by mass spectrometry, which showed signals that matched with the masses of (*S*,*S*)-^Ph^BPECo­(Cl) and (*S*,*S*)-^Ph^BPECo­(Cl)­(substrate). Based on these findings, the authors propose an all Co­(II) cycle, wherein a single chloride ligand remains bound to the cobalt throughout the catalytic cycle (Scheme 34).

**Scheme 34:**
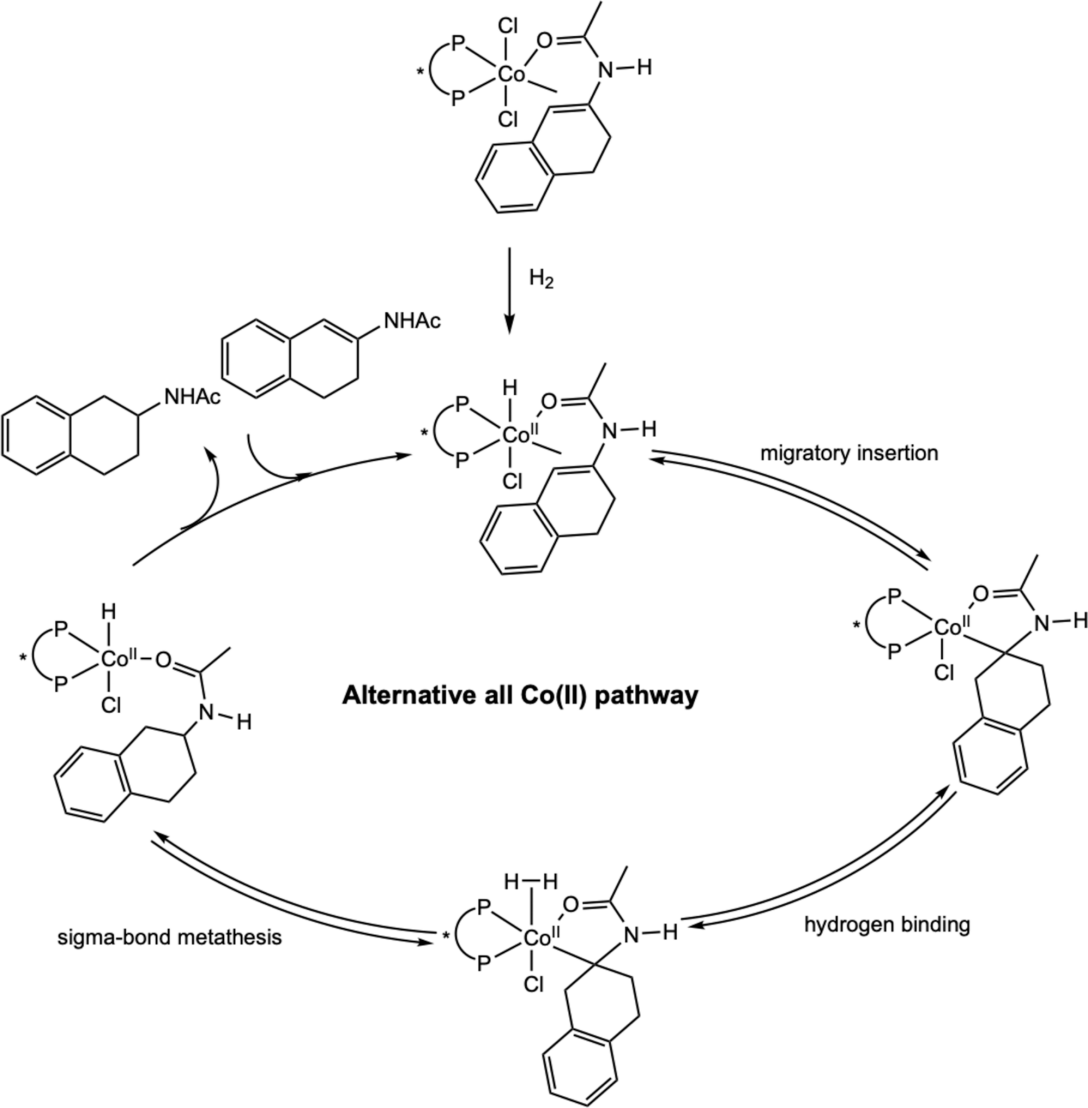
Proposed Mechanism for the Asymmetric Hydrogenation of Cyclic Enamides with CoCl_2_ in the Absence of Zinc (de Vries, 2023 ^71^)­^a^

a Adapted from ref 71. Available under a CC-BY-NC-ND license. Copyright 2023 Soumyadeep Chakrabortty, Katharina Konieczny, et al.

Taken together, these results demonstrate a much more complex network of potential pathways for asymmetric enamide hydrogenation when utilizing cobalt precatalysts as compared to their rhodium counterparts. As the field is still in its infancy, major questions persist, including how cobalt­(II) dihalide precatalysts are activated in the absence of metal reductant and the specific ligand features that determine the preferred mechanistic pathway.

### Mechanistic Results in Neutral Cobalt-Catalyzed Asymmetric Hydrogenation of Alternative Substrates

Aside from enamides, the cobalt-catalyzed asymmetric hydrogenation of α,β-unsaturated carboxylic acids have been the most extensively studied, and here the mechanistic picture seems more well-defined. An all-Co­(II) metallacycle pathway B is supported for the asymmetric hydrogenation of α,β-unsaturated carboxylic acids on the basis of results published by Zhang and co-workers and by our laboratory.^66–68^ The asymmetric hydrogenation of this class of substrates by cobalt complexes was independently published by the two laboratories in the same time period, followed by another study by the Zhang group on the asymmetric hydrogenation of tetrasubstituted variants. The Zhang lab found that no zinc activator was required when Co­(acac)_2_ was used as the cobalt source. While the hydrogenation of α,β-unsaturated carboxylic acids produced high yields and enantio­selectivities, the corresponding ethyl ester gave no conversion even in the presence of exogenous AcOH or carboxylic acid substrate. These results indicate that the carboxyl group of the substrate coordinates to the cobalt during the course of the reaction. EPR experiments established a Co­(II) resting state. Similar freeze-quench EPR experiments by our laboratory also showed a Co­(II) resting state, assigned to be the bis­(phosphine) cobalt­(II) bis­(carboxylate) substrate complex (Scheme 35A). Such compounds were independently synthesized by reaction of bis­(phosphine)­Co­(COD) with H_2_ in the presence of α,β-unsaturated carboxylic acid, and characterized by X-ray diffraction. Interestingly, use of the Co­(II) bis­(carboxylate) substrate complexes as precatalysts produced comparably high conversion, but greatly diminished enantio­selectivity as compared to well-defined Co(0) precatalysts, suggesting a different mechanism for enantio­induction, although the origin of this difference has not been elucidated. The Zhang lab found that deuterium labeling using D_2_ in MeOH resulted in 1:1 deuterium incorporation in both the α- and β-positions of the product, consistent with homolytic cleavage of H_2_ with no evidence for solvent participation, which is common in precious metal catalysis.^77^ Similarly, when well-defined (*R*,*R*)-(^Ph^BPE)­Co­(COD) was used by our laboratory for reaction with D_2_ in MeOH, 1:1 incorporation of the isotopic label was also observed (Scheme 35B). However, when this experiment was repeated with either (*R*,*R*)-(^*i*Pr^DuPhos)­Co­(OPiv)_2_ or (*R*,*R*)-(BenzP*)­Co­(OPiv)_2_ ((*R*,*R*)-BenzP* = (*R*,*R*)-(+)-1,2-bis­(*tert*-butylmethyl­phosphino)­benzene) as the precatalyst, 15–20% deuterium incorporation was observed in one of the substrate carbons. This further suggests a potential difference in catalyst activation modes between the Co(0) and Co­(II) bis­(carboxylate) complexes.

**Scheme 35:**
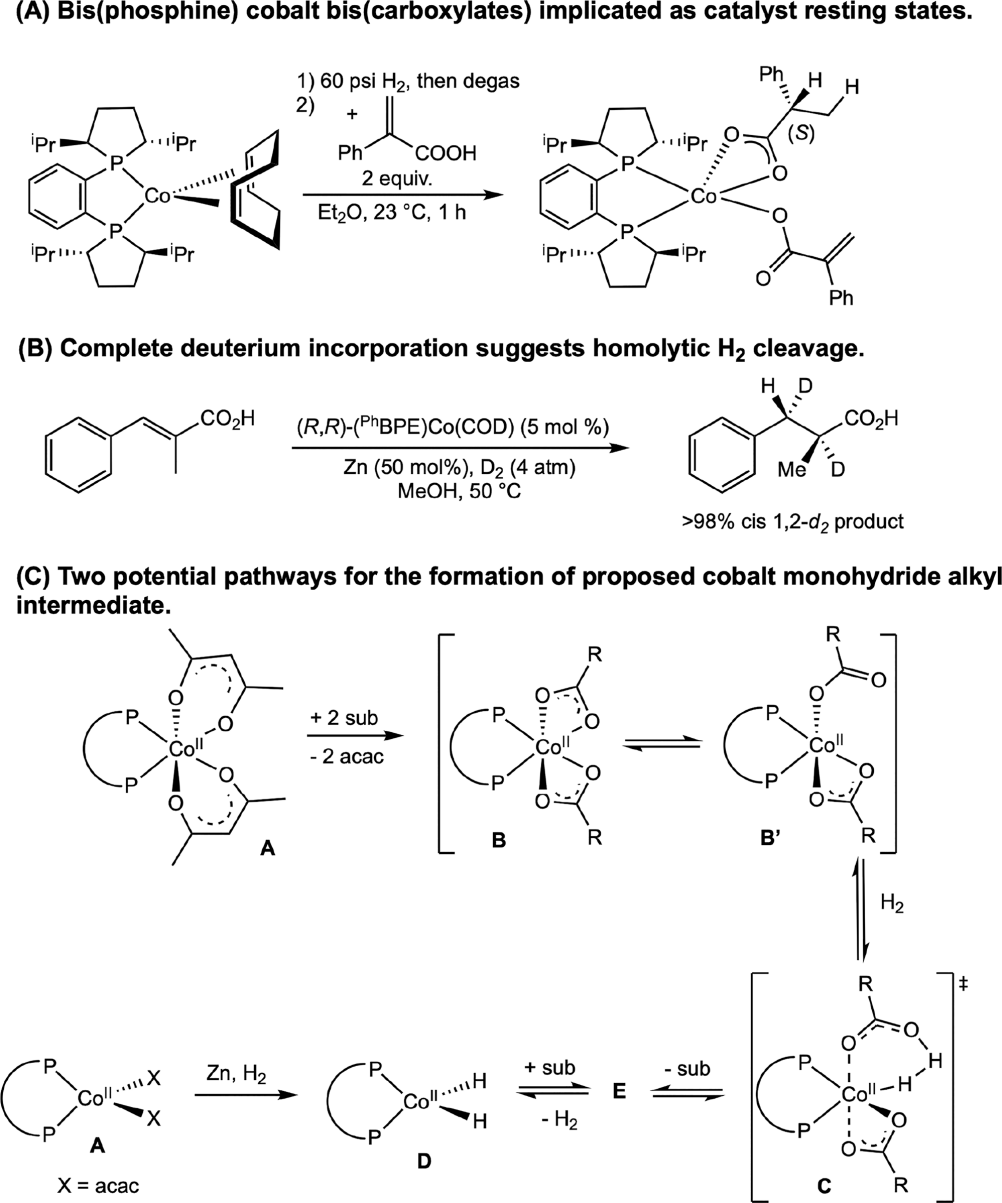
(A) Independent Synthesis of Cobalt­(II) Bis­(carboxylate) Complexes Identified as Resting States during Asymmetric Hydrogenation, (B) Deuterium Labeling Experiments Establishing Homolytic H_2_ Cleavage, and (C) Proposed Pathway for the Formation of the Key Monohydride Intermediate (Chirik, 2020,^66^ and Zhang, 2020 ^67^)­^a^

a Adapted from ref 66. Copyright 2020 American Chemical Society. Adapted from ref 67. Available under a CC-BY 4.0 license. Copyright 2020 Xiaoyong Du, Ye Xiao, et al.

On the basis of their results, the Zhang laboratory proposed a redox-neutral Co­(II) pathway B involving metalla­cycles. This mechanism commences from a cobalt­(II) carboxylate monohydride where the substrate is coordinated to the cobalt through both the carboxylate oxygen and the C=C bond. Two pathways are proposed for the generation of the initial cobalt­(II) carboxylate monohydride from a bis­(carboxylate) compound or directly from Co­(acac)_2_ with Zn and hydrogen (Scheme 35C). In their follow-up study on tetrasubstituted α,β-unsaturated carboxylic acids, a full DFT study of the mechanism was also conducted.^70^ The authors concluded that the mechanism proposed in their earlier work was plausible and initiates from a cobalt­(II) carboxylate monohydride (Scheme 36). Preferential insertion of hydrogen at the C1 atom of the substrate in a rate-, regio-, and selectivity-determining step forms the cobalt­(II) metallacycle proposed in the previous study. Such a pathway predicts an overall *ee* of 94.4%, which is in excellent agreement with the experimentally determined value of 90%.

**Scheme 36:**
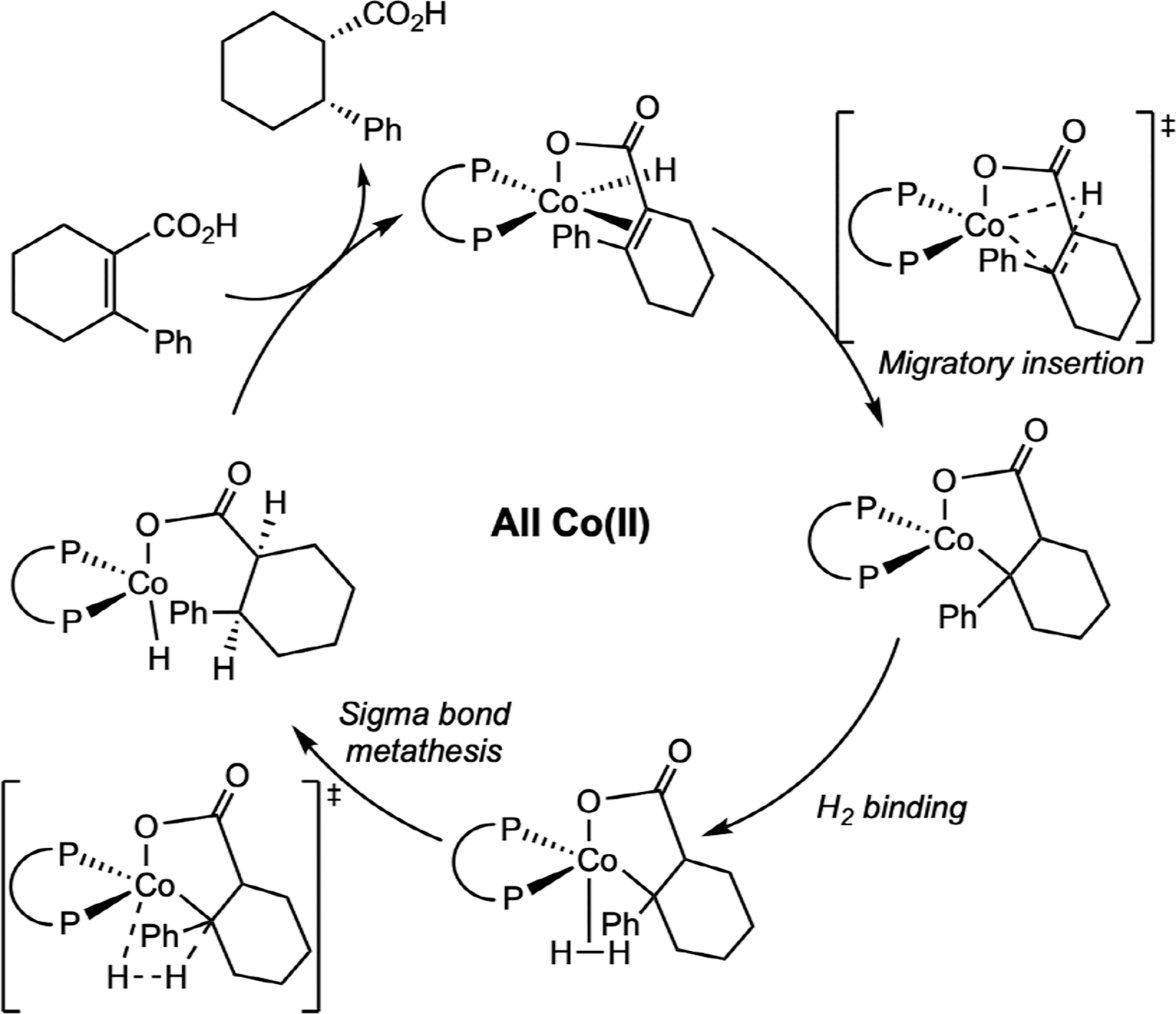
Computationally Supported Cobalt­(II) Metallacycle Pathway B for the Hydrogenation of Tetrasubstituted α,β-Unsaturated Carboxylic Acids (Zhang, 2021 ^68^)­^a^

a Adapted with permission from ref 68. Copyright 2021 John Wiley and Sons.

A Co­(0/II) redox cycling pathway has also been proposed by Wanbin Zhang and co-workers in the azole-directed hydrogenation of alkenes using in situ generation from CoCl_2_ and (*S*,*S*)-^Ph^BPE in the presence of zinc reductant.^72^ This publication is the first defined example of a nitrogen atom acting as a directing group for bis­(phosphine) cobalt-catalyzed asymmetric alkene hydrogenation. In contrast to each of the previous examples, the azole-containing alkenes possess no X–H bond to allow for pathway B, constraining the mechanistic possibilities. Interestingly, zinc was not required for hydrogenations conducted at higher (≥10 atm) hydrogen pressures, but a profound effect on added activator was noted at lower (1 atm) pressure. Deuterium labeling studies established that H_2_ is the hydrogen source, rather than protons from the alcohol solvent. Higher levels of deuterium incorporation in the methyl carbon of the product were observed as compared to the prochiral position, suggesting that insertion into the Co–H occurs in a 2,1-fashion and is reversible, a distinct difference from the proposed mechanism for enamide hydrogenation (Scheme 37A). On the basis of these results, the authors proposed the Co­(0/II) redox-cycling pathway shown in Scheme 37B.

**Scheme 37:**
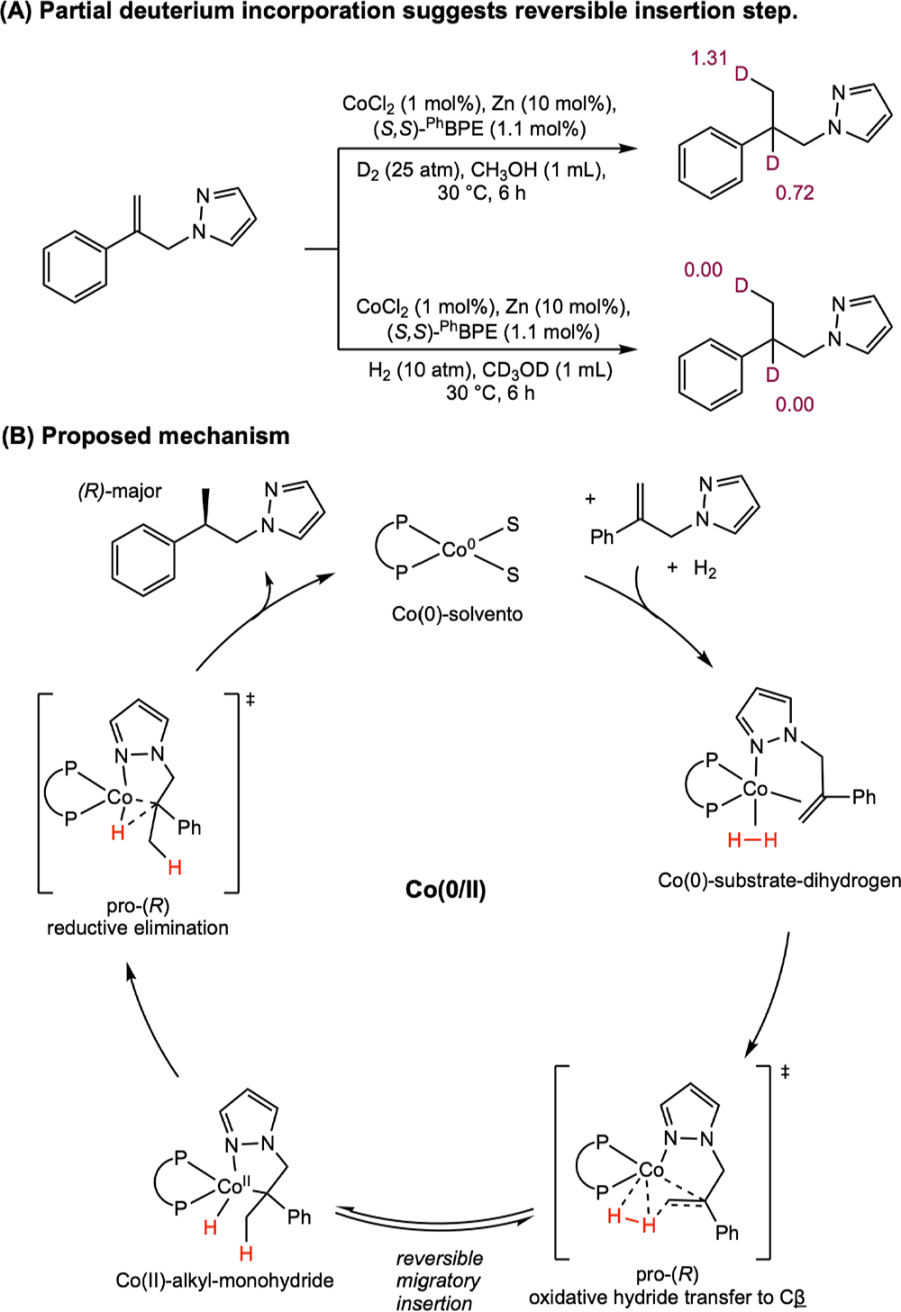
Azole-Directed Asymmetric Hydrogenation: (A) Deuterium Labeling Experiments and (B) Proposed Catalytic Cycle (Zhang, 2022 ^72^)­^a^

a Adapted with permission from ref 72. Copyright 2022 John Wiley and Sons.

### Mechanistic Studies with Cationic Cobalt Precatalysts for Asymmetric Alkene Hydrogenation

Our laboratory has also reported the synthesis of well-defined cationic cobalt­(I) complexes and their application in the asymmetric hydrogenation of prochiral enamides,^65^ as well as the block-buster diabetes treatment, Januvia.^78^ The cationic cobalt­(I) complexes were accessed through stepwise one-electron reduction to the bridging chloride dimer, [bis­(phosphine)­CoCl]_2_, followed by salt elimination with NaBAr^F^_4_ in the presence of coordinating ligand. These cationic cobalt complexes are diamagnetic and are analogous to the bis­(phosphine) rhodium­(I) cationic complexes historically utilized in the mechanistic studies of rhodium-catalyzed asymmetric alkene hydrogenation. Independent synthesis of [(*R*,*R*)-(^*i*Pr^DuPhos)­Co­(MAA)]­[BAr^F^_4_] demonstrated exclusive formation of the “wrong” pro-(*R*) diastereomer, as compared to the (*S*) hand of the product that is strongly favored in catalysis, even upon cooling to low temperatures (Scheme 38A). Hydrogenation of this pro-(*R*) diastereomer gave >99% conversion and >99% *ee* of the (*S*) product. This suggests that an anti-“lock-and-key” mechanism is also operative with the cationic cobalt­(I) catalysts (Scheme 38B).

**Scheme 38:**
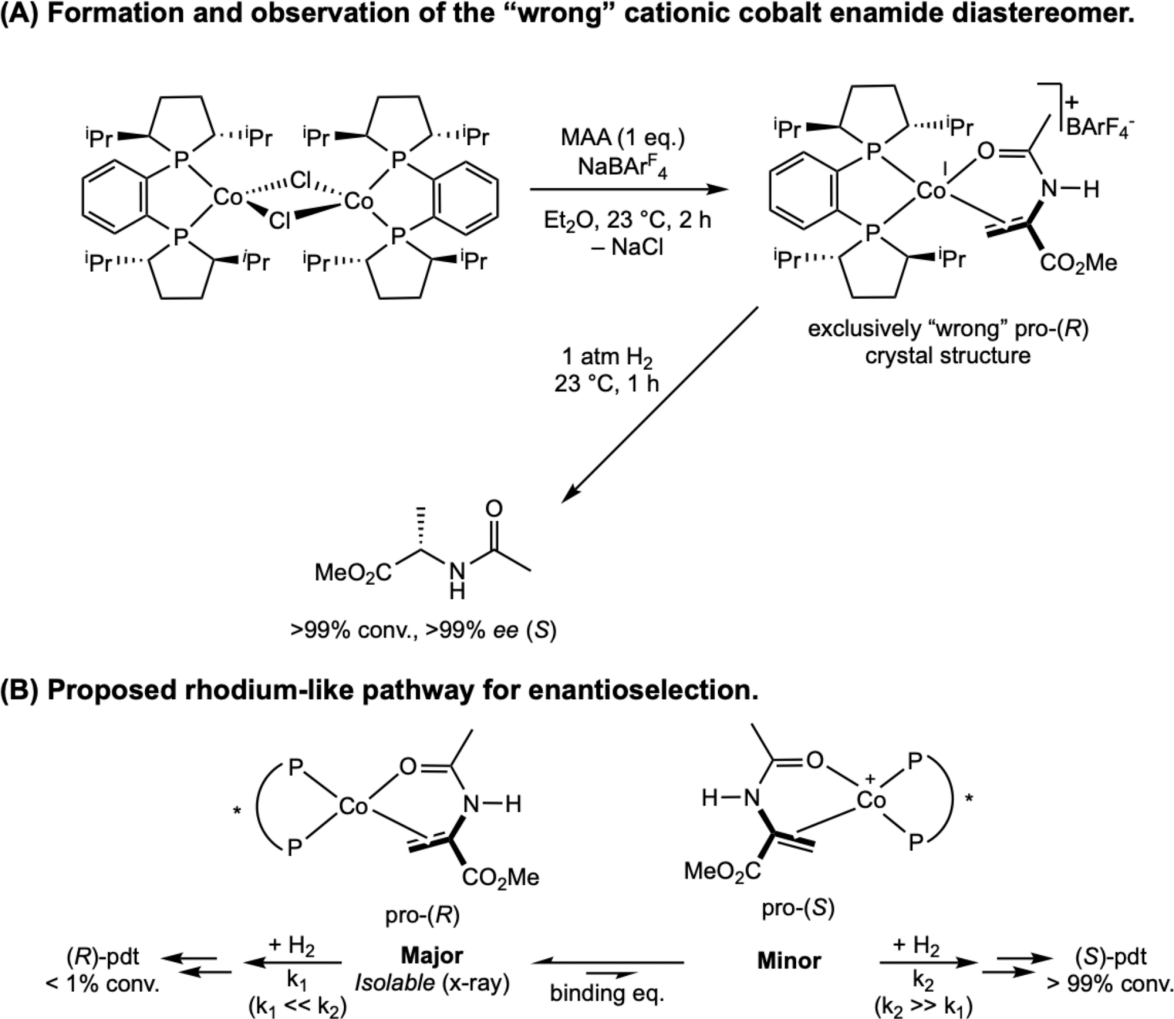
(A) Independent Synthesis and Hydrogenation of [(*R*,*R*)-(^*i*Pr^Duphos)­Co­(MAA)]­[BAr^F^_4_] Demonstrating Exclusive Formation of the Pro-(*R*) Diastereomer and Hydrogenation to Exclusively the (*S*)-Product and (B) Proposed Curtin–Hammett Scheme for Explanation of These Results (Chirik, 2019 ^65^)­^a^

a Adapted with permission from ref 65. Copyright 2019 John Wiley and Sons.

A subsequent study comparing cyclooctadiene ligand exchange in neutral and cationic bis­(phosphine) cobalt complexes demonstrated a much faster rate of ligand exchange with the cationic complex.^79^ This was attributed to the square planar geometry around the [(*R*,*R*)-(^*i*Pr^DuPhos)­Co­(COD)]­[BAr^F^_4_] complex, allowing for a faster rate of associative ligand exchange. In contrast, neutral (*R*,*R*)-(^*i*Pr^DuPhos)­Co­(COD) adopts a paramagnetic square pyramidal geometry. Significant back-bonding from the neutral cobalt into one of the COD C=C bonds renders it more similar to a Co­(II) metallacycle. Kinetic experiments support a dissociative mechanism, whereby rate-determining dissociation of a single C=C bond from cobalt center is required prior to association of a second equivalent of COD. This study demonstrates how the one-electron flexibility of cobalt oxidation states may play a role in their respective mechanisms ligand substitution and ultimately catalytic alkene hydrogenation. However, the mechanism of cationic cobalt-catalyzed asymmetric hydrogenation has been largely understudied and significant questions remain.

### Summary of Mechanistic Studies in Cobalt-Catalyzed Hydrogenation of Functionalized Alkenes

While bis­(phosphine) cobalt-catalyzed asymmetric hydrogenation has been less well studied as compared to that of its rhodium- and iridium-catalyzed counterparts, the experimental and computational studies conducted thus far have begun to paint a mechanistic picture. In the few cases examined, cobalt complexes tend to follow unsaturated pathways, whereby reaction with substrate occurs prior to the reaction with H_2_. Neutral cobalt complexes, in the presence of substrates bearing proximal X–H bonds, are prone to follow the directed redox-neutral Co­(II) metalla­cycle mechanism B, which has been suggested for alcohols, carboxylic acids, and enamides. Redox cycling Co­(0/II) pathways are also possible, particularly with substrates lacking X–H bonds and enamides. The factors which dictate the choice of pathway have yet to be fully elucidated, and doing so will likely require more detailed studies with additional substrate classes. Only a very small subset of ligands has been mechanistically studied with cobalt, and if a broader range of ligands are explored, different mechanistic pathways may result. The ability to access cobalt complexes in both neutral and cationic oxidation states offers the possibility for new mechanistic pathways and substrate scope.

## Conclusions

Overall, the rich history and literature on the mechanisms of group 9 metal-catalyzed asymmetric hydrogenation paints a complex but comprehensible mechanistic picture, following distinct guiding principles. While each metal possesses its own mechanistic nuances, differences can be attributed to the available oxidation states and the coordination affinities of each metal center. Rhodium follows the most simplified mechanistic picture, following a distinct Rh­(I)–Rh­(III) redox cycle comprised of well-known elementary steps such as ligand substitution, oxidative addition, reductive elimination, and migratory insertion. In the case of iridium, the ability to access Ir­(I), Ir­(III), and Ir­(V) intermediates creates a more complex but interesting picture. The high coordination affinity of the iridium center also allows productive catalysis with minimally functionalized olefins which do not have the ability to engage in two-point binding that is familiar in the mechanism of rhodium-catalyzed asymmetric hydrogenation and a source of enantio­selection. As the emphasis on less expensive and more terrestrially available metals increases, the interest in the mechanism of cobalt-catalyzed alkene asymmetric hydrogenation will also increase. Notably, these catalysts rank among the most active and enantio­selective known. The substitutional lability of cobalt, as well as the ability of this metal to access a range of single-electron differentiated oxidation states, creates a rich mechanistic landscape. While rhodium-like two-electron pathways are possible, additional mechanisms have been supported with intermediates in multiple oxidation states. Mechanistic studies on all three metals help inform chemists about the nature of transition-metal activity in one of the most important reactions in homogeneous catalysis.
